# Machine Learning Enables Accurate and Rapid Prediction of Active Molecules Against Breast Cancer Cells

**DOI:** 10.3389/fphar.2021.796534

**Published:** 2021-12-17

**Authors:** Shuyun He, Duancheng Zhao, Yanle Ling, Hanxuan Cai, Yike Cai, Jiquan Zhang, Ling Wang

**Affiliations:** ^1^ Guangdong Provincial Key Laboratory of Fermentation and Enzyme Engineering, Guangdong Provincial Engineering and Technology Research Center of Biopharmaceuticals, School of Biology and Biological Engineering, South China University of Technology, Guangzhou, China; ^2^ Joint International Research Laboratory of Synthetic Biology and Medicine, Guangdong Provincial Engineering and Technology Research Center of Biopharmaceuticals, School of Biology and Biological Engineering, South China University of Technology, Guangzhou, China; ^3^ Center for Certification and Evaluation, Guangdong Drug Administration, Guangzhou, China; ^4^ State Key Laboratory of Functions and Applications of Medicinal Plants, College of Pharmacy, Guizhou Provincial Engineering Technology Research Center for Chemical Drug R&D, Guizhou Medical University, Guiyang, China

**Keywords:** breast cancer, machine learning, graph neural networks, molecular fingerprints, structural fragments

## Abstract

Breast cancer (BC) has surpassed lung cancer as the most frequently occurring cancer, and it is the leading cause of cancer-related death in women. Therefore, there is an urgent need to discover or design new drug candidates for BC treatment. In this study, we first collected a series of structurally diverse datasets consisting of 33,757 active and 21,152 inactive compounds for 13 breast cancer cell lines and one normal breast cell line commonly used in in vitro antiproliferative assays. Predictive models were then developed using five conventional machine learning algorithms, including naïve Bayesian, support vector machine, k-Nearest Neighbors, random forest, and extreme gradient boosting, as well as five deep learning algorithms, including deep neural networks, graph convolutional networks, graph attention network, message passing neural networks, and Attentive FP. A total of 476 single models and 112 fusion models were constructed based on three types of molecular representations including molecular descriptors, fingerprints, and graphs. The evaluation results demonstrate that the best model for each BC cell subtype can achieve high predictive accuracy for the test sets with AUC values of 0.689–0.993. Moreover, important structural fragments related to BC cell inhibition were identified and interpreted. To facilitate the use of the model, an online webserver called ChemBC (http://chembc.idruglab.cn/) and its local version software (https://github.com/idruglab/ChemBC) were developed to predict whether compounds have potential inhibitory activity against BC cells.

## 1 Introduction

According to the latest data on the global cancer burden for 2020 released by the International Agency for Research on Cancer of the World Health Organization, breast cancer (BC) surpassed lung cancer in 2020 to become the most common cancer worldwide. BC is the leading cause of cancer-related death among women worldwide (Sung et al., 2021). BC consists of the uncontrolled proliferation of mammary epithelial cells under the action of many carcinogenic factors (Escala-Garcia et al., 2020), including alcohol consumption, smoking, overweight, and mammographic density. BC is classified according to the expression of the estrogen receptor (ER), progesterone receptor (PR), human epidermal growth factor receptor 2 (HER2), and Ki-67 into five subtypes: Luminal A, Luminal B (HER2-positive or HER2-negative), HER2-positive, and triple-negative breast cancer (TNBC) (Harbeck et al., 2013). Among these BC subtypes, TNBC is associated with poor survival mediated by treatment resistance, and it is the most difficult to treat with curative intent (Liao et al., 2021). Several drugs (e.g., anthracyclines and trastuzumab) have been approved by the U.S. Food and Drug Administration (FDA) for the treatment of BC; however, issues such as poor efficacy, toxicity, adverse drug reactions, and the emergence of drug resistance have limited their clinical use (Brower, 2013; Cameron et al., 2017; Shah and Gradishar, 2018; Daniyal et al., 2021; Li and Li, 2021). Therefore, there is an urgent need to discover and develop new drugs for the treatment of BC, particularly for TNBC.

Innovative drugs (or active molecules) can be identified through two mainstream screening methods: phenotypic-based screening and target-based screening. Target-based screening has been widely used to discover new drugs for the treatment of human diseases in both the pharmaceutical industry and academia for more than 30 years (Chen et al., 2014; Zhang et al., 2014; Wang et al., 2017a; Luo and Wang, 2017; Moffat et al., 2017; Shang et al., 2017). Target-based screening has several advantages, including simplicity, lower cost, and easy to achieve efficient structure-activity relationship (SAR) for lead optimization (Croston, 2017). However, there are two major concerns associated with target-based approaches: 1) the identification and validation of druggable targets is difficult, and if a selected target is undruggable, it may lead practitioners to pursue projects and compounds that fail to translate into clinical results (Croston, 2017) and 2) the conventional “one drug, one target” paradigm has shown unsatisfactory clinical results in human complex diseases (e.g., cancer (Wermuth, 2004), Alzheimer’s disease (Wang et al., 2017b; Albertini et al., 2021), and infectious diseases (Morphy et al., 2004; Li et al., 2019). Phenotypic-based screening (e.g., whole-cell activity), an original but indispensable drug screening method, has gained attention in recent years because of the number of discovered and approved drugs (Liu et al., 2019; Childers et al., 2020; Berg, 2021; Quancard et al., 2021). Two influential analyses by Swinney and Anthony in 2011 and Swinney in 2013 highlighted that the majority of first-in-class drugs (new chemical entities, NME) approved between 1999 and 2008 were identified through phenotypic screening approaches compared with target-based screening methods. In reality, most FDA approvals of first-in-class drugs originated from phenotypic screening before their precise mechanisms of action or molecular targets were elucidated.

Although phenotype-based screening has advantages over target-based screening for drug discovery, it is unscalable, costly, and does not contribute to the understanding of the mechanism of action of drugs. Several important technologies including affinity-based approaches, functional genetic approaches, cellular profiling approaches, and knowledge-based (computational) approaches are currently available and can be used to characterize the direct and indirect target space of bioactive compounds from phenotypic screening (Schirle and Jenkins, 2016; Sydow et al., 2019; Hughes et al., 2021).

Increased amounts of phenotypical pharmacological data on cancer, Alzheimer’s disease, and infectious diseases have been accumulated in the past 3 decades. Inspired by the available phenotypic screening data, several efficient and cost-saving computational models have been developed to accelerate the drug design and discovery process (Zoffmann et al., 2019; Buckner et al., 2020; Chandrasekaran et al., 2021; Malandraki-Miller and Riley, 2021). For example, in 2020, Stokes et al. first reported directed message passing neural network models using a collection of 2,335 compounds for those that inhibited the growth of *Escherichia coli* (phenotype screening data) and then identified the lead compound halicin with broad-spectrum antibacterial activity (Stokes et al., 2020). Other machine learning-based models have been established to identify new agents against Methicillin-Resistant *Staphylococcus aureus* (Wang et al., 2016b), *Mycobacterium tuberculosis* (Ye et al., 2021), *Pseudomonas aeruginosa* (Fields et al., 2020), *Plasmodium falciparum* (Ashdown et al., 2020), and *Schistosoma* (Zheng et al., 2021). In the field of anticancer drug design and discovery, phenotypical whole cell-based screening methods have substantially advanced our ability to identify new anticancer drugs. In previous studies, we reported the development of computational models using integrated NCI-60 cell-based phenotype screening data to identify new anticancer agents (e.g., **G03** and **I2**) with significant inhibitory activity against various cancer cell lines (Guo et al., 2019; Luo et al., 2019). Although the reported integrated computational anticancer models provided valuable data for discovering anticancer agents, these models cannot distinguish or selectively predict specific cancer cell subtypes (such as BC and its subtypes). In addition, these prediction models have not been developed into easy-to-use tools (e.g., local software packages or online prediction platforms), which limits the use of these models by practitioners in the field.

In the present study, we expanded our earlier efforts aimed at developing reliable computational cell-based models to predict cell inhibitory activity in BC and subtypes and provided a free platform to share our models. A total of 588 cell-based models for BC and subtypes were developed using five conventional machine learning (ML) and five deep learning (DL) algorithms based on three major types of molecular descriptors, fingerprints, and graphs. We used the local outlier factor (LOF) (Breunig et al., 2000) algorithm to evaluate the applicability domain of the best model for each BC cell line and applied the SHapley Additive exPlanations (SHAP) (Lundberg and Lee, 2017; Lundberg et al., 2020) algorithm to highlight significant structural fragments. Finally, an online platform (http://chembc.idruglab.cn/) and local software (https://github.com/idruglab/ChemBC) were constructed based on reliable models to contribute to future research.

## 2 Methods

### 2.1 Dataset Collection and Preparation

All quantitative compound-cell associations (cell-based assays, assay type: F) for available BC cell lines and normal BC cell lines were collected from ChEMBL (Mendez et al., 2019) (downloaded in March 2021) after the exclusion of metastatic cell lines. Each BC cell dataset was then processed using the following steps: 1) compounds with biological activity reported as IC_50_, EC_50_, or GI_50_ were kept, whereas molecules that had no bioactivity record were removed; 2) the units of bioactivity (i.e., g/mL, M, nM) were converted into the standard unit in μM; 3) for a molecule with multiple bioactivity values, the final bioactivity value was obtained by averaging the available bioactivity records; 4) according to previous studies (Fields et al., 2020; Ye et al., 2021), compounds with bioactivity values (e.g., IC_50_, EC_50_, GI_50_) ≤10 μM were considered as active and vice versa; molecules whose labels could not be unequivocally assigned (e.g., activity <100 μM or activity >1 μM) were excluded from the dataset; 5) all molecules were processed by removing salt and optimized based on the MMFF94X force field using MOE software (version 2018) with the default parameters. Finally, 14 cell lines with the number of active molecules (actives) and inactive molecules (inactives) >50 were retained. Each cell-compound dataset was randomly split into three sub-datasets: training (80%), validation (10%), and test (10%). All datasets used for the models described in the present study are freely available at https://github.com/idruglab/ChemBC.

### 2.2 Molecular Representations Calculation

Choosing suitable molecular representations is essential for developing acceptable and robust QSAR models. To a certain extent, the molecular representation determines the upper limit of the accuracy of the model. To fully characterize the chemical information of these molecules, three distinct types of features were calculated and used, including molecular descriptors-, fingerprints-, and graph-based representations. RDKit descriptors (RDKitDes), a set of 208 descriptors, were used. Four fingerprint-based features including Morgan fingerprints (ECFP-like, 1024-bits) (Rogers and Hahn, 2010), MACCS keys (166-bits) (Durant et al., 2002), AtomParis fingerprints (2048-bits) (Carhart et al., 1985), and 2D Pharmacophore Fingerprints (PharmacoPFP, 38-bits) (Gobbi and Poppinger, 1998) were implemented. The molecular descriptor- and fingerprint-based representations were calculated using RDKit (Landrum, 2016) (version: 2020.03.1).

The molecular graph (*G*) representative consisted of two matrices for a given molecule: the *N* × *N* adjacency matrix *A*, representing a graph structure; and the *N* × *F* node-feature matrix *X*, where *N* is the number of nodes and *F* is the number of node features. The node-feature matrix contained the following atom features: atom type, formal charge, hybridization, number of bound hydrogens, aromaticity, number of degrees, number of hydrogens, chirality, and partial charge. The edge representation contained bond type, whether the atoms in the pair are in the same ring, whether the bond is conjugated or not, and stereo configuration of a bond (Kearnes et al., 2016). Most of them were encoded in a one-hot manner into a molecular graph. In this study, molecular graph-based representations were generated using Deepchem (version: 2.5.0). For example, the MolGraphConvFeatureizer module was used to calculate the molecular graphs of Attentive FP, GAT, and MPNN models, and the ConvMolFeaturizer (Duvenaud et al., 2015) module was used to calculate the molecular graph of the GCN model.

### 2.3 Machine Learning Algorithms and Model Construction

Five conventional ML algorithms (i.e., RF, SVM, XGBoost, KNN, and NB) and five DL algorithms (i.e., DNN, GCN, GAT, MPNN, and Attentive FP) were used to develop classification models for discriminating actives from inactives against breast cell lines. The RF, SVM, KNN, and NB models were constructed using the Scikit-learn (Pedregosa et al., 2011) python package (https://github.com/scikit-learn/scikit-learn, version: 0.24.1); the XGBoost (Chen and Guestrin, 2016) models were developed using the XGBoost python package (https://github.com/dmlc/xgboost, version: 1.3.3); and other graph-based models were established using the DeepChem python package (https://deepchem.io/). All descriptor- and fingerprint-based models and graph-based DL models were trained on CPU [Intel(R) Xeon(R) Silver 4216 CPU @ 2.10 GHz] and GPU [NVIDIA Corporation GV100GL (Tesla V100 PCIe 32 GB)], respectively. In addition, we used grid search to optimize hyperparameters for each model. Detailed these modeling methods and their hyperparameters are briefly described as follows.

#### 2.3.1 Random Forest

RF is a representative ensemble learning approach. It establishes a classifier or regressor by an ensemble of individual decision trees and makes predictions as final output by vote or by averaging multiple decision trees (Svetnik et al., 2003). Compared with a decision tree, RF has high prediction accuracy, good tolerance to outliers and noise, and is not easy to overfit. To obtain the best RF model, the following five hyperparameters were optimized: n_estimators (10–500), criterion (“*gini*” and “*entropy*”), max_depth (0–15), min_samples_leaf (1–10), and max_features (“*log2*”, “*auto*” and “*sqrt*”).

#### 2.3.2 Support Vector Machine

SVM is a supervised ML algorithm that can be used for both classification and regression tasks (Zernov et al., 2003). The basic idea underlying SVM is to find the optimal hyperplane in the feature space that can be obtained by maximizing the boundary between classes in N-dimensional space, which distinguishes objects with different class labels. SVM has been widely used in drug discovery-relevant applications such as compound activity and property prediction (Heikamp and Bajorath, 2014). In the training of SVM models, two hyperparameters, Kernel coefficient (gamma, “*auto*”, 0.1–0.2) and penalty parameter C of the error term (C, from 1 to 100), were optimized.

#### 2.3.3 Extreme Gradient Boosting

XGBoost is one of the so-called ensemble learning algorithms under the Gradient Boosting framework and has achieved state-of-the-art ranking results in many ML competitions. It has been widely used in molecular property/activity prediction tasks (Jiang Z. et al., 2021; Li et al., 2021; Ye et al., 2021). Seven hyperparameters were optimized in the training of XGBoost models: learning_rate (0.01–0.1), gamma (0–0.1), min_child_weight (1–3), max_depth (3–5), n_estimators (50–100), subsample (0.8–1.0), and colsample bytree (0.8–1.0).

#### 2.3.4 K-Nearest Neighbor

The basic idea of the KNN ML algorithm (Cover and Hart, 1967) is to identify the *k* training samples closest to the test samples in the training set based on distance measures (e.g., Euclidean, Manhattan, and Jaccard distance), and to make a prediction based on the information of the *k* samples. The default distance measure Euclidean was used in this study. The following three hyperparameters were optimized: n_neighbors (1–5), p (1–2), and weight function (“uniform”, “distance”).

#### 2.3.5 Naïve Bayes

NB is a classic classification ML method based on Bayes’ theorem (Duda and Hart, 1973) and independent assumption of characteristic conditions. For a given dataset, the joint probability distribution of input and output is first learned based on the independent hypothesis of characteristic conditions. NB is also widely used in drug discovery practices (Wang et al., 2016b; Wang et al., 2016a; Wang et al., 2016b; Guo et al., 2020). Two hyperparameters were optimized: alpha (0.01–1) and binarize (0, 0.5, 0.8).

#### 2.3.6 Deep Neural Networks

DNN is a typical DL algorithm and is essentially an artificial neural network (McCulloch and Pitts, 1943) with multiple hidden layers. It consists of many independent neurons, each of which collects information from its connected neurons, and the aggregated information is then activated through a nonlinear activation function. The following key hyperparameters were optimized: dropouts (0.1, 0.2, 0.5), layer_sizes (64, 128, 256, 512) and weight_decay_penalty (0.01, 0.001, 0.0001).

#### 2.3.7 Graph Convolutional Network

GCN is a classic neural network that can use graph-structured data as input (Kipf and Welling, 2016). It is composed of graph convolution layers, a readout layer, fully connected layers, and an output layer. The core idea of graph convolution is to use edge information for aggregating node information, thereby generating a new node representation. Various GCN frameworks have been proposed. Duvenaud et al. (2015) introduced a convolutional neural network that allows end-to-end learning of prediction pipelines. In this study, we used Duvenaud’s GCN method, and the following hyperparameters were optimized: weight_decay (0, 10e-8, 10e-6, 10e-4), graph_conv_layers [(64, 64), (128, 128), (256, 256)], learning rate (0.01, 0.001, 0.0001) and dense_layer_size (64, 128, 256).

#### 2.3.8 Graph Attention Network

Attention mechanism (AM) is one component of a neural network architecture, which can be embedded in the DL models to automatically learn and calculate the contribution of input data to output data. GCN cannot complete the inductive task, namely, dynamic graph problems, and it is not easy for GCN to assign different learning weights to different neighbors. GAT (Veličković et al., 2017) introduces an AM to address the disadvantages of previous approaches based on GCN or its approximation. The weight of the features of adjacent nodes depends entirely on the features of the nodes and is independent of the graph structure. In the training of the GAT model, the following hyperparameters were optimized: weight_decay (0, 10e-8, 10e-6, 10e-4), learning rate (0.01, 0.001, 0.0001), n_attention_heads (8, 16, 32), and dropouts (0, 0.1, 0.3, 0.5).

#### 2.3.9 Message Passing Neural Network

MPNN, proposed by Gilmer et al. (2017), is a common graph neural network (GNN) framework for chemical prediction tasks. It can directly learn the molecular characteristics from the molecular diagram and is not affected by the graph isomorphism. In the training of the MPNN model, six hyperparameters were optimized: weight_decay (10e-8, 10e-6, 10e-4), learning rate (0.01, 0.001, 0.0001), graph_conv_layers [(64, 64), (128, 128), (256, 256)], num_layer_set2set (2, 3, 4), node_out_feats (16, 32, 64), and edge_hidden_feats (16, 32, 64).

#### 2.3.10 Attentive FP

Attentive FP, which was proposed by Xiong et al. (Xiong et al., 2020), is currently a state-of-the-art GNN model for molecular property prediction, and what is learned from the established model is interpretable. It allows the model to focus on the most relevant parts of the input by applying a graph AM. Herein, the main hyperparameters were optimized as follows: dropout (0, 0.1, 0.5), graph_feat_size (50, 100, 200), num_timesteps (1, 2, 3), num_layers (2, 3, 4), learning rate (0.0001, 0.001, 0.01), and weight_decay (0, 0.01, 0.0001).

### 2.4 Performance Evaluation of Models

The following classification evaluation metrics were used to evaluate the performance of the classification models: specificity (SP/TNR), sensitivity (SE/TPR/Recall), accuracy (ACC), F1-measure (F1 score), Matthews correlation coefficient (MCC), the area under the receiver operating characteristic (AUC), and Balanced accuracy (BA). These evaluation metrics are defined as follows:

$$\text{SP}=\;\frac{TN}{TN+FP}$$ (1)

$$\text{SE }=\;\frac{TP}{TP+FN}$$ (2)

$$\text{ACC }=\;\frac{TP+TN}{TP+TN+FP+FN}$$ (3)

$$\text{F}1\;=\;\frac{2\times Precision\times Recall}{Precision+Recall}\;=\;\frac{2\times TP}{2\times TP+FN+FP}$$ (4)

$$\text{MCC }=\;\frac{TP\times TN-FN\times FP}{\sqrt{\left(TP+FN\right)\times \left(TP+FP\right)\times \left(TN+FN\right)\times \left(TN+FP\right)}}$$ (5)

$$\text{BA }=\;\frac{TPR+TNR}{2}\;=\;\frac{SE+SP}{2}$$ (6)

where TP, TN, FP, and FN represent the number of true positives, true negatives, false positives, and false negatives, respectively.

### 2.5 Model Interpretation

The interpretation of complex ML models remains a challenge because ML algorithms are often a “black box”. Accordingly, we used a recently-developed model-agnostic interpretation framework termed SHapley Additive exPlanation (SHAP) to interpret the established ML models presented in this study. Inspired by the idea of cooperative game theory, the SHAP method constructs an additive explanatory model. In this model, all features are considered contributors. For each prediction sample, the model generates a predicted value, and the SHAP value is the value assigned to each feature in the sample. The greater the SHAP value, the greater the contribution of the corresponding feature to the ML model. The SHAP value is calculated as follows:

$$y_{i}=y_{\mathrm{base}}+f\left(X_{i1}\right)+f\left(X_{i2}\right)+\cdots +f\left(X_{\mathrm{ik}}\right)$$ (7)

where X_i_ represents the sample, X_ij_ represents the j feature of this sample, y_i_ represents the predicted value of the model for this sample, y_base_ represents the baseline of the entire model (usually the mean of the target variable for all samples), f (X_ij_) is the SHAP value of X_ij_. Intuitively, f (X_i1_) is the contribution value of the first feature in sample i to the final predicted value y_i_. When f (X_i1_) > 0, it indicates that this feature improves the predicted value and has a positive effect. On the contrary, it shows that this feature reduces the predicted value and has a reverse effect. Collectively, SHAP value can reflect the influence of the feature in each sample and show the positive and negative influence of the feature.

### 2.6 Model Applicability Domain

According to the principles of the Organization for Economic Co-operation and Development (OECD), it is necessary to determine the applicability domain (AD) of the QSAR model because of the limited structural diversity of the molecules used in the training dataset. From the perspective of ML, a suitable AD can prevent the prediction deviation from being too large because the feature range of the samples to be tested is too different from the training dataset samples. Therefore, effective identification of Out-of-Domain compounds is the basis for ensuring the reliability of the established model. We used the LOF algorithm (Breunig et al., 2000) to detect super-applicability domain compounds for the best model for each BC or normal breast cell line. LOF is based on the concept of local density, where the local area is given by k-nearest neighbors, whose distance is used to estimate the density. Regions of similar density can be identified by comparing the local density of an object with that of its neighbors, and points that are much lower in density than their neighbors are considered outliers.

## 3 Results

### 3.1 Dataset Analysis and Model Construction

**FIGURE 1:**
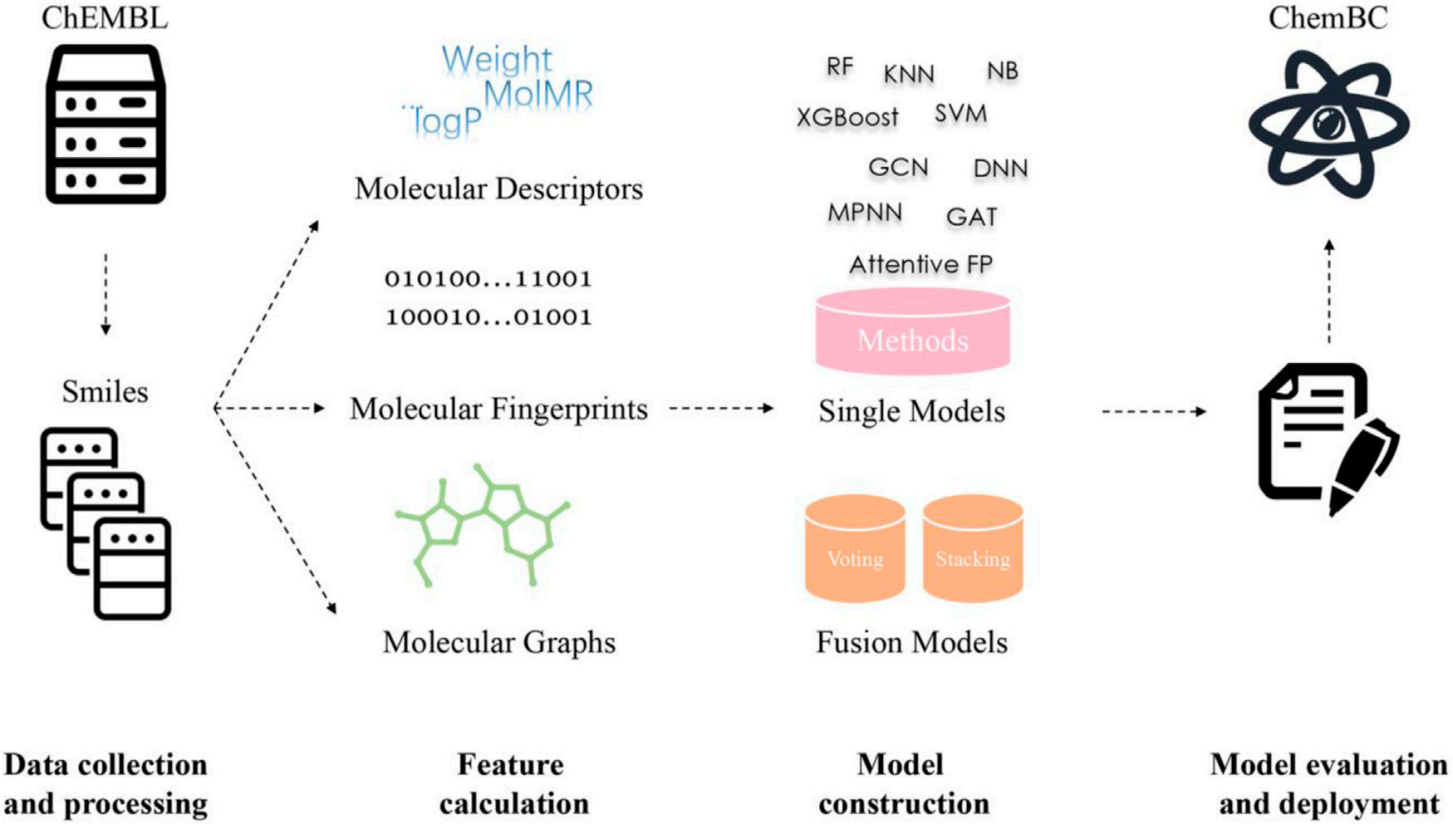
Model construction pipeline.

According to the above-predefined criteria, 14 breast-associated cell lines were obtained and distributed as follows: 1) two Luminal A subtypes including MCF-7 and T-47D; 2) two Luminal B subtypes including BT-474 and MDA-MB-361; 3) three HER-2+ subtypes including MDA-MB-435, MDA-MB-453, and SK-BR-3; 4) six TNBC subtypes including Bcap37, BT-20, BT-549, HS-578T, MDA-MB-231, and MDA-MB-468; and 5) one normal breast cell line, HBL-100. Accordingly, we selected these cell-based phenotypical datasets for subsequent modeling. The model construction pipeline is shown in Figure 1. Details on the 14 cell lines and their corresponding cell-associated compound datasets are summarized in Table 1. The compiled cell-based phenotype datasets included 34,801 unique compounds and 54,909 cell–compound associations. Among them, in 14 cell line datasets, 33,757 compounds were labeled as actives and 21,152 compounds were labeled as inactives (Supplementary Figure S1A). Supplementary Figure S1B shows the proportions of actives and inactives in the 14 cell datasets (due to the natural, although it may not be the best, we did not add theoretical decoys to deliberately balance the data), with active compounds accounting for approximately 40–78%.

**TABLE 1:** Breast cell line datasets used in this study.

Cell lines	Classification	No. of compounds	No. of scaffolds	Scaffolds/compounds (%)
MDA-MB-435	HER-2+^a^	3,030	870	28.71
MDA-MB-453	HER-2+	440	215	48.86
SK-BR-3	HER-2+	2026	571	28.18
MCF-7	Luminal A^b^	29,378	5,787	19.70
T-47D	Luminal A	3,135	926	29.54
BT-474	Luminal B^c^	811	308	37.98
MDA-MB-361	Luminal B	367	196	53.41
HBL-100	Normal cell line	316	110	34.81
Bcap37	TNBC^d^	275	73	26.55
BT-20	TNBC	292	146	50.00
BT-549	TNBC	1,182	497	42.05
HS-578T	TNBC	469	215	45.84
MDA-MB-231	TNBC	11,202	2,672	23.85
MDA-MB-468	TNBC	1986	685	34.49

a *HER-2+: HER2-positive breast cancers.

b Luminal A: Luminal A breast cancer is hormone-receptor positive (estrogen-receptor and/or progesterone-receptor positive), HER2-negative, and has low levels of the protein Ki-67, which helps control how fast cancer cells grow.

c Luminal B: Luminal B breast cancer is hormone-receptor positive (estrogen-receptor and/or progesterone-receptor positive), and either HER2 positive or HER2 negative with high levels of Ki-67.

d TNBC: triple-negative breast cancer.

The structural diversity and chemical space of compounds in datasets play a key role in the predictive ability of the ML models. Bemis–Murcko scaffold analysis (Bemis and Murcko, 1996) showed that the proportion of the scaffolds for each BC cell line dataset was between 19.70 and 53.41% (Table 1), suggesting that the anti-BC compounds of each cell line were structurally more diverse. In addition, the chemical space of the compounds in each dataset can be depicted in a two-dimensional space using molecular weight (MW) and AlogP. As shown in Supplementary Figure S2, the training, validation, and test set compounds were distributed over a wide range of MW (108.10–5,714.45) and AlogP (−55.54–42.62), demonstrating that the compounds in the modeling datasets have a broad chemical space. Based on the three different types of molecular features (i.e., molecular descriptors-, fingerprints-, and graph-based features) and the selected ten types of ML algorithms, 476 single models and 112 fusion models were developed. All models were optimized based on the validation sets and selected based on the F1 score (Kc et al., 2021). The best models were selected for the evaluation of external test datasets. The performance of the established models is discussed in the following sections.

### 3.2 Performance of Descriptor-Based Prediction Models for Breast-Associated Cells

**FIGURE 2:**
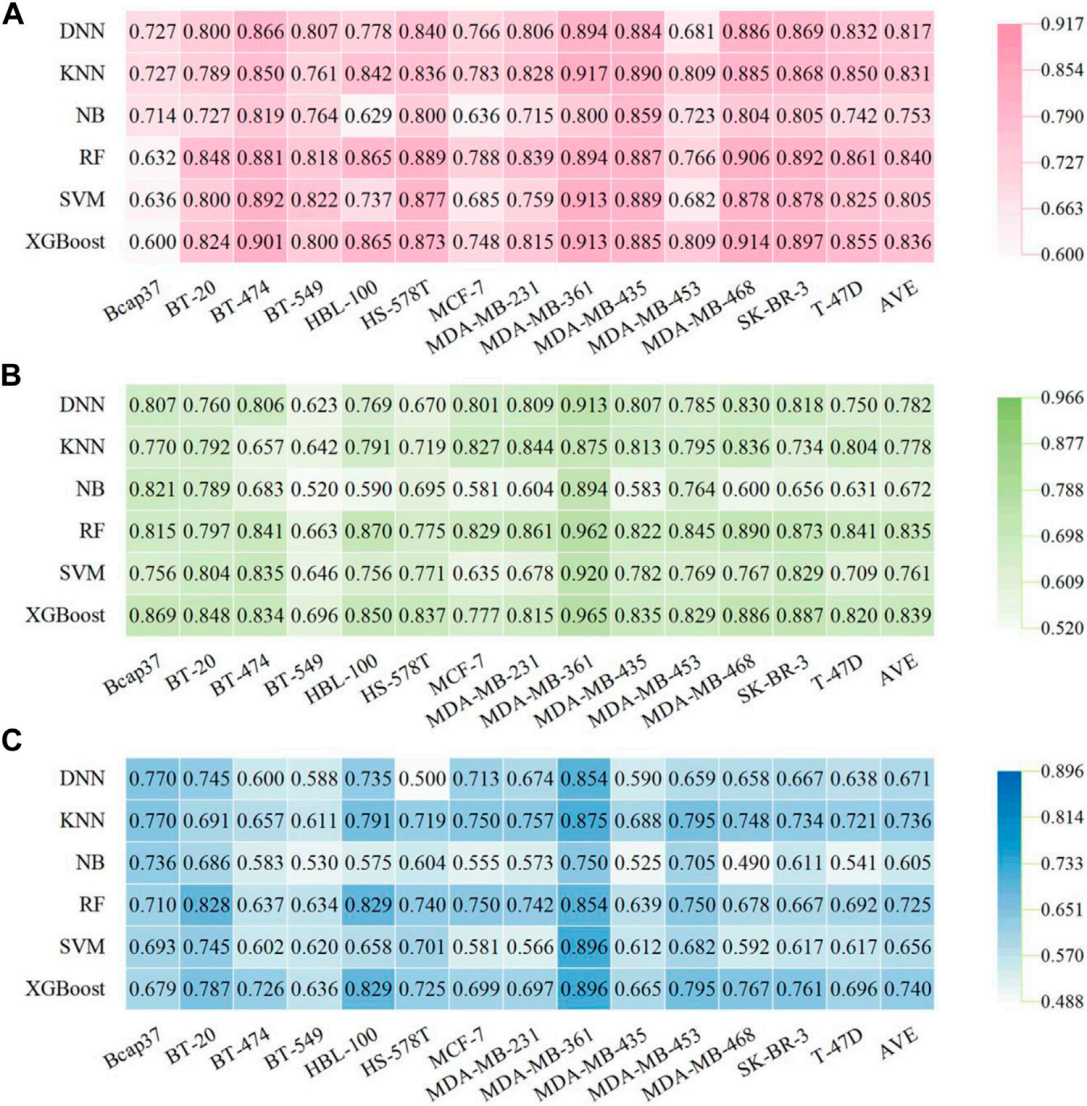
Performance of descriptor-based BC prediction models. **(A)** F1 scores of descriptor-based models. **(B)** AUC results of descriptor-based models. **(C)** BC results of descriptor-based models.

Firstly, 84 predictive models were constructed based on the RDKit-descriptors using five traditional types of ML algorithms (KNN, NB, RF, SVM, and XGBoost) and one deep learning DNN method. For these traditional ML methods, the optimized RDKit-descriptors were obtained using the SelectPercentile module (Percentile = 30) implemented in the scikit-learn package and then used as input features to construct models. Each model is denoted as a combination of a given molecular representation and ML algorithm (e.g., RF:RDKitDes). For each cell dataset and the corresponding ML methods, hyperparameters were optimized based on the validation sets (detailed in the Methods section), and the best set of hyperparameters are shown in Supplementary Table S1. The detailed performance results for descriptor-based models are listed in Supplementary Table S2. The performance of the models (F1 score, BA, and AUC) for the test sets is summarized in Figure 2. Overall, most descriptor-based models performed well in BC cell inhibitory prediction tasks, achieving a mean F1 score and BA value > 0.5. The RF model performed the best in all cell lines, with higher average F1 scores (0.840 ± 0.073), BA (0.725 ± 0.073), and AUC (0.835 ± 0.067). Meanwhile, the XGBoost model also achieved good and/or comparable performance results (Figure 2). The detailed best-performing RF:RdkitDes models results were achieved in five breast cancer cell lines (BT-20, HS-578T, MCF-7, MDA-MB-231, and T-47D), while the XGBoost:RDKitDes models also showed superior performance in five breast-associated cell lines (BT-474, HBL-100, MDA-MB-453, MDA-MB-468, and SK-BR-3). The KNN:RDKitDes models exhibited the best performance in the Bcap37, MDA-MB-361, and MDA-MB-435 cell lines. The SVM:RDKitDes models performed well in BT-549.

### 3.3 Performance of Fingerprint-Based Prediction Models for Breast-Associated Cells

**FIGURE 3:**
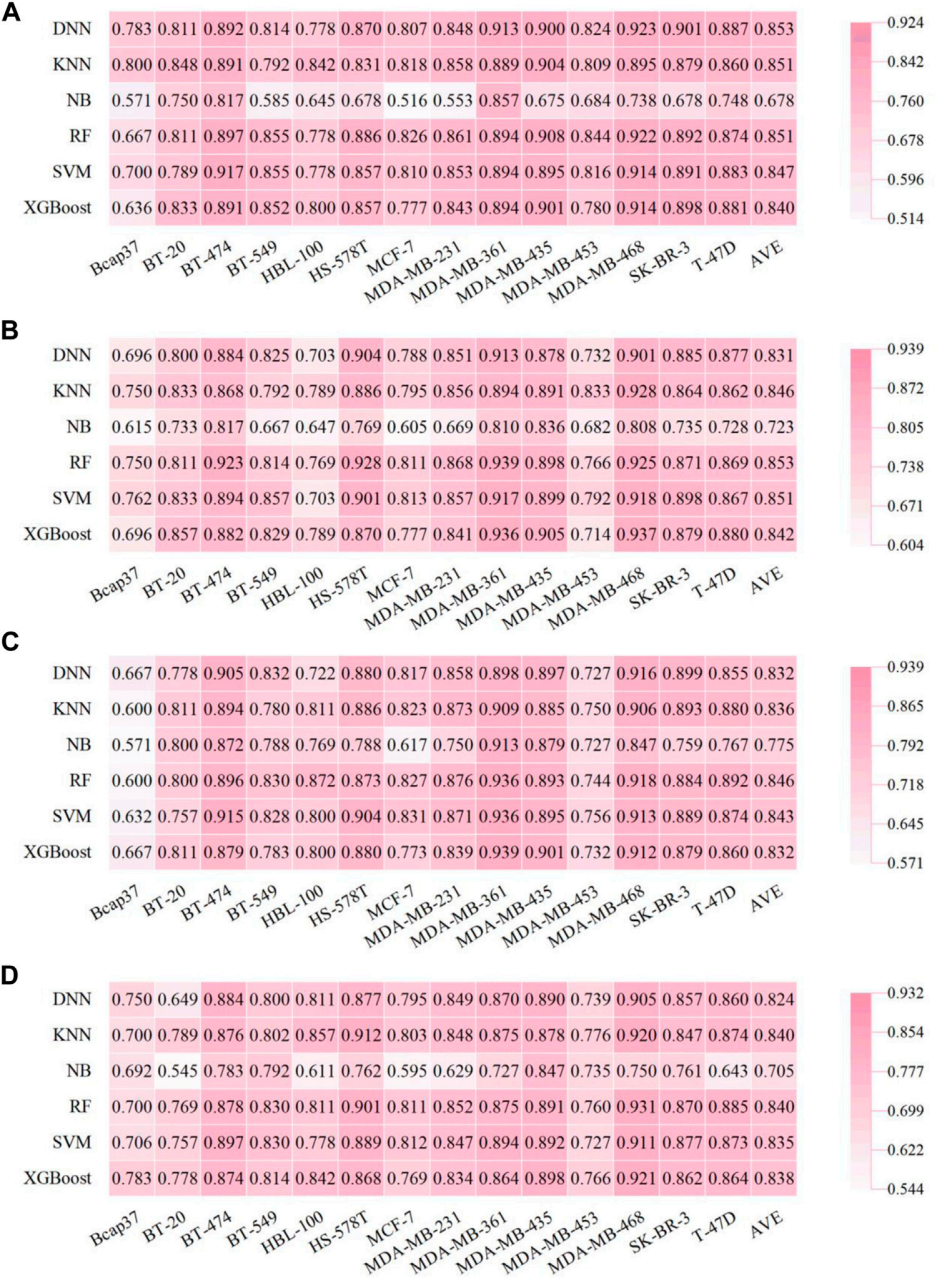
Performance of fingerprint-based BC prediction models. **(A)** F1 scores of the AtomPairs-based models. **(B)** F1 scores of the MACCS-based models. **(C)** F1 scores of the Morgan-based models. **(D)** F1 scores of the PharmacoPFP-based models.

**FIGURE 4:**
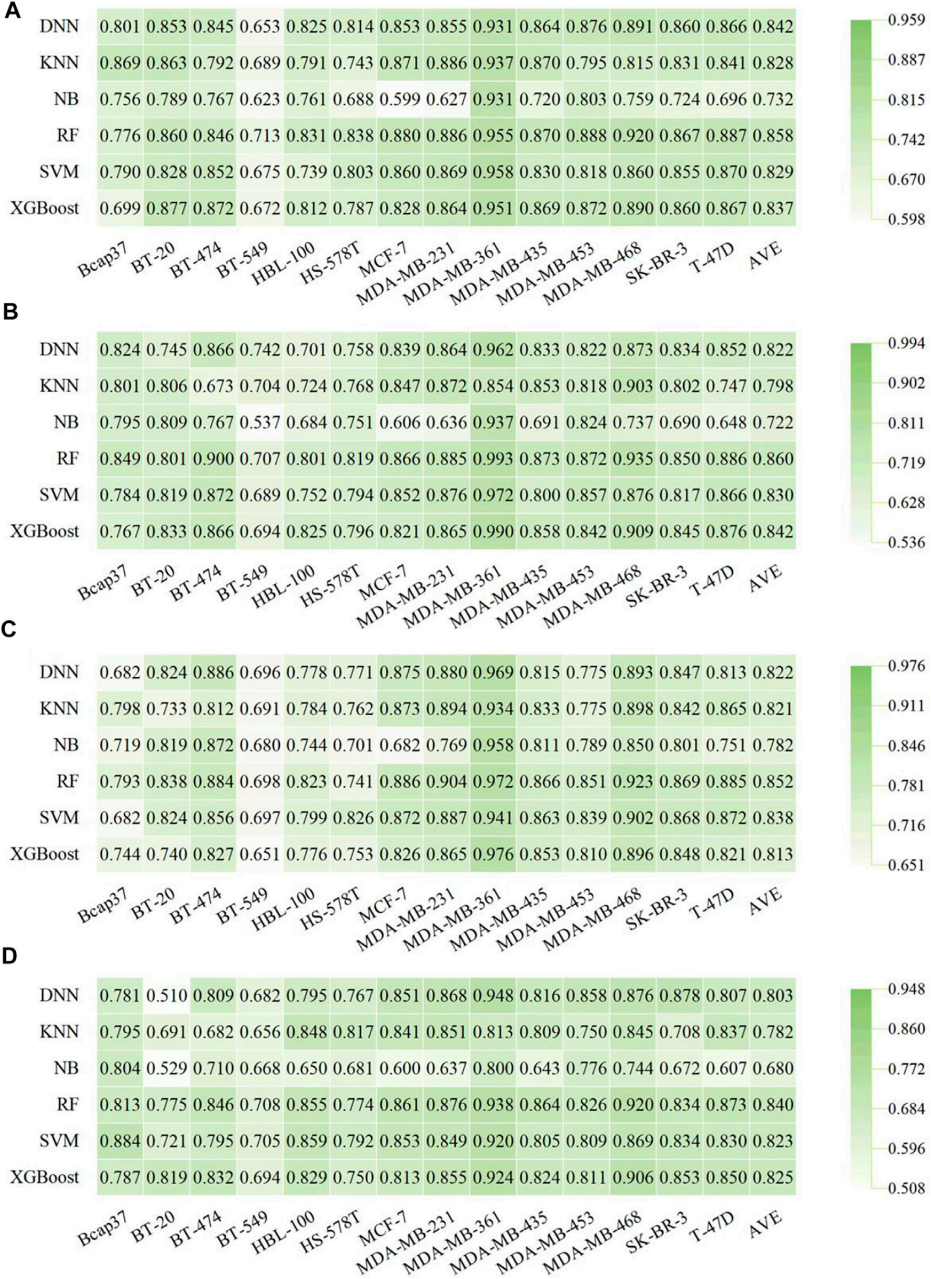
Performance of fingerprint-based BC prediction models. **(A)** AUC results of the AtomPairs-based models. **(B)** AUC results of the MACCS-based models. **(C)** AUC results of the Morgan-based models. **(D)** AUC results of the PharmacoPFP-based models.

**FIGURE 5:**
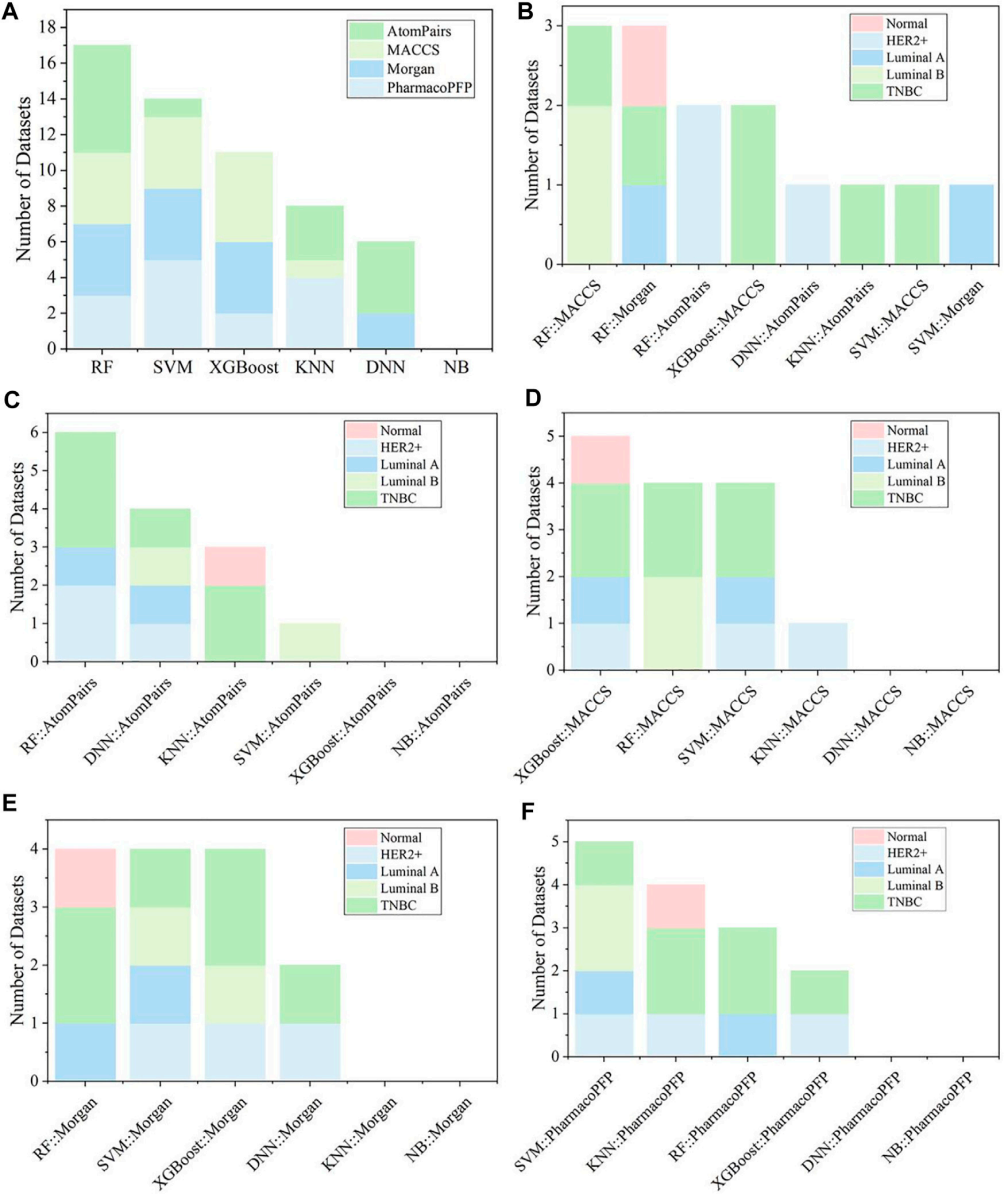
**(A)** Summary of the optimal models for each fingerprint-based feature. **(B)** The best models among various fingerprint-based models for different kinds of breast cell lines. The optimal models based on **(C)** AtomPairs, **(D)** MACCS, **(E)** Morgan, and **(F)** PharmacoPFP for different subtypes of breast cell lines.

There were 336 models developed based on four types of fingerprints (Morgan, MACCS, Atompairs, and PharmacoPFP) using six types of ML algorithms (KNN, NB, RF, SVM, XGBoost, and DNN). The detailed performance results for fingerprint-based models are listed in Supplementary Tables S3-S6. The F1, AUC, and BA values of the test sets are shown in Figures 3, 4 and Supplementary Figure S3. Taking the average F1 score as a point metric into consideration, the numbers of cell lines for which each model was identified as the best-performing are shown in Figure 5. No model, fingerprint, or ML algorithm could be identified as the best-performing for the 14 cell line datasets, demonstrating that it is necessary to screen different fingerprints and different ML algorithms for the current breast cell-associated modeling datasets (Figures 5B–F). Although the characteristics of the four molecular fingerprints are different, the RF models performed better than the other five ML models against most of the 14 cell lines (Figures 3, 4, 5A). Meanwhile, the Morgan fingerprint represents the best molecular feature representation because the ML models based on Morgan fingerprints achieved the best results for these modeling datasets (Table 2). Global analysis of four fingerprint-based models also demonstrated that RF methods can achieve a better performance than other ML methods, with the highest average F1 score (0.848 ± 0.006), BA (0.750 ± 0.013), and AUC (0.853 ± 0.009).

**TABLE 2:** Optimal models in different datasets and the evaluation of test datasets.

Molecular features	Algorithms	F1 scores^j^	BA^k^	AUC^l^
Morgan	DNN^a^	0.832 ± 0.080	0.735 ± 0.058	0.822 ± 0.078
	KNN^b^	0.836 ± 0.084	0.771 ± 0.063	0.821 ± 0.069
	NB^c^	0.775 ± 0.094	0.720 ± 0.079	0.782 ± 0.078
	RF^d^	0.846 ± 0.087	0.754 ± 0.068	0.852 ± 0.072
	SVM^e^	0.843 ± 0.084	0.747 ± 0.067	0.838 ± 0.072
	XGBoost^f^	0.832 ± 0.076	0.728 ± 0.062	0.813 ± 0.079
	Mean	0.827 ± 0.026	0.743 ± 0.019	0.821 ± 0.024
MACCS	DNN	0.831 ± 0.076	0.737 ± 0.060	0.822 ± 0.067
	KNN	0.846 ± 0.050	0.759 ± 0.056	0.798 ± 0.067
	NB	0.723 ± 0.077	0.637 ± 0.073	0.722 ± 0.103
	RF	0.853 ± 0.066	0.761 ± 0.064	0.860 ± 0.067
	SVM	0.851 ± 0.064	0.755 ± 0.059	0.830 ± 0.068
	XGBoost	0.842 ± 0.074	0.760 ± 0.056	0.842 ± 0.068
	Mean	0.824 ± 0.050	0.735 ± 0.049	0.812 ± 0.049
AtomPairs	DNN	0.853 ± 0.050	0.759 ± 0.057	0.842 ± 0.063
	KNN	0.851 ± 0.037	0.781 ± 0.051	0.828 ± 0.064
	NB	0.678 ± 0.099	0.668 ± 0.083	0.732 ± 0.085
	RF	0.851 ± 0.066	0.753 ± 0.054	0.858 ± 0.059
	SVM	0.847 ± 0.062	0.737 ± 0.069	0.829 ± 0.066
	XGBoost	0.840 ± 0.074	0.755 ± 0.041	0.837 ± 0.075
	Mean	0.820 ± 0.070	0.742 ± 0.039	0.821 ± 0.045
Molecular Graph	Attentive FP	0.831 ± 0.070	0.721 ± 0.086	0.809 ± 0.087
	GAT^g^	0.810 ± 0.086	0.695 ± 0.088	0.774 ± 0.075
	GCN^h^	0.818 ± 0.076	0.710 ± 0.091	0.798 ± 0.100
	MPNN^i^	0.821 ± 0.080	0.696 ± 0.109	0.781 ± 0.090
	Mean	0.820 ± 0.009	0.708 ± 0.011	0.793 ± 0.015
PharmacoPFP	DNN	0.824 ± 0.072	0.705 ± 0.091	0.803 ± 0.105
	KNN	0.840 ± 0.060	0.755 ± 0.075	0.782 ± 0.070
	NB	0.705 ± 0.088	0.619 ± 0.075	0.680 ± 0.080
	RF	0.840 ± 0.064	0.731 ± 0.070	0.840 ± 0.060
	SVM	0.835 ± 0.068	0.722 ± 0.064	0.823 ± 0.059
	XGBoost	0.838 ± 0.049	0.727 ± 0.072	0.825 ± 0.058
	Mean	0.814 ± 0.054	0.710 ± 0.047	0.792 ± 0.059
RDKit	DNN	0.817 ± 0.063	0.671 ± 0.089	0.782 ± 0.070
	KNN	0.831 ± 0.053	0.736 ± 0.065	0.778 ± 0.068
	NB	0.753 ± 0.068	0.605 ± 0.083	0.672 ± 0.108
	RF	0.840 ± 0.073	0.725 ± 0.073	0.835 ± 0.067
	SVM	0.805 ± 0.091	0.656 ± 0.086	0.761 ± 0.077
	XGBoost	0.836 ± 0.084	0.740 ± 0.071	0.839 ± 0.060
	Mean	0.814 ± 0.032	0.689 ± 0.054	0.778 ± 0.061

a DNN: Deep neural networks.

b KNN: K-Nearest Neighbor.

c NB: Naïve Bayesian.

d RF: Random forest.

e SVM: Support vector machine.

f XGBoost: Extreme gradient boosting.

g GCN: Graph convolutional networks.

^h^GAT: Graph attention network.

i MPNN: Message passing neural networks.

j F1 scores: F1-measure.

k BA: Balanced accuracy.

l AUC: Area under the receiver operating characteristics curve.

### 3.4 Performance of Graph-Based Prediction Models for Breast-Associated Cells

**FIGURE 6:**
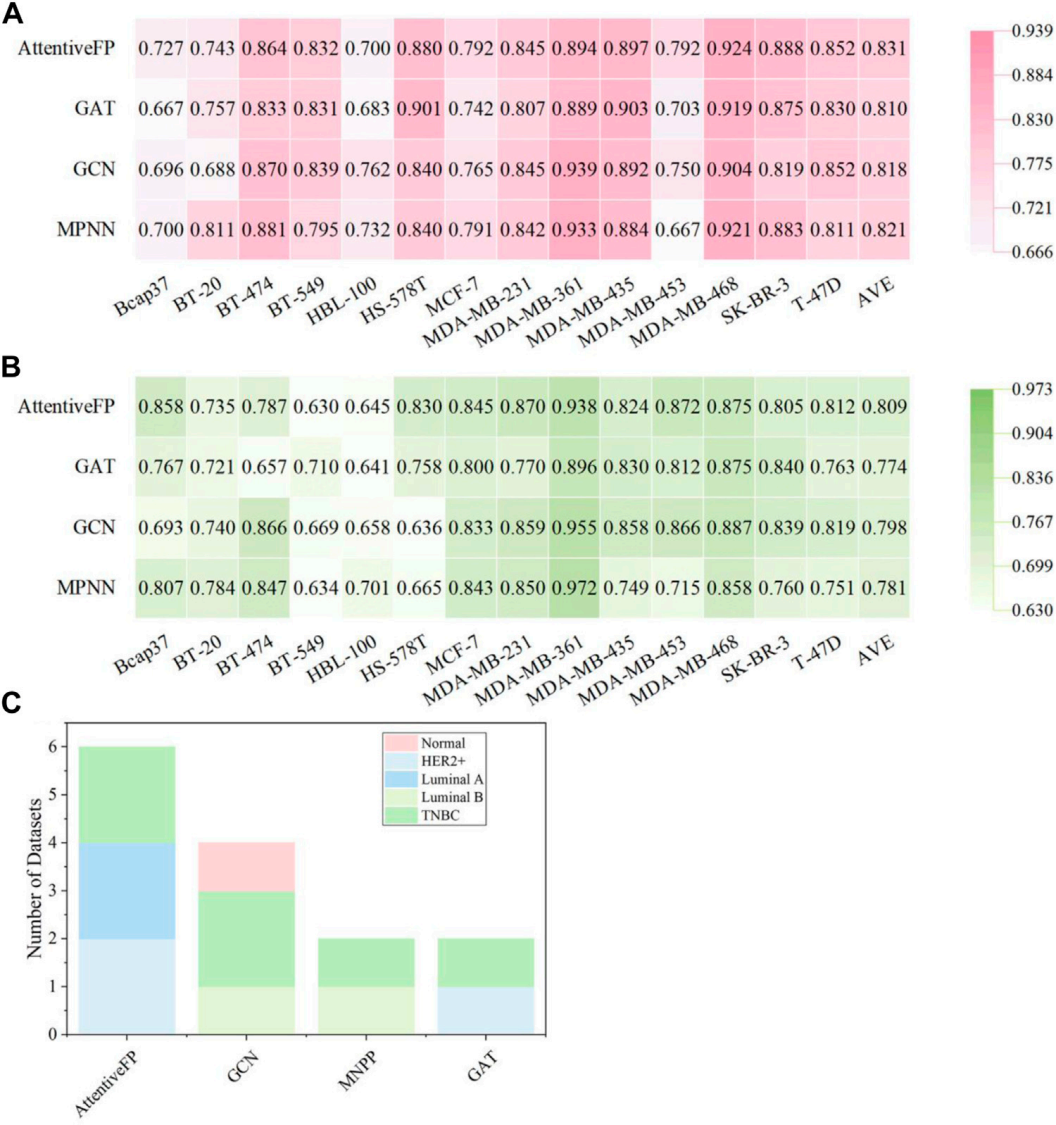
Performance of graph-based BC prediction models. **(A)** F1 scores of graph-based models. **(B)** AUC results of graph-based models. **(C)** The optimal models based on molecular graph for different subtypes of breast cell lines.

Compared with the traditional pre-tailored molecular descriptors and/or fingerprints, the key feature of GNN is its capacity to automatically learn task-specific molecular representations using graph convolutions. The SOAT accuracies of GNN models and their variants (e.g., GCN, MPNN, GAT, and Attentive FP) have been reported in various molecular property prediction tasks (Wu et al., 2018; Yang et al., 2019; Xiong et al., 2020). Therefore, 56 molecular graph-based models were established using four types of DL algorithms, including GCN, MPNN, GAT, and Attentive FP. The detailed performance results of molecular graph-based models are listed in Supplementary Table S7. As shown in Figure 6, the Attentive FP models exhibited the overall best performance compared with other GNN methods, with a relatively higher average F1 score (0.831 ± 0.070) and AUC (0.809 ± 0.086). The BA results are shown in Supplementary Figure S4. Figure 6C shows that the Attentive FP models performed the best in six breast cancer cell lines including Bcap37, MCF-7, MDA-MB-453, MDA-MB-468, SK-BR-3, and T-47D, making it the most frequent choice. The GCN models showed the best performance in four breast cell lines (BT-549, HBL-100, MDA-MB-231, and MDA-MB-361), the MPNN models performed the best in BT-20 and BT-474 cell lines, and the GAT models performed the best in HS-578T and MDA-MB-435 cell lines.

One advantage of the DL model is its capacity for multi-task model building for attribute-related datasets to improve the accuracy of the single-task model (Li et al., 2018). Therefore, the multi-task models were trained by the entire 13 breast cancer cell-compound datasets based on the features of the Morgan fingerprints using DNN and molecular graphs using GCN, Attentive FP. Supplementary Table S8 shows that the AUC of the multi-task models was not better than that of the single-task models. Further data point distribution analysis found that the number of common compounds shared by 13 cell line datasets was small (only 12 molecules, Supplementary Figure S5), which explains the poor performance results (Supplementary Table S8) of the multi-task models.

### 3.5 The Optimal Model for Each Breast Cell Line and Further Validation

Comparison of the established molecular descriptor-, fingerprint-, and graph-based models showed that Eq. 1 the RF algorithm had a better performance capability than the other five ML methods, with higher average metric values of F1 score, BA, and AUC (Table 2) in both descriptor- and fingerprint-based models, while XGBoost also achieved comparable results for these 14 modeling datasets (Table 2 and Figure 5A); 2) among the established 56 graph-based models, Attentive FP architecture outperformed the other three deep graph learning approaches (i.e., GCN, MPNN, and GAT) on average across all 14 datasets (Table 2); and 3) the performance of molecular fingerprint-based models is generally better than that of both descriptor- and graph-based models at least in these 14 datasets (Table 2), implying that graph DL methods do not achieve better results than the traditional ML learning methods (especially for the two most efficient algorithms, XGBoost and RF), which is consistent with a recent systematic comparison study (Jiang D. et al., 2021).

According to the metrics of F1 score, BA, and AUC from the test sets, the optimal in silico predictive model for each breast cell line is listed in Supplementary Table S9. Fingerprint-based RF models performed the best because they ranked first in eight of 14 cell lines. Fingerprint-based XGBoost and SVM models are tied for second place and performed best in two of 14 breast cell lines each. For example, the RF:Morgan model achieved higher prediction results against MDA-MB-231 and T-47D breast cancer cell lines, with ACC values of 83.7 and 84.0%, respectively, and AUC values of 0.904 and 0.885, respectively. The lack of selectivity for cancer cells rather than normal cells is one of the main factors that limit the development of anticancer drugs for clinical use (Dy and Adjei, 2013; Guo et al., 2020). For one normal breast cell line (HBL-100), the RF:Morgan model also showed good prediction results, with ACC and AUC values of 83.9%, and 0.823, respectively, suggesting that this model can be used to detect whether a given molecule selectively inhibits breast cancer cells over normal human breast cells.

Model fusion may improve the classification prediction performance of a single model by combining the classification prediction results from the corresponding multiple models. Both voting and stacking methods were used in this study for model fusion. As shown in Table 2, Morgan fingerprint-based models performed the best in different kinds of fingerprint-based models with an average F1 score of 0.827 ± 0.026, and RF, XGBoost, and SVM algorithms performed best in most of the datasets (Figures 5A,E). Therefore, RF, SVM, and XGBoost models for model fusion were applied based on Morgan fingerprints. A total of 112 fusion models were established, and detailed performance results for these voting and stacking models are listed in Supplementary Tables S10, S11. As shown in Supplementary Figure S6, the average F1 scores of voting or stacking models were similar in each dataset. In all the datasets of breast cell lines, the RF + XGBoost voting model showed the best average performance among fusion models, with average F1, BA, and AUC of 0.849 ± 0.066, 0.749 ± 0.075, and 0.845 ± 0.075, respectively. The fusion models based on Morgan fingerprints were slightly but not significantly better than the single models.

**FIGURE 7:**
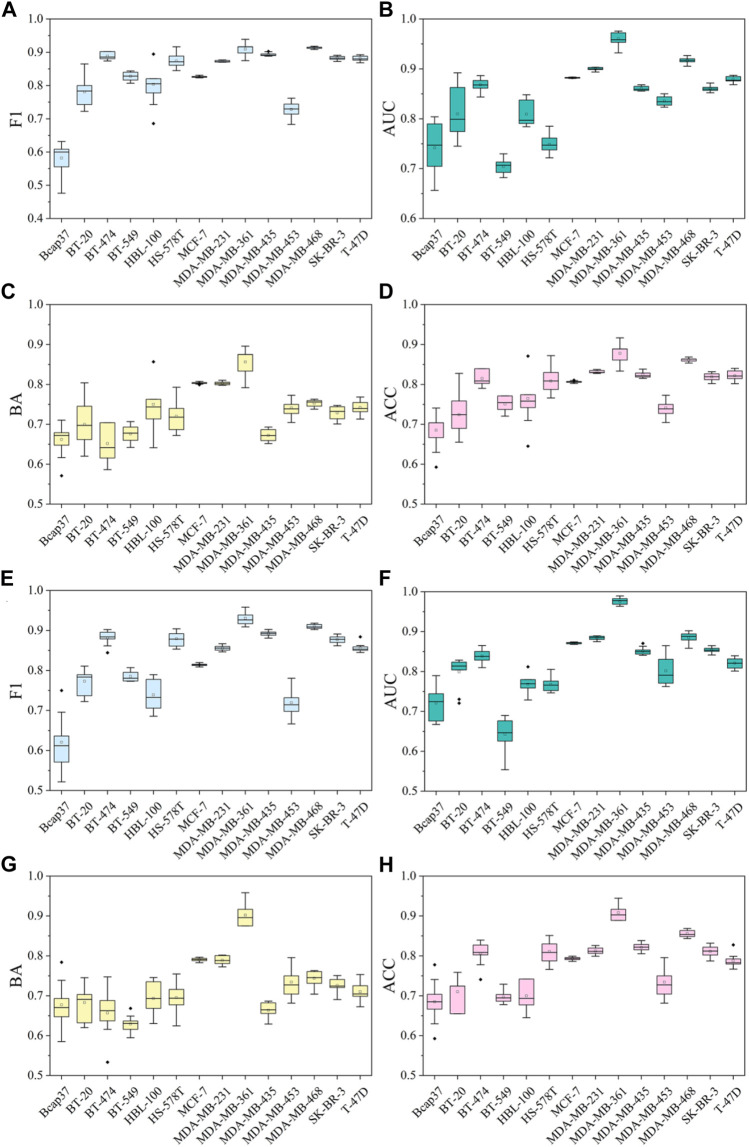
The performance of 10-fold cross-validation results in RF:Morgan and XGBoost:Morgan models. **(A–D)** F1 scores, AUC, BA, and ACC results in RF:Morgan models. **(E–H)** F1 scores, AUC, BA, and ACC results in XGBoost:Morgan models.

To validate the stability and reliability of the models presented, 10-fold cross-validation and 10 different random seeds of data were used to retrain the models based on the combination of Morgan fingerprints and two ML algorithms (RF and XGBoost). The performance of 10-fold cross-validation classification models is summarized in Supplementary Table S12 and Figure 7. Overall, all RF:Morgan models performed well, showing high F1 scores of 0.582–0.914, AUC values of 0.704–0.960, and ACC values of 0.685–0.878. XGBoost:Morgan models showed a similar trend in the 10-fold cross-validation experiment. In 14 cell line datasets, both RF:Morgan and XGBoost:Morgan models consistently exhibited better performance with different seeds (Supplementary Figure S7), and the performance showed comparable or smaller variation compared with the previous models based on a specific random seed. Taken together, these results demonstrate that the models presented in this study show stability and reliability. Y-scrambling testing was used to demonstrate that the results are not attributed to chance correlation. As illustrated in Supplementary Figure S8, S9, the F1 scores, BA, and AUC values of the RF:Morgan and XGBoost:Morgan models were significantly higher than those of any of the Y-scrambled models, which confirmed that the results were not chance correlations.

### 3.6 Interpretation of the Optimal Model for Each Breast Cell Line

**FIGURE 8:**
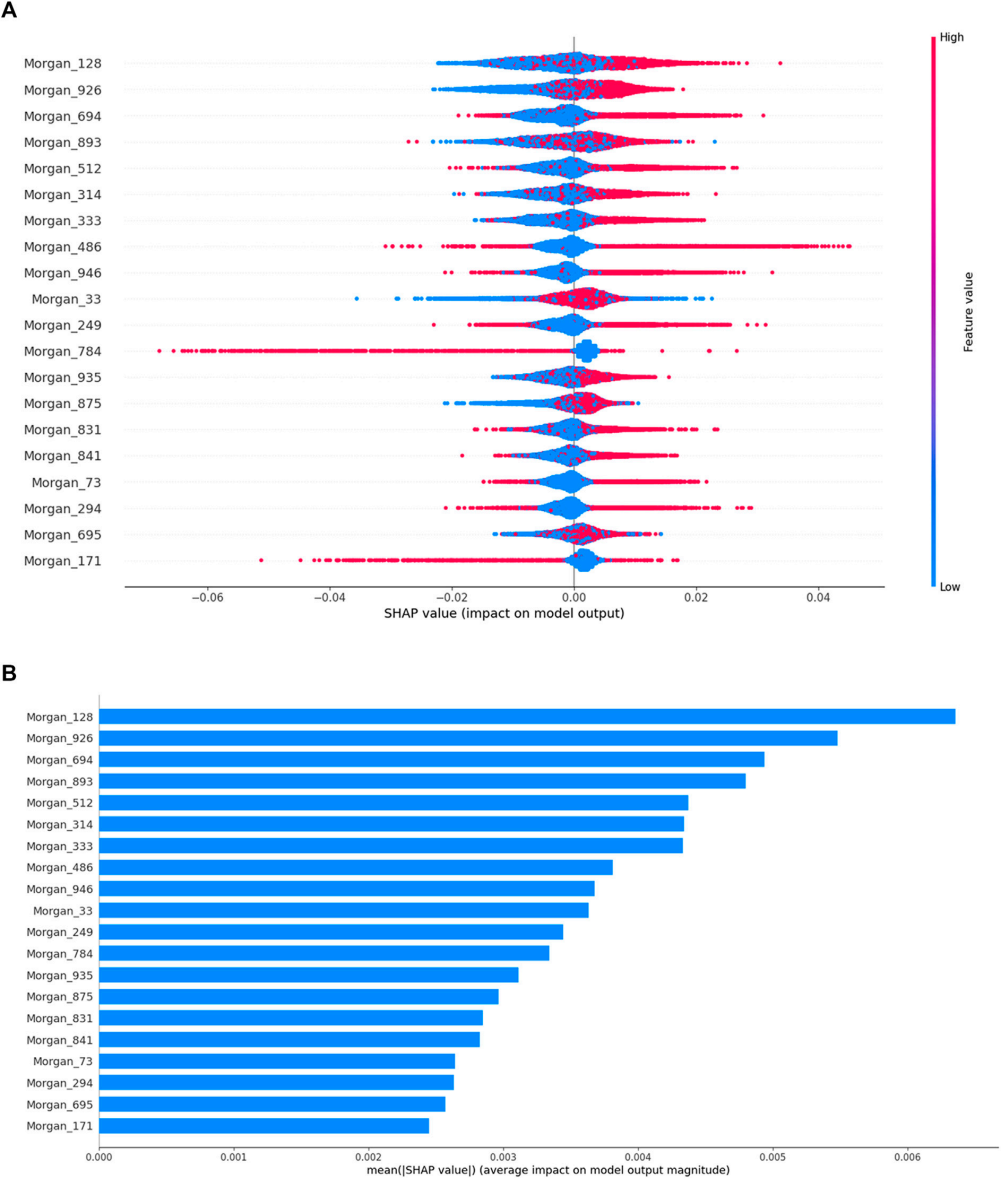
Based on the top 20 most important features of the RF:Morgan model in MDA-MB-231, **(A)** the SHAP values for each molecular substructure, and **(B)** the mean of the absolute value of the SHAP value for each molecular substructure.

**FIGURE 9:**
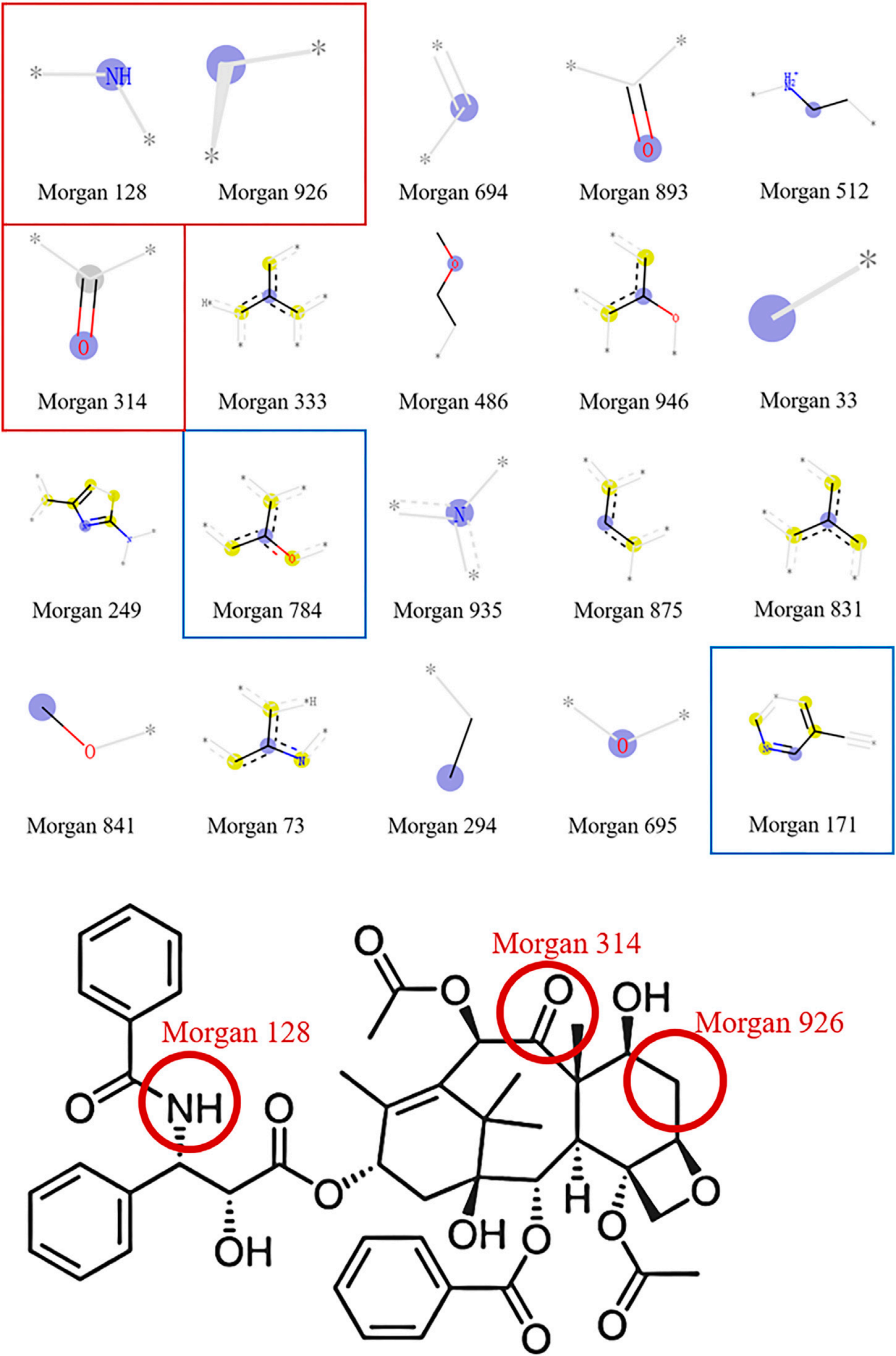
Important molecular substructures of the RF:Morgan model in MDA-MB-231 and the chemical structural of paclitaxel.

To gain a deeper understanding of the established models, we used the SHAP method to calculate the contribution of important structural fragments. Because models based on the combination of the RF and Morgan fingerprints had relatively high predictive performance, we used TreeExplainer, a tree explanation method in SHAP, to calculate the optimal local explanation for these RF:Morgan models. In the MDA-MB-231 cell line as an example, the top 20 favorable and unfavorable structural fragments for MDA-MB-231 inhibition were determined based on the SHAP value and are displayed in Figures 8, 9. As shown in Figure 8A, the feature values are represented by different colors (red to blue). Redder points indicate larger feature values. Morgan fingerprints only contain 1 (with this structural fragment, red) and 0 (without this structural fragment, blue). For Morgan 128, Morgan 926, and Morgan 314 in Figure 8A, most of the red points are in the positive value part and most of the blue points are in the negative value part, indicating that the predicted molecules with these fragments will have a higher probability of anti-BC activity. On the contrary, Morgan 784 and Morgan 171 have more red points in the negative value part, indicating that high probabilities are judged by the model as having no inhibitory effect on the MDA-MB-231 cell line. Taking paclitaxel (a typical drug for BC treatment) as an example, it contains Morgan 128, Morgan 926, and Morgan 314 but does not contain Morgan 784 and Morgan 171, implying that it will be predicted to have an inhibitory effect on the MDA-MB-231 cell line. In fact, this is consistent with actual predictions and experimental results. The top 20 important structural fragments for other breast cell lines are shown in Supplementary Figure S10–S35, which may facilitate anti-BC lead compound selection and optimization.

### 3.7 Model AD

To further evaluate the generalization capability of our models, the LOF algorithm was applied to detect super-applicability domain compounds in the datasets. We first reduced the Morgan fingerprints of 1,024 bits to two dimensions by Principal Component Analysis in Scikit-learn and then used the LOF module for calculation. As shown in Supplementary Figure S36, there are fewer red points, which indicates that each dataset has fewer super-applicability domain compounds. Therefore, selecting compounds that are similar to those in the datasets of this study may result in higher prediction accuracy when using the present model. The molecular (feature) spaces can be used to define the applicability domain, thus, a simpler way to determine whether a molecule fits the models of this study is to directly calculate the molecular weight of the molecule. Since the molecular weight range of the molecules in this study is 108.10–5,714.45, we recommend using molecules in this range for prediction, which can make the prediction more accurate.

### 3.8 Webserver and Local Version Software for the Prediction of Anti-BC Agents

**FIGURE 10:**
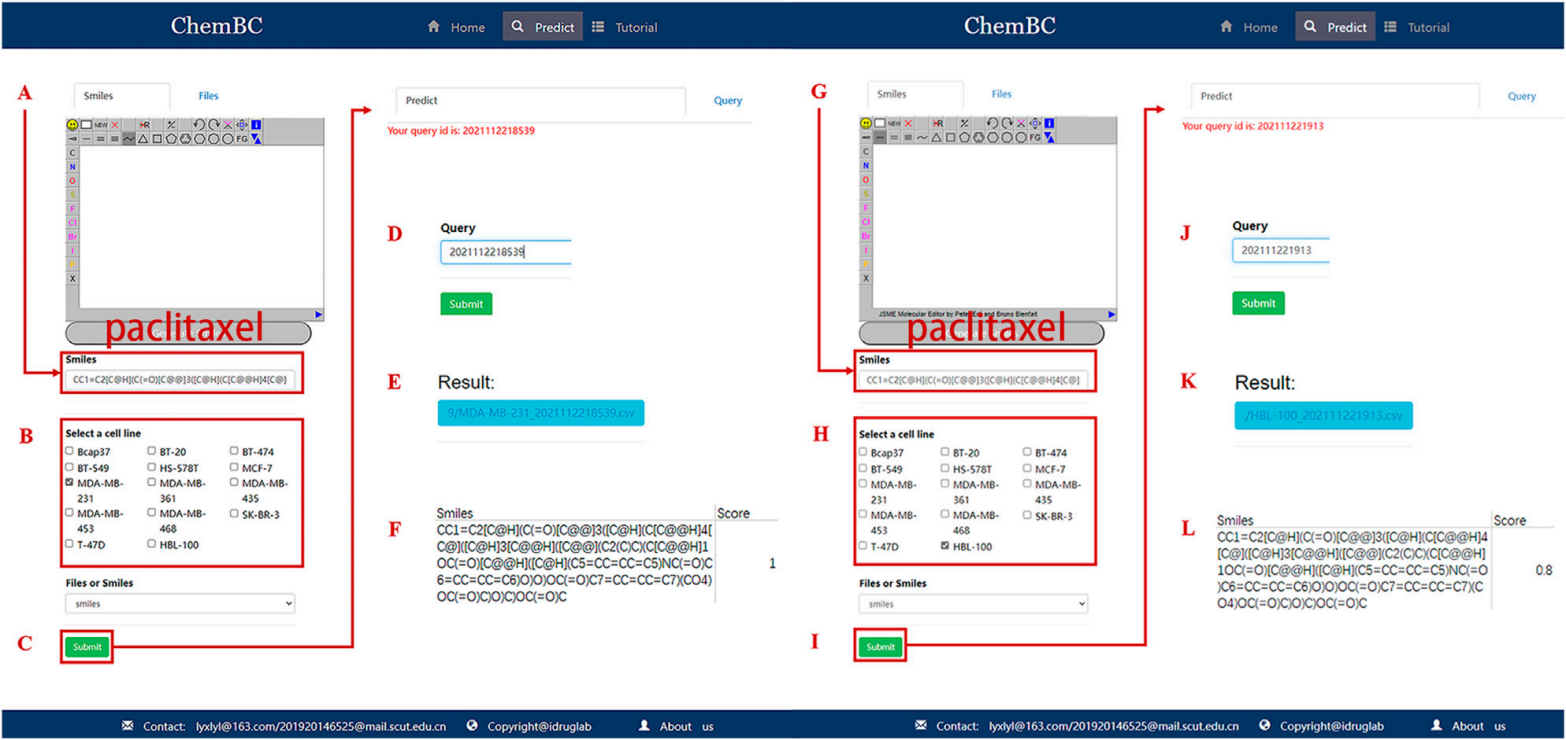
Website schematic diagram of bioactivity prediction. **(A–F)** represents prediction of paclitaxel inhibition for MDA-MB-231 cell line. **(G–L)** represents prediction of paclitaxel inhibition against HBL-100 cell line.

To facilitate the use of these models by experts and non-experts in the field, we built a web-based online forecasting system called ChemBC (http://chembc.idruglab.cn/). To expand the AD threshold of the established model, we retained models for each breast cell line according to the combination of Morgan fingerprint and RF using the entire dataset, and then implemented these retained models into ChemBC and its local version. According to the 10-fold cross-validation (AUC = 0.780–0.928, ACC = 0.714–0.880), the retrained models for 14 breast cell line datasets showed excellent predictive performance. ChemBC was developed based on the Django framework using the Python package. The main functional module of ChemBC is prediction (Figure 10) in which users can upload and/or online draw a structure to easily and quickly predict the inhibitory activity against 13 breast cancer cell lines and one normal breast cell line. In addition, a local version executable software (https://github.com/idruglab/ChemBC) was developed to perform large-scale VS screening.

Taking paclitaxel as an example, it has a predicted score of 1.0 in the MDA-MB-231 model, proving that it has a strong inhibitory effect on the MDA-MB-231 cell line. Meanwhile, it has a predicted score of 0.8 in the normal breast cell line (HBL-100), suggesting that it is also toxic to the normal breast cell. Therefore, the ChemBC webserver can not only predict whether the compound has an inhibitory effect on breast cancer cells but also predict whether the compound is toxic to one normal breast cell.

## 4 Conclusion

In this study, we collected datasets of phenotypic compound-cell association bioactivity toward 13 breast cancer cell lines and one normal breast cell line and constructed 588 models based on three molecular representatives, including molecular descriptors, fingerprints, and graphs using five conventional ML and five DL algorithms. Compared with these established models, the performance of RF:Morgan models was superior to that of the other models based on molecular descriptors and graphs. Based on RF:Morgan models, the important favorable and unfavorable fragments for each breast cell line generated using SHAP algorithms will be helpful for lead optimization or the design of new agents with better anti-BC activity. Although some fusion models based on voting and stacking methods showed better performance than single models, the observed improvement was minor. Finally, the online platform ChemBC and its local version software were developed based on well-established models, which could contribute to research aimed at designing and discovering new anti-BC agents. With the growth of compound toxicity data for BC and normal breast cell lines, we will add more prediction models in future studies.

## Data Availability

The original contributions presented in the study are included in the article/Supplementary Material, further inquiries can be directed to the corresponding authors.
