# Corrigendum: Machine Learning Enables Accurate and Rapid Prediction of Active Molecules Against Breast Cancer Cells

**DOI:** 10.3389/fphar.2022.901513

**Published:** 2022-05-30

**Authors:** Shuyun He, Duancheng Zhao, Yanle Ling, Hanxuan Cai, Yike Cai, Jiquan Zhang, Ling Wang

**Affiliations:** ^1^ Guangdong Provincial Key Laboratory of Fermentation and Enzyme Engineering, Guangdong Provincial Engineering and Technology Research Center of Biopharmaceuticals, School of Biology and Biological Engineering, South China University of Technology, Guangzhou, China; ^2^ Joint International Research Laboratory of Synthetic Biology and Medicine, Guangdong Provincial Engineering and Technology Research Center of Biopharmaceuticals, School of Biology and Biological Engineering, South China University of Technology, Guangzhou, China; ^3^ Center for Certification and Evaluation, Guangdong Drug Administration, Guangzhou, China; ^4^ State Key Laboratory of Functions and Applications of Medicinal Plants, College of Pharmacy, Guizhou Provincial Engineering Technology Research Center for Chemical Drug R&D, Guizhou Medical University, Guiyang, China

**Keywords:** breast cancer, machine learning, graph neural networks, molecular fingerprints, structural fragments

In the original article, there was a mistake in Figures 4 and 5 as published. There are some errors in the figure insertion, Figure 4 is repeated with **Figure 3**, and Figure 5 is the result of Figure 4. The corrected Figures 4 and 5 appear below.

**FIGURE 4:**
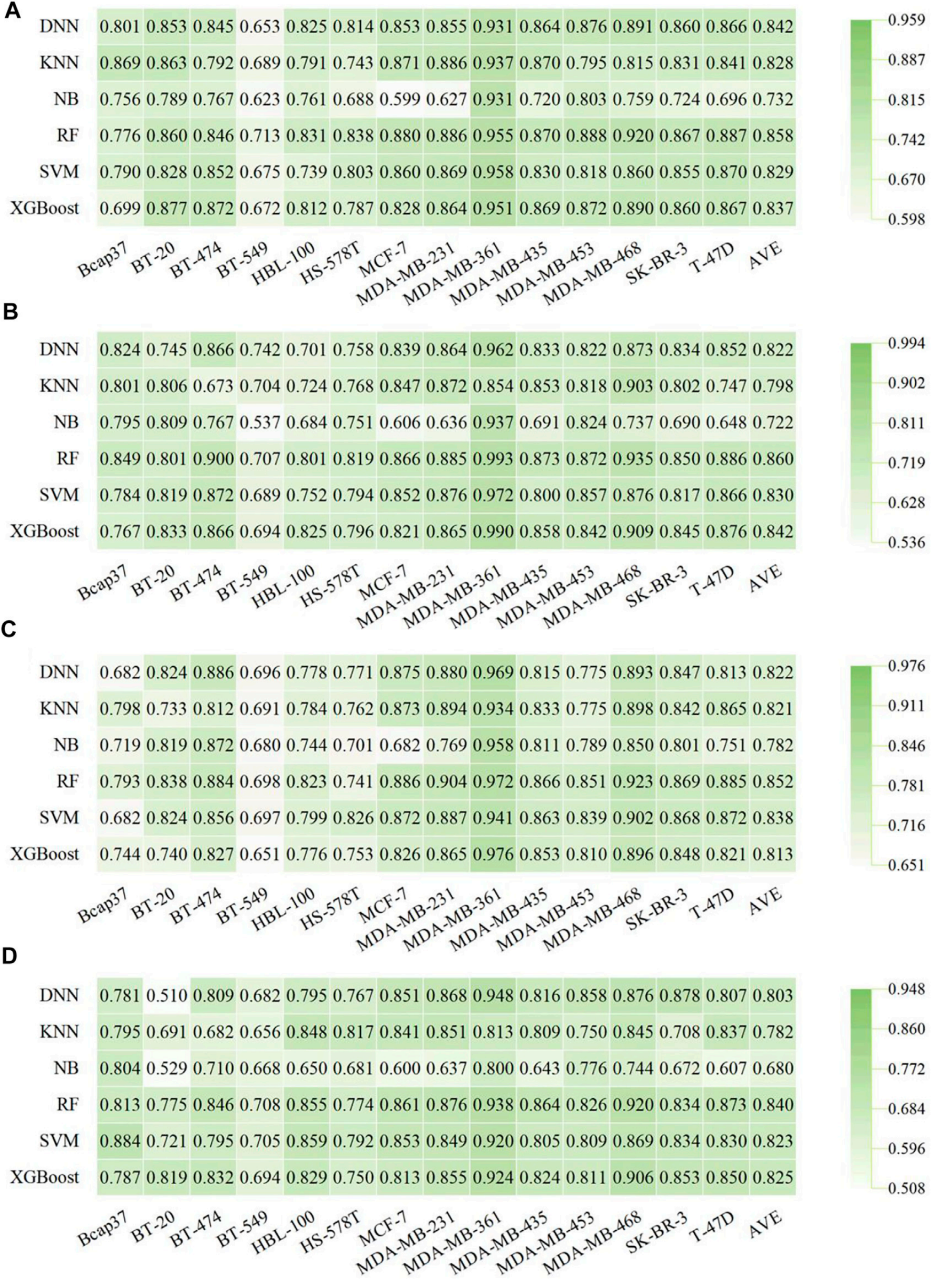
Performance of fingerprint-based BC prediction models. **(A)** AUC results of the AtomPairs-based models. **(B)** AUC results of the MACCS-based models. **(C)** AUC results of the Morgan-based models. **(D)** AUC results of the PharmacoPFP-based models.

**FIGURE 5:**
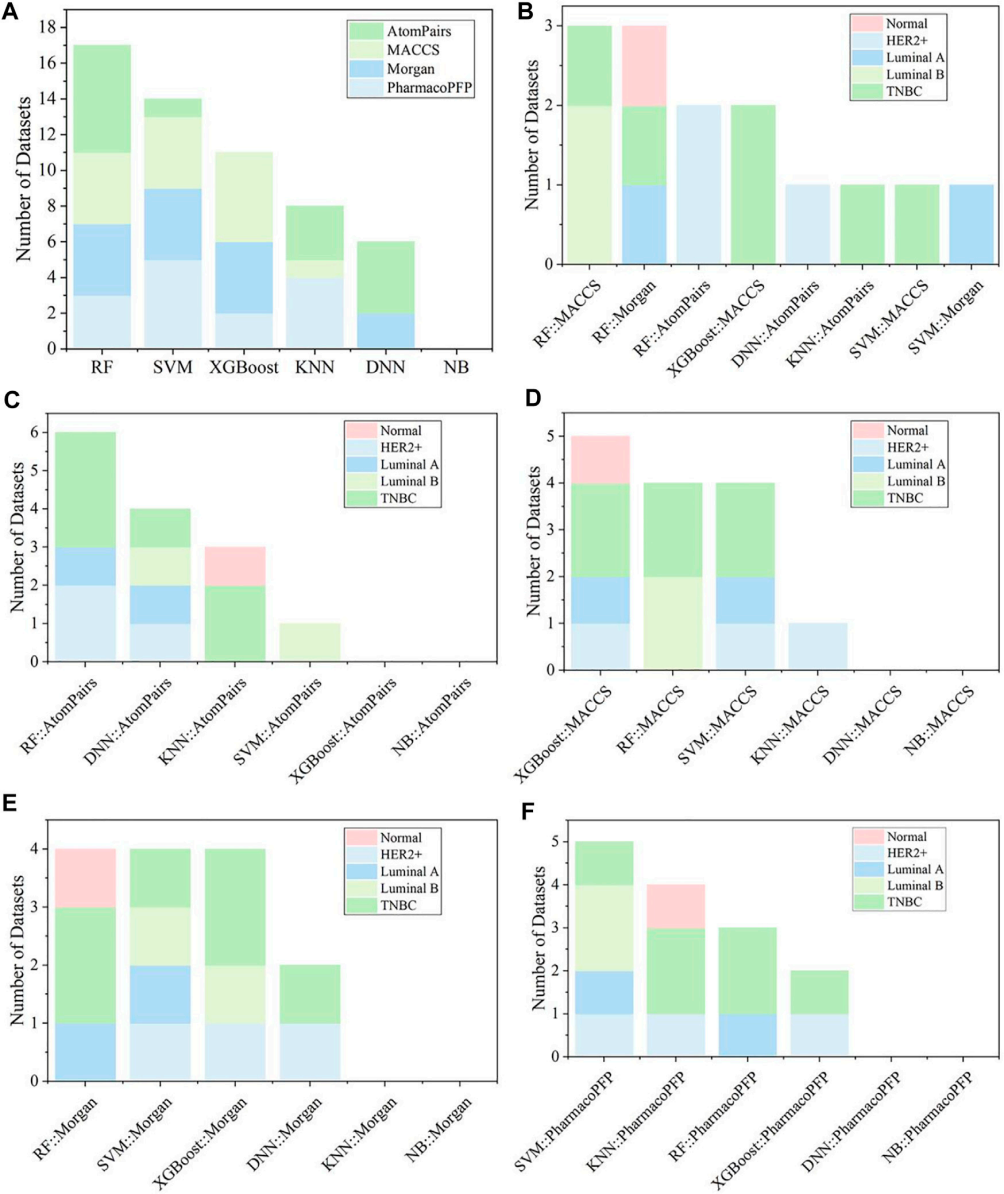
**(A)** Summary of the optimal models for each fingerprint-based feature. **(B)** The best models among various fingerprint-based models for different kinds of breast cell lines. The optimal models based on **(C)** AtomPairs, **(D)** MACCS, **(E)** Morgan, and **(F)** PharmacoPFP for different subtypes of breast cell lines.

The authors apologize for this error and state that this does not change the scientific conclusions of the article in any way. The original article has been updated.

