# Supplementary material for: Machine Learning Enables Accurate and Rapid Prediction of Active Molecules Against Breast Cancer Cells
Source: Front Pharmacol. 2021 Dec 17;12:796534. doi: 10.3389/fphar.2021.796534 (PMC8719637; doi:10.3389/fphar.2021.796534)
Supplement: Supplementary file 1 [file DataSheet1.docx]

Supplementary Material

**Machine learning enables accurate and rapid prediction of active molecules against breast cancer cells**

Shuyun He,^1,‡^ Duancheng Zhao,^1,‡^ Yanle Ling^1^, Hanxuan Cai^1^, Yike Cai^3^, Jiquan Zhang^2,*^ and Ling Wang^1,*^

^1^Guangdong Provincial Key Laboratory of Fermentation and Enzyme Engineering, Joint International Research Laboratory of Synthetic Biology and Medicine, Guangdong Provincial Engineering and Technology Research Center of Biopharmaceuticals, School of Biology and Biological Engineering, South China University of Technology, Guangzhou 510006, China.

^2^State Key Laboratory of Functions and Applications of Medicinal Plants & College of Pharmacy, Guizhou Provincial Engineering Technology Research Center for Chemical Drug R&D, Guizhou Medical University, Guiyang, 550004, China.

^3^Center for Certification and Evaluation, Guangdong Drug Administration, Guangzhou 510080, China.

^‡^These authors contributed equally to this work

*To whom correspondence should be addressed.

**Contents**

**Supplementary Figure S1.** Analysis of 14 datasets. (A) Number of compounds in all datasets. (B) Percentage of active and inactive compounds in all breast cell lines.

**Supplementary Figure S2.** The chemical space of the compounds in (A) Bcap37, (B) BT-20, (C) BT-474, (D) BT-549, (E) HS-578T, (F) MCF-7, (G) MDA-MB-231, (H) MDA-MB-361, (I) MDA-MB-435, (J) MDA-MB-453, (K) MDA-MB-468, (L) SK-BR-3, (M) T-47D, (N) HBL-100 datasets.

**Supplementary Figure S3.** Performance of fingerprint‑based BC prediction models. (A) BA results of the AtomPairs-based models. (B) BA results of the MACCS-based models. (C) BA results of the Morgan-based models. (D) BA results of the PharmacoPFP-based models.

**Supplementary Figure S4.** BA results of graph‑based BC prediction models.

**Supplementary Figure S5.** Common molecules in 13 breast cancer cell lines.

**Supplementary Figure S6.** F1 scores of fusion models.

**Supplementary Figure S7.** The performance of 10 random seeds in RF and XGBoost::Morgan models. (A-D) F1scores, AUC, BA, and ACC results in RF::Morgan models; (E-H) F1scores, AUC, BA, and ACC results in XGBoost::Morgan models.

**Supplementary Figure S8.** Y-scrambling results for the RF::Morgan models. Both the training sets and testing sets were unscrambled (gold). The training sets were scrambled, whereas the test sets were unscrambled (green). The training sets were unscrambled, whereas the test sets were scrambled (purple). F1: F1 scores. BA: Balanced accuracy. AUC: Area under the receiver operating characteristics curve.

**Supplementary Figure S9.** Y-scrambling results for the XGB::Morgan models. Both the training sets and testing sets were unscrambled (gold). The training sets were scrambled, whereas the test sets were unscrambled (green). The training sets were unscrambled, whereas the test sets were scrambled (purple). F1: F1 scores. BA: Balanced accuracy. AUC: Area under the receiver operating characteristics curve.

**Supplementary Figure S10.** Based on the top 20 most important features of the RF::Morgan model in Bcap37, (A) the SHAP values for each molecular substructure, and (B) the mean of the absolute value of the SHAP value for each molecular substructure.

**Supplementary Figure S11.** Based on the top 20 most important features of the RF::Morgan model in BT-20, (A) the SHAP values for each molecular substructure, and (B) the mean of the absolute value of the SHAP value for each molecular substructure.

**Supplementary Figure S12.** Based on the top 20 most important features of the RF::Morgan model in BT-474, (A) the SHAP values for each molecular substructure, and (B) the mean of the absolute value of the SHAP value for each molecular substructure.

**Supplementary Figure S13.** Based on the top 20 most important features of the RF::Morgan model in BT-549, (A) the SHAP values for each molecular substructure, and (B) the mean of the absolute value of the SHAP value for each molecular substructure.

**Supplementary Figure S14.** Based on the top 20 most important features of the RF::Morgan model in HBL-100, (A) the SHAP values for each molecular substructure, and (B) the mean of the absolute value of the SHAP value for each molecular substructure.

**Supplementary Figure S15.** Based on the top 20 most important features of the RF::Morgan model in HS-578T (A) the SHAP values for each molecular substructure, and (B) the mean of the absolute value of the SHAP value for each molecular substructure.

**Supplementary Figure S16.** Based on the top 20 most important features of the RF::Morgan model in MCF-7 (A) the SHAP values for each molecular substructure, and (B) the mean of the absolute value of the SHAP value for each molecular substructure.

**Supplementary Figure S17.** Based on the top 20 most important features of the RF::Morgan model in MDA-MB-361 (A) the SHAP values for each molecular substructure, and (B) the mean of the absolute value of the SHAP value for each molecular substructure.

**Supplementary Figure S18.** Based on the top 20 most important features of the RF::Morgan model in MDA-MB-435 (A) the SHAP values for each molecular substructure, and (B) the mean of the absolute value of the SHAP value for each molecular substructure.

**Supplementary Figure S19.** Based on the top 20 most important features of the RF::Morgan model in MDA-MB-453 (A) the SHAP values for each molecular substructure, and (B) the mean of the absolute value of the SHAP value for each molecular substructure.

**Supplementary Figure S20.** Based on the top 20 most important features of the RF::Morgan model in MDA-MB-468 (A) the SHAP values for each molecular substructure, and (B) the mean of the absolute value of the SHAP value for each molecular substructure.

**Supplementary Figure S21.** Based on the top 20 most important features of the RF::Morgan model in SK-BR-3 (A) the SHAP values for each molecular substructure, and (B) the mean of the absolute value of the SHAP value for each molecular substructure.

**Supplementary Figure S22.** Based on the top 20 most important features of the RF::Morgan model in T-47D (A) the SHAP values for each molecular substructure, and (B) the mean of the absolute value of the SHAP value for each molecular substructure.

**Supplementary Figure S23.** Important molecular substructures of the RF::Morgan model in Bcap37.

**Supplementary Figure S24.** Important molecular substructures of the RF::Morgan model in BT-20.

**Supplementary Figure S25.** Important molecular substructures of the RF::Morgan model in BT-474.

**Supplementary Figure S26.** Important molecular substructures of the RF::Morgan model in BT-549.

**Supplementary Figure S27.** Important molecular substructures of the RF::Morgan model in HBL-100.

**Supplementary Figure S28.** Important molecular substructures of the RF::Morgan model in HS-578T.

**Supplementary Figure S29.** Important molecular substructures of the RF::Morgan model in MCF-7.

**Supplementary Figure S30.** Important molecular substructures of the RF::Morgan model in MDA-MB-361.

**Supplementary Figure S31.** Important molecular substructures of the RF::Morgan model in MDA-MB-435.

**Supplementary Figure S32.** Important molecular substructures of the RF::Morgan model in MDA-MB-453.

**Supplementary Figure S33.** Important molecular substructures of the RF::Morgan model in MDA-MB-468.

**Supplementary Figure S34.** Important molecular substructures of the RF::Morgan model in SK-BR-3.

**Supplementary Figure S35.** Important molecular substructures of the RF::Morgan model in T-47D.

**Supplementary Figure S36.** Model AD in training sets and test sets in all breast cell lines. K was set to 5. By comparing the density of each point p and its five neighborhood points, whether this point is abnormal is judged. The lower the density of point p is, the more likely it is to be identified as an abnormal point. Exceptions are shown in red.

**Supplementary Table S1.** Summary of hyperparameter setting in different models. (Supplementary Table.xlsx)

**Supplementary Table S2.** The performance results of models based on RDKit descriptors. (Supplementary Table.xlsx)

**Supplementary Table S3.** The performance results of models based on AtomPairs fingerprints. (Supplementary Table.xlsx)

**Supplementary Table S4.** The performance results of models based on MACCS keys. (Supplementary Table.xlsx)

**Supplementary Table S5.** The performance results of models based on Morgan fingerprints. (Supplementary Table.xlsx)

**Supplementary Table S6.** The performance results of models based on PharmacoPFP. (Supplementary Table.xlsx)

**Supplementary Table S7.** The performance results of models based on molecular graph. (Supplementary Table.xlsx)

**Supplementary Table S8.** AUC results for Multi-task models. (Supplementary Table.xlsx)

**Supplementary Table S9.** The optimal *in silico* predictive model for each breast cell line. (Supplementary Table.xlsx)

**Supplementary Table S10.** The performance results of voting models based on Morgan fingerprints. (Supplementary Table.xlsx)

**Supplementary Table S11.** The performance results of stacking models based on Morgan fingerprints. (Supplementary Table.xlsx)

**Supplementary Table S12.** The performance results of 10-fold cross validation models based on Morgan fingerprints. (Supplementary Table.xlsx)


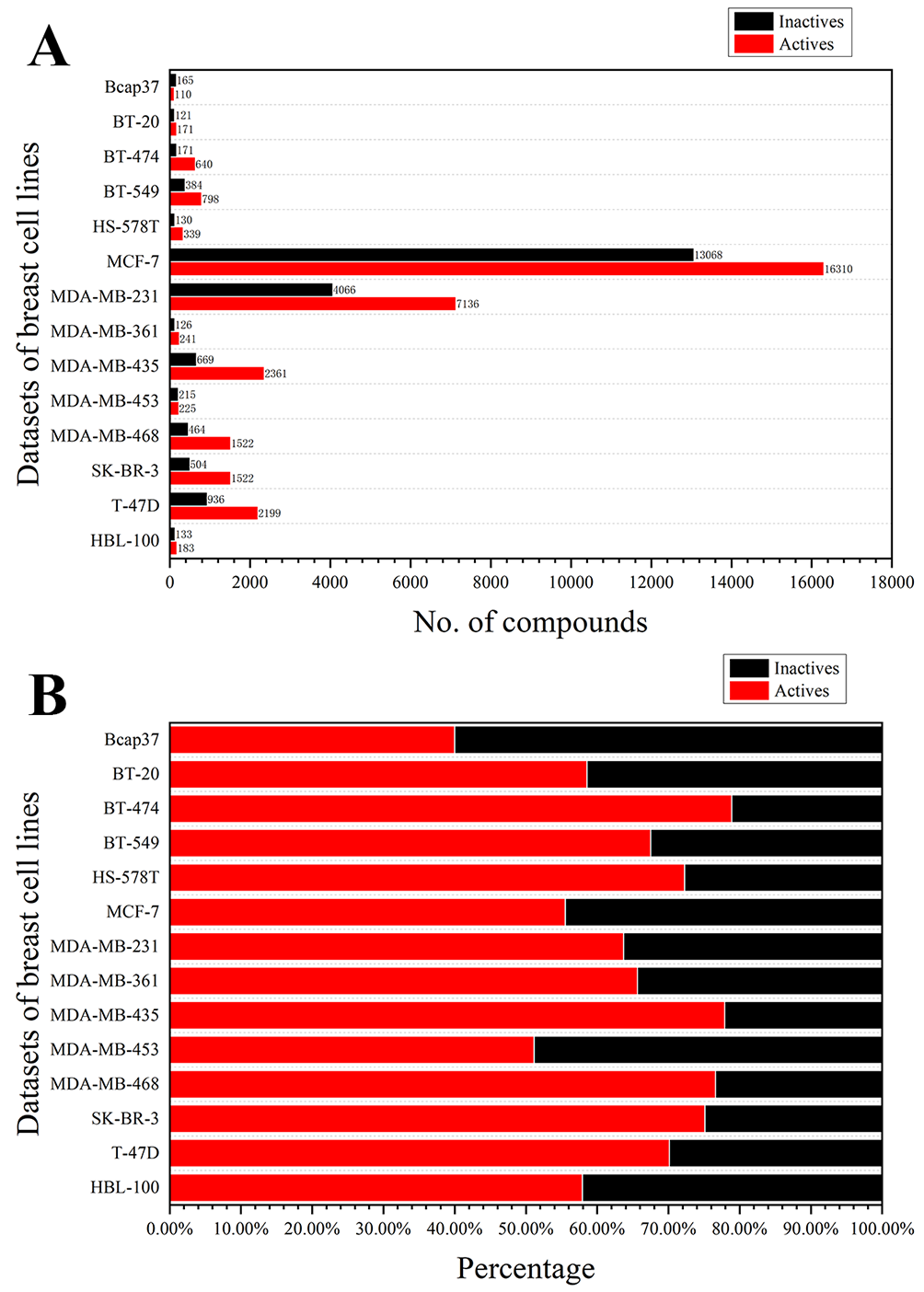


**Supplementary Figure S1.** Analysis of 14 datasets. (A) Number of compounds in all datasets. (B) Percentage of active and inactive compounds in all breast cell lines.


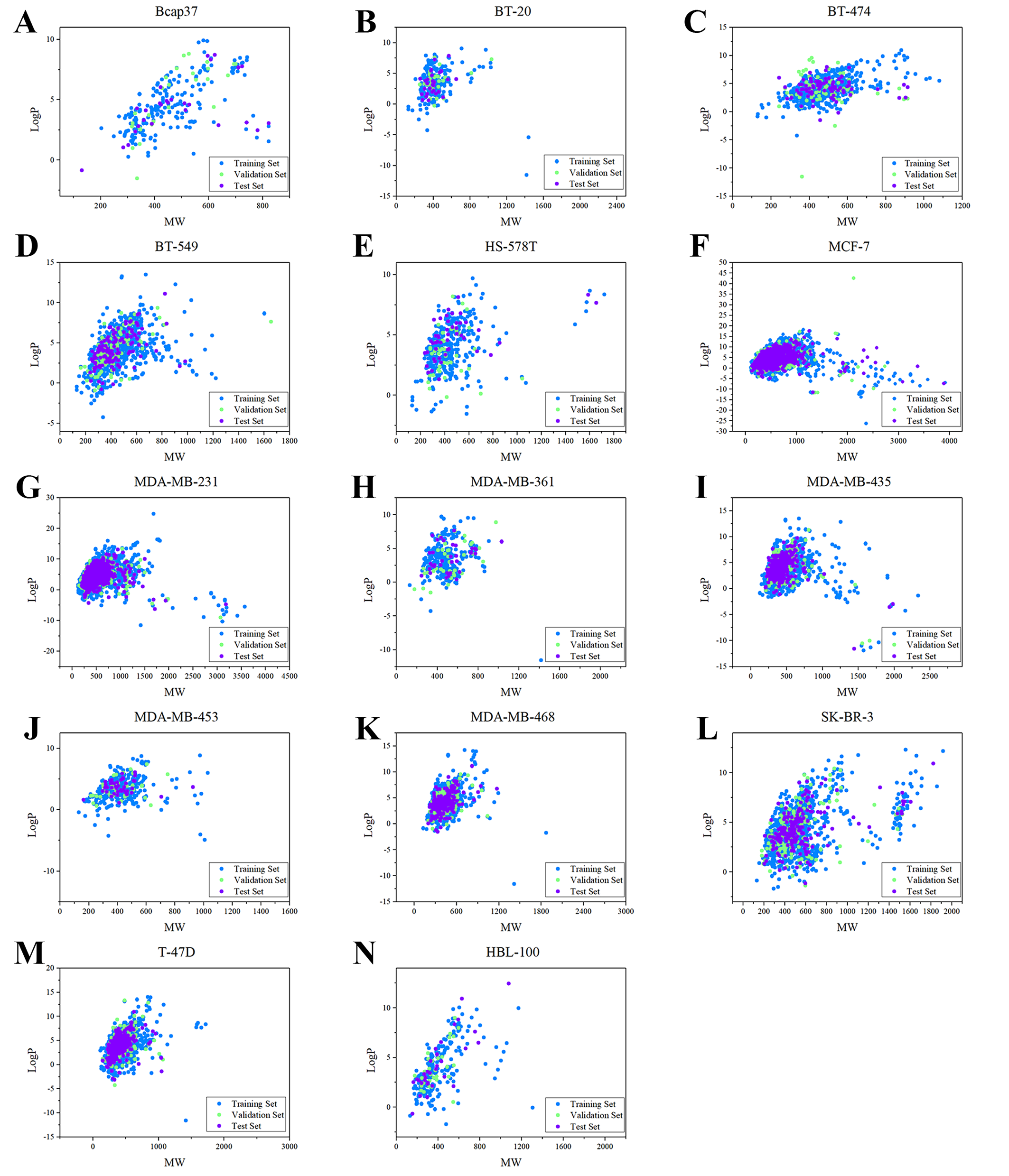


**Supplementary Figure S2.** The chemical space of the compounds in (A) Bcap37, (B) BT-20, (C) BT-474, (D) BT-549, (E) HS-578T, (F) MCF-7, (G) MDA-MB-231, (H) MDA-MB-361, (I) MDA-MB-435, (J) MDA-MB-453, (K) MDA-MB-468, (L) SK-BR-3, (M) T-47D, (N) HBL-100 datasets.

**

**

**Supplementary Figure S3.** Performance of fingerprint‑based BC prediction models. (A) BA results of the AtomPairs-based models. (B) BA results of the MACCS-based models. (C) BA results of the Morgan-based models. (D) BA results of the PharmacoPFP-based models.

**

**

**Supplementary Figure S4.** BA results of graph‑based BC prediction models.

**
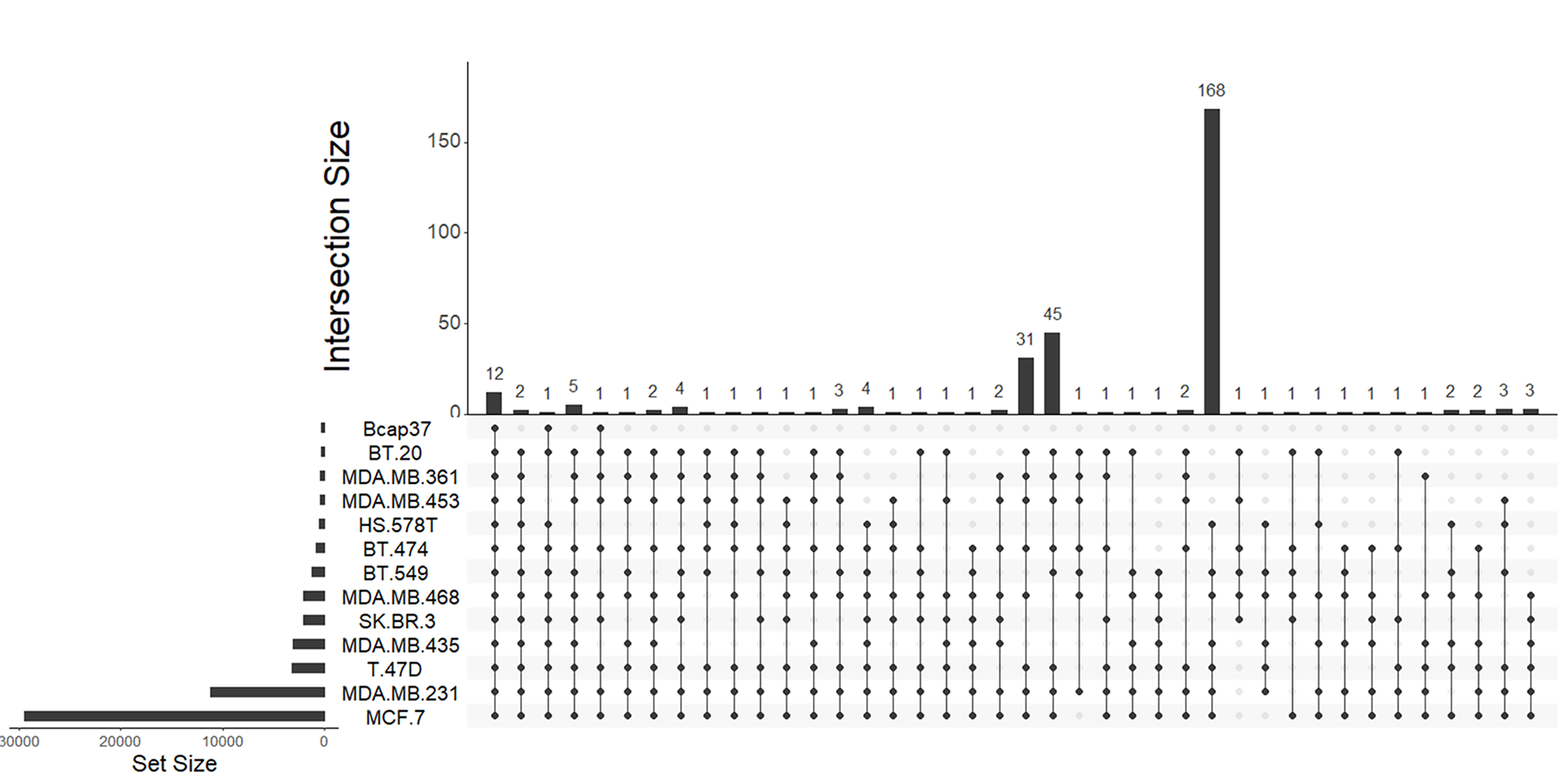
**

**Supplementary Figure S5.** Common molecules in 13 breast cancer cell lines.





**Supplementary Figure S6.** F1 scores of fusion models.


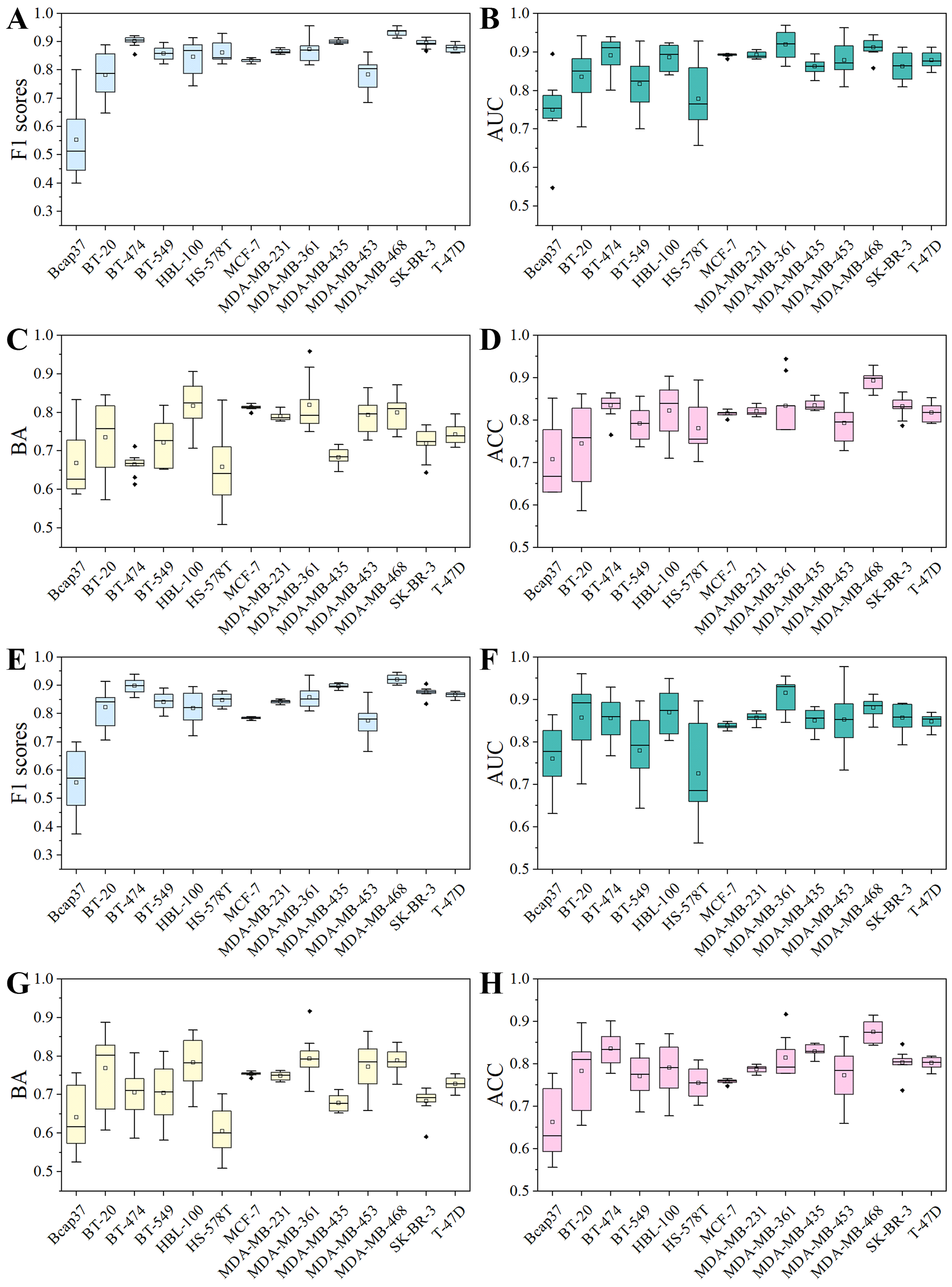


**Supplementary Figure S7.** The performance of 10 random seeds in RF and XGBoost::Morgan models. (A-D) F1scores, AUC, BA, and ACC results in RF::Morgan models; (E-H) F1scores, AUC, BA, and ACC results in XGBoost::Morgan models.

**
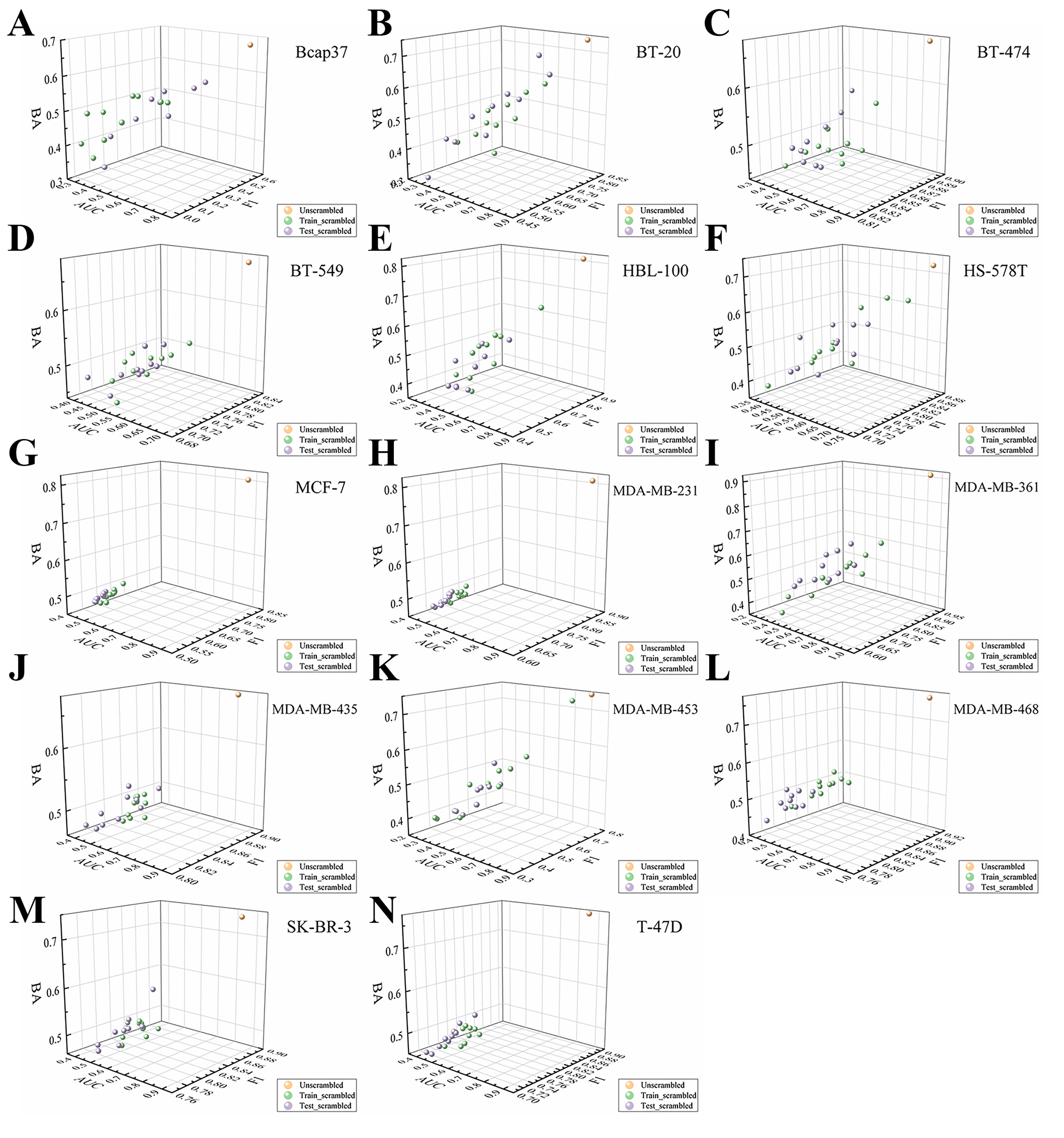
**

**Supplementary Figure S8.** Y-scrambling results for the RF::Morgan models. Both the training sets and testing sets were unscrambled (gold). The training sets were scrambled, whereas the test sets were unscrambled (green). The training sets were unscrambled, whereas the test sets were scrambled (purple). F1: F1 scores. BA: Balanced accuracy. AUC: Area under the receiver operating characteristics curve.

**
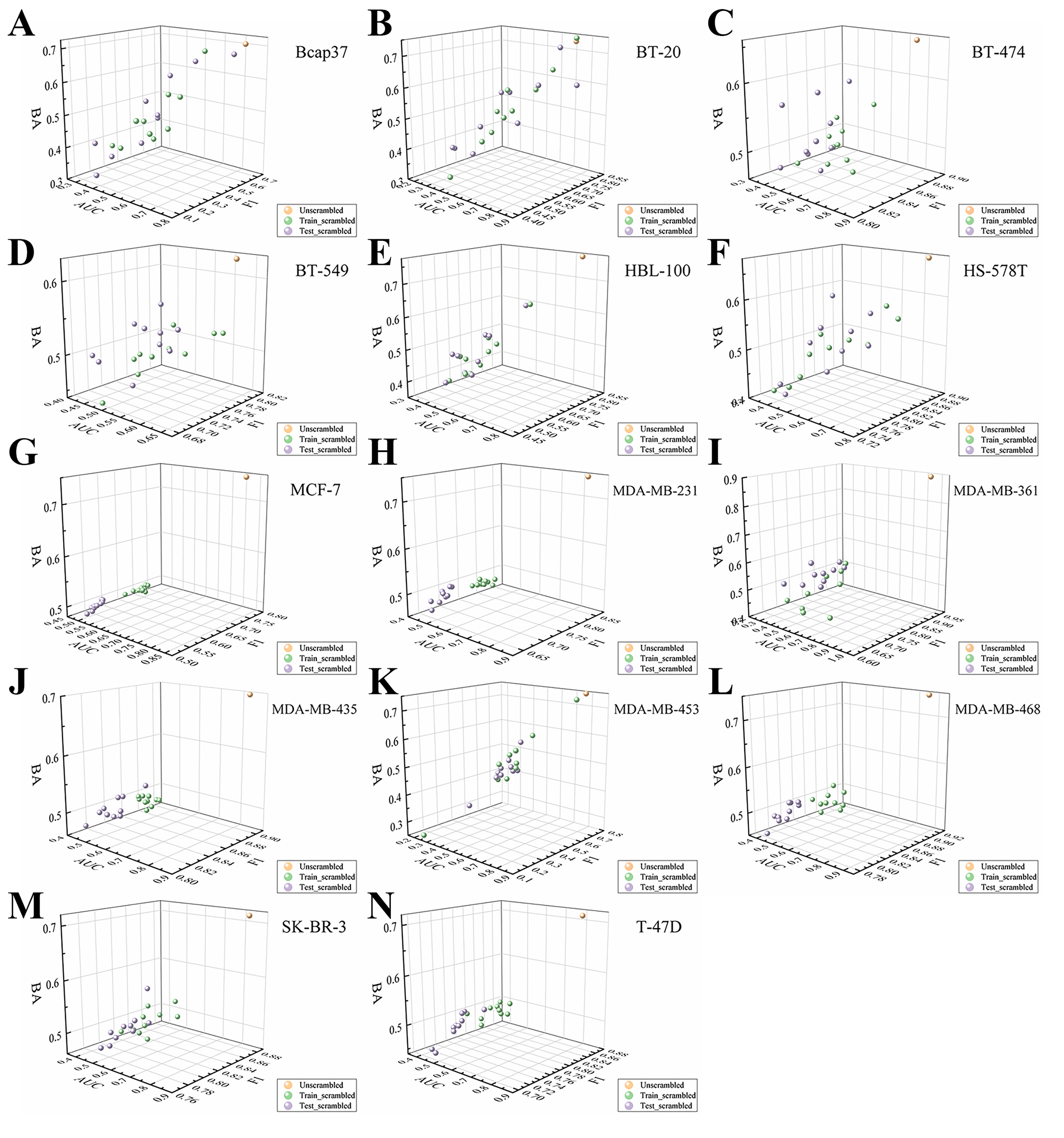
**

**Supplementary Figure S9.** Y-scrambling results for the XGB::Morgan models. Both the training sets and testing sets were unscrambled (gold). The training sets were scrambled, whereas the test sets were unscrambled (green). The training sets were unscrambled, whereas the test sets were scrambled (purple). F1: F1 scores. BA: Balanced accuracy. AUC: Area under the receiver operating characteristics curve.


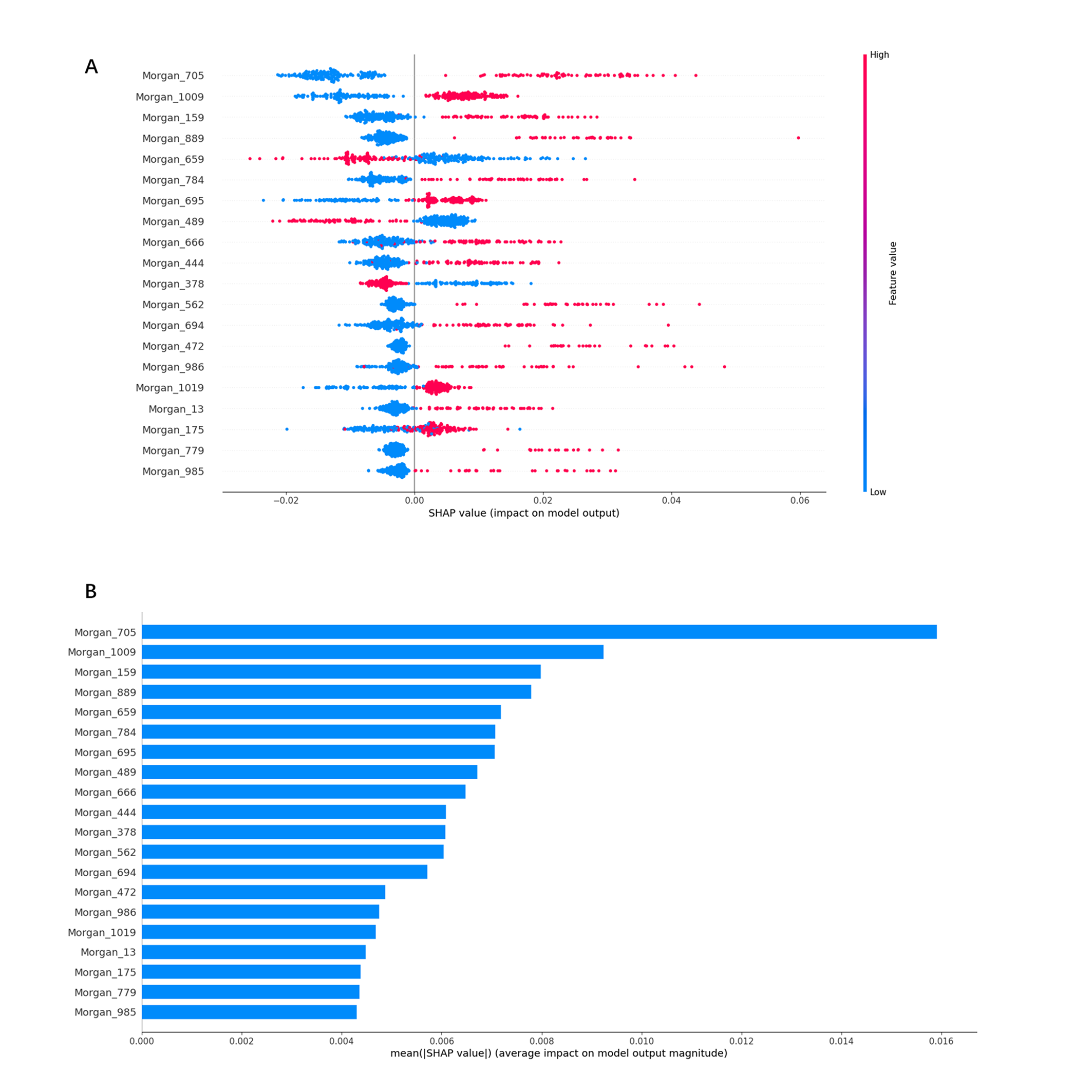


**Supplementary Figure S10.** Based on the top 20 most important features of the RF::Morgan model in Bcap37, (A) the SHAP values for each molecular substructure, and (B) the mean of the absolute value of the SHAP value for each molecular substructure.


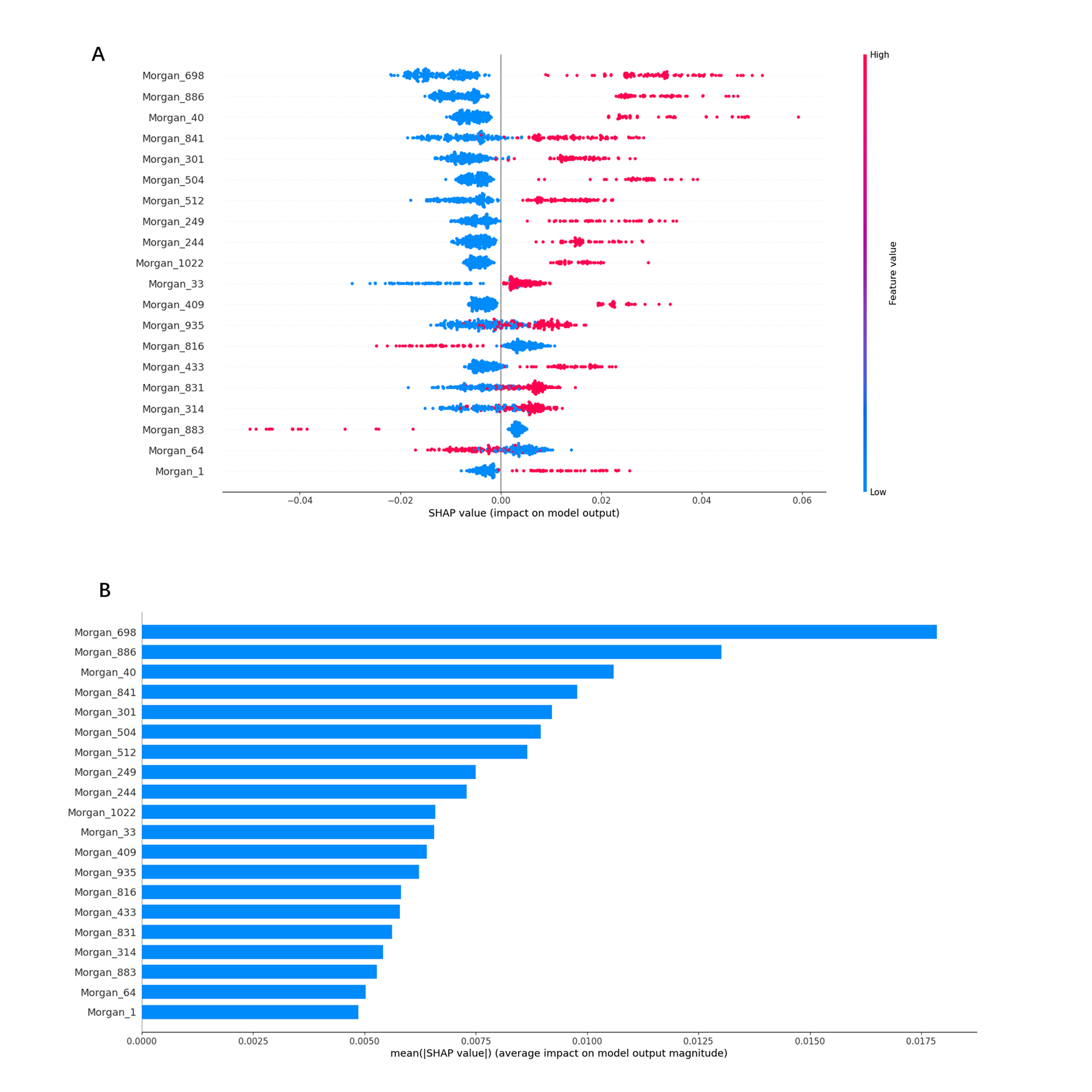


**Supplementary Figure S11.** Based on the top 20 most important features of the RF::Morgan model in BT-20, (A) the SHAP values for each molecular substructure, and (B) the mean of the absolute value of the SHAP value for each molecular substructure.


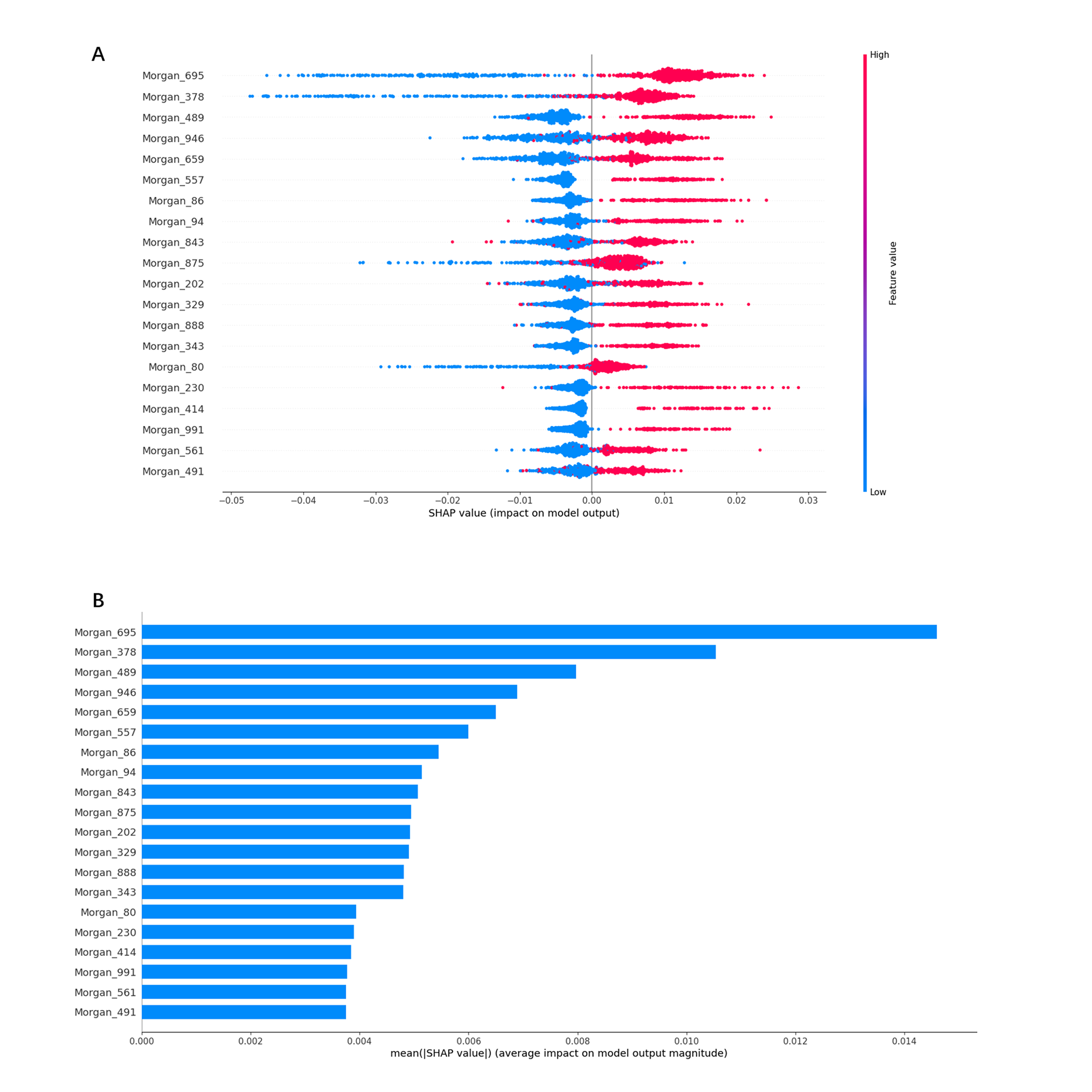


**Supplementary Figure S12.** Based on the top 20 most important features of the RF::Morgan model in BT-474, (A) the SHAP values for each molecular substructure, and (B) the mean of the absolute value of the SHAP value for each molecular substructure.


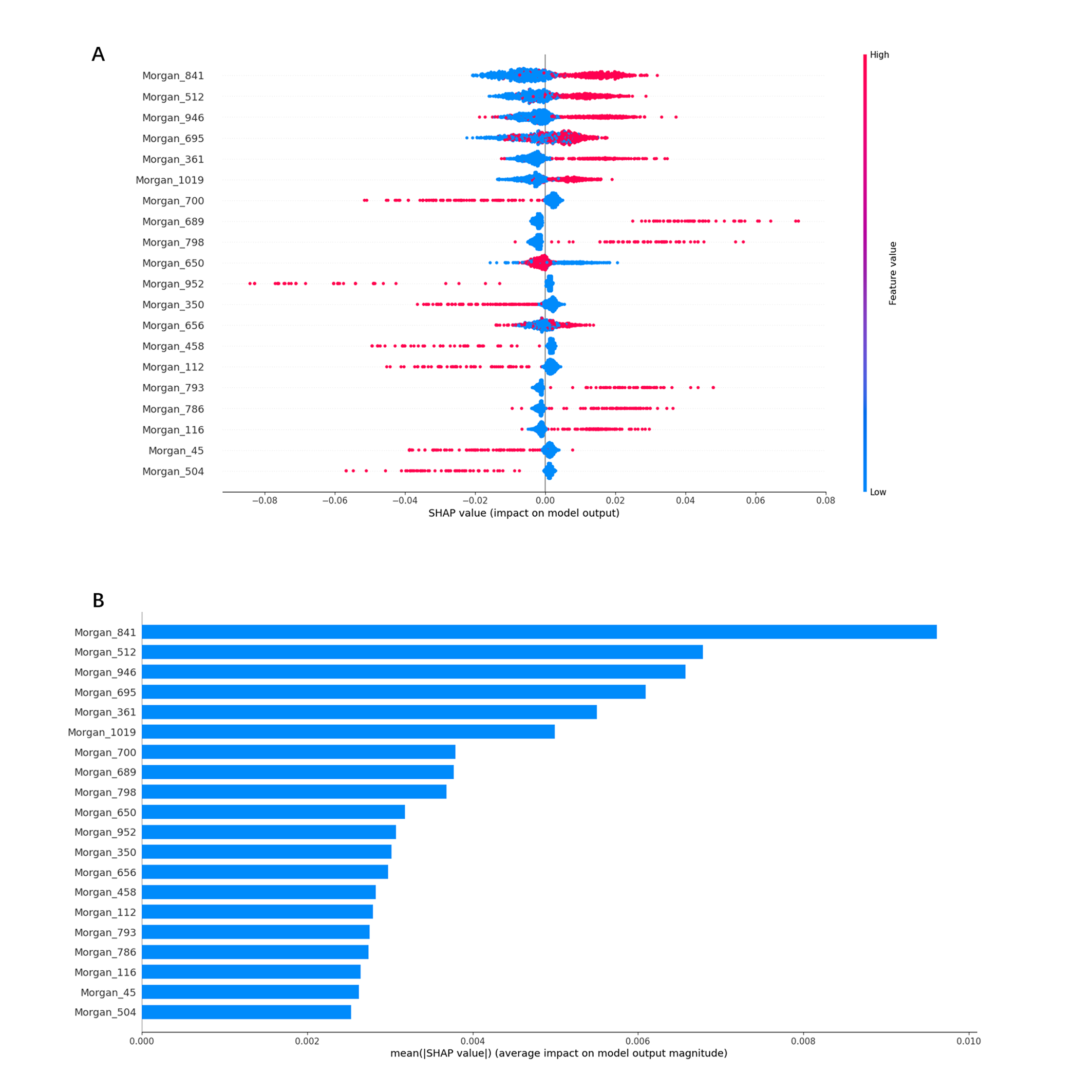


**Supplementary Figure S13.** Based on the top 20 most important features of the RF::Morgan model in BT-549, (A) the SHAP values for each molecular substructure, and (B) the mean of the absolute value of the SHAP value for each molecular substructure.


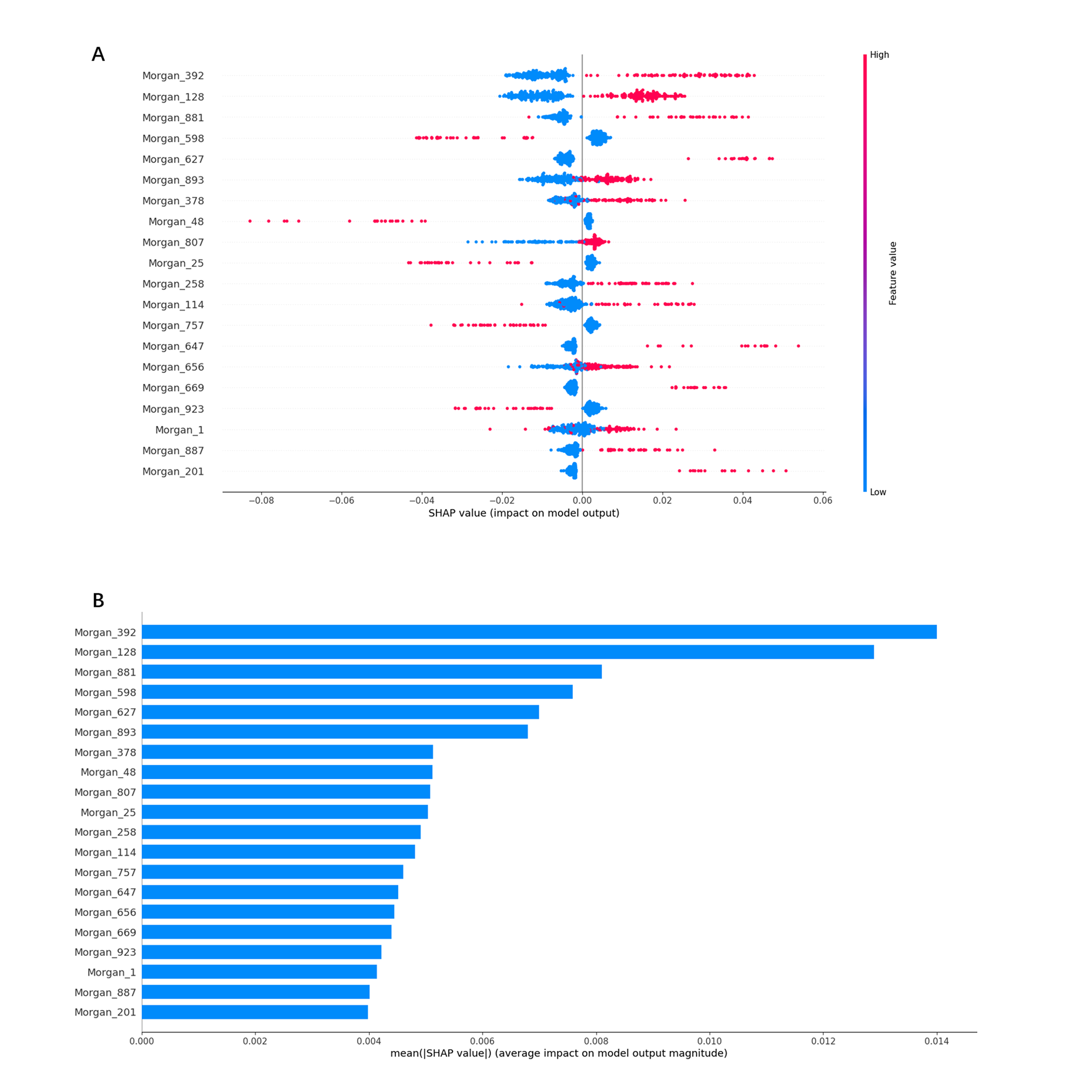


**Supplementary Figure S14.** Based on the top 20 most important features of the RF::Morgan model in HBL-100, (A) the SHAP values for each molecular substructure, and (B) the mean of the absolute value of the SHAP value for each molecular substructure.


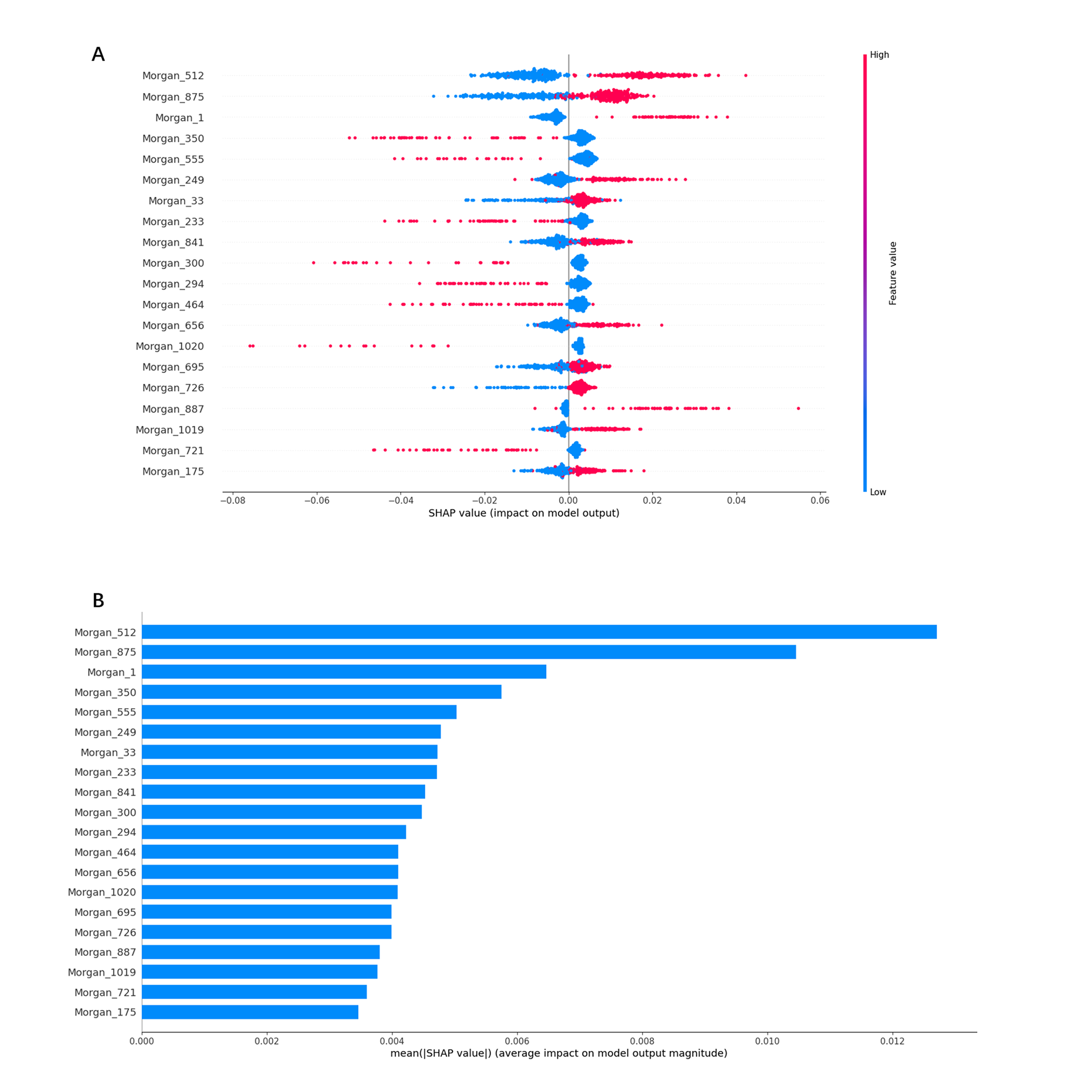


**Supplementary Figure S15.** Based on the top 20 most important features of the RF::Morgan model in HS-578T (A) the SHAP values for each molecular substructure, and (B) the mean of the absolute value of the SHAP value for each molecular substructure.


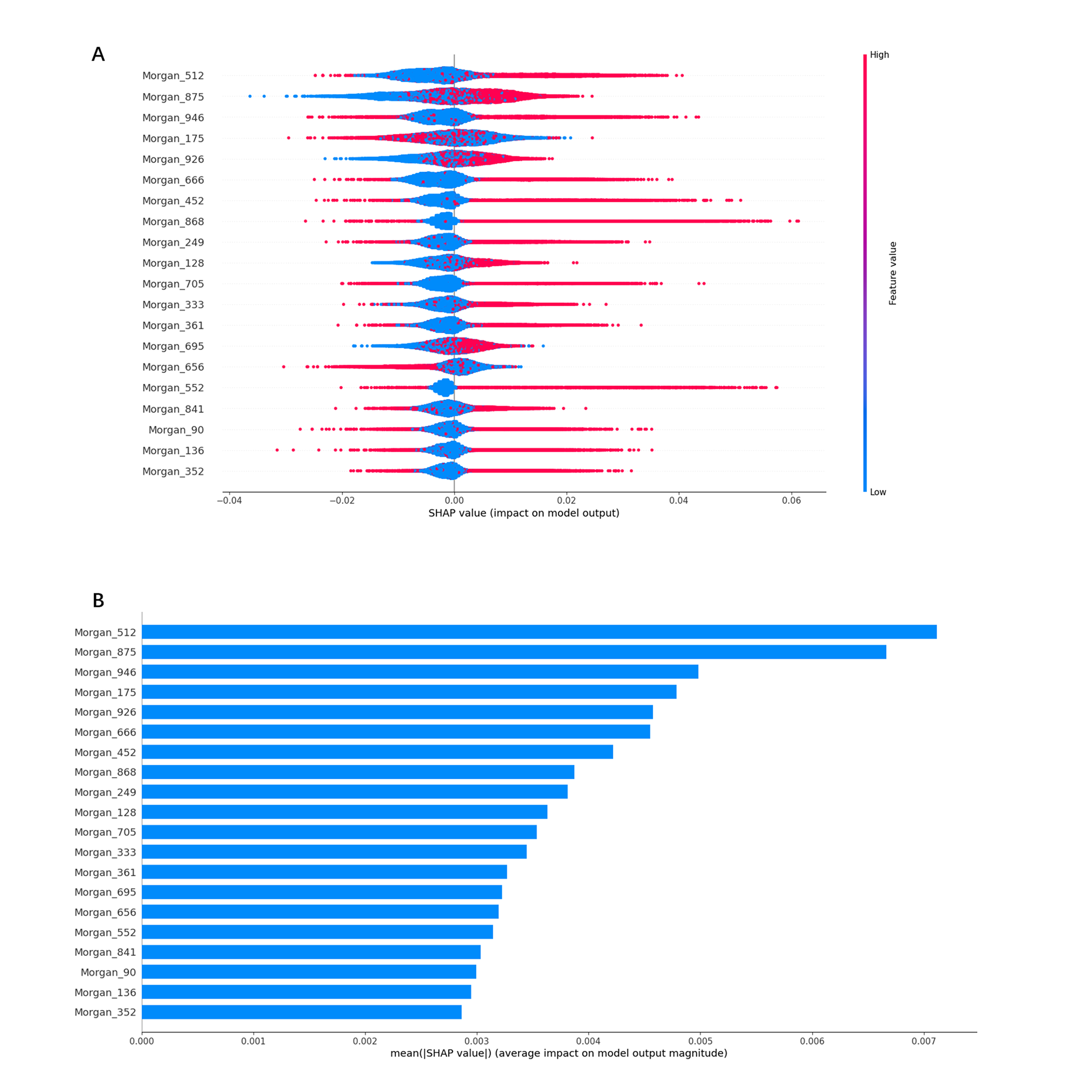


**Supplementary Figure S16.** Based on the top 20 most important features of the RF::Morgan model in MCF-7 (A) the SHAP values for each molecular substructure, and (B) the mean of the absolute value of the SHAP value for each molecular substructure.


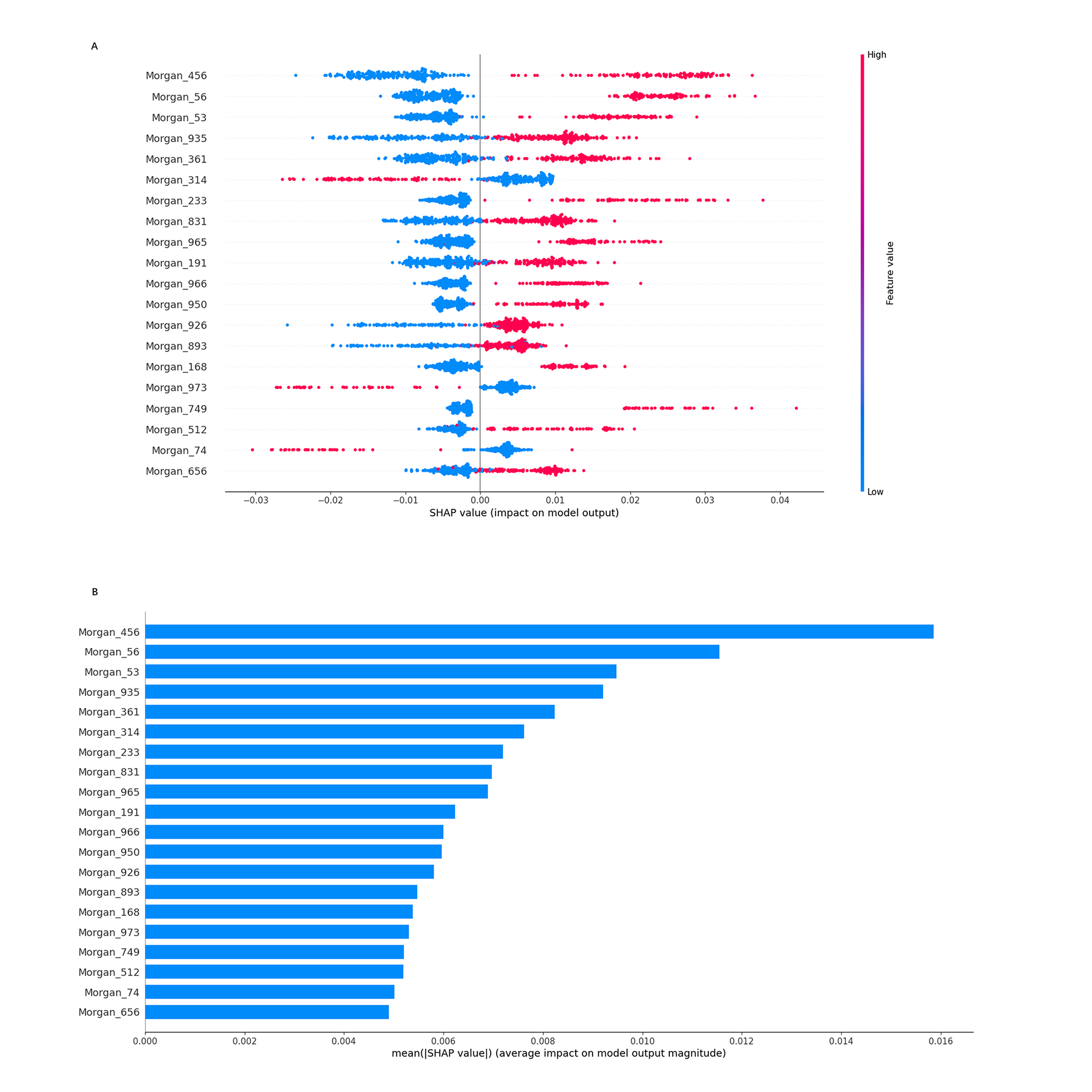


**Supplementary Figure S17.** Based on the top 20 most important features of the RF::Morgan model in MDA-MB-361 (A) the SHAP values for each molecular substructure, and (B) the mean of the absolute value of the SHAP value for each molecular substructure.


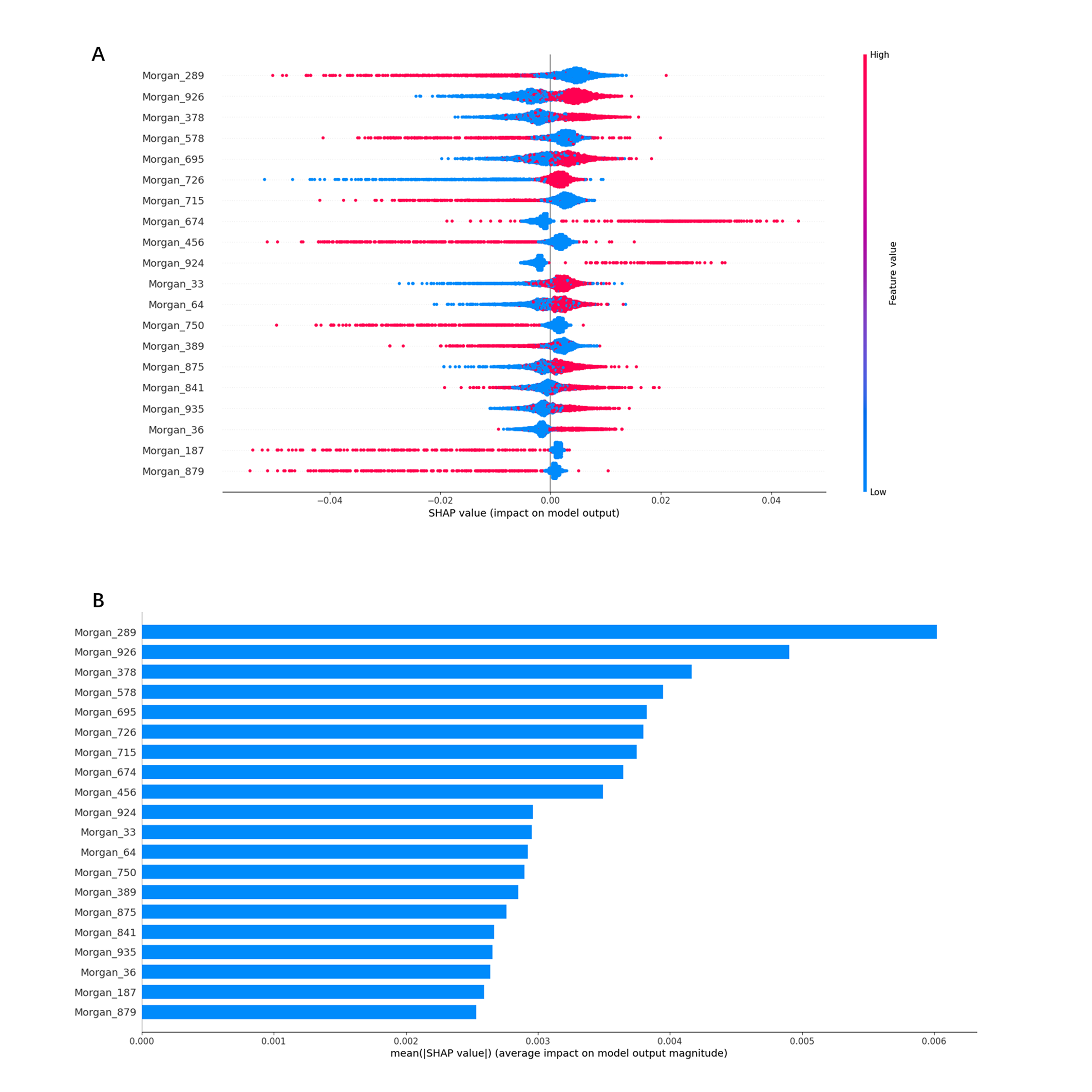


**Supplementary Figure S18.** Based on the top 20 most important features of the RF::Morgan model in MDA-MB-435 (A) the SHAP values for each molecular substructure, and (B) the mean of the absolute value of the SHAP value for each molecular substructure.


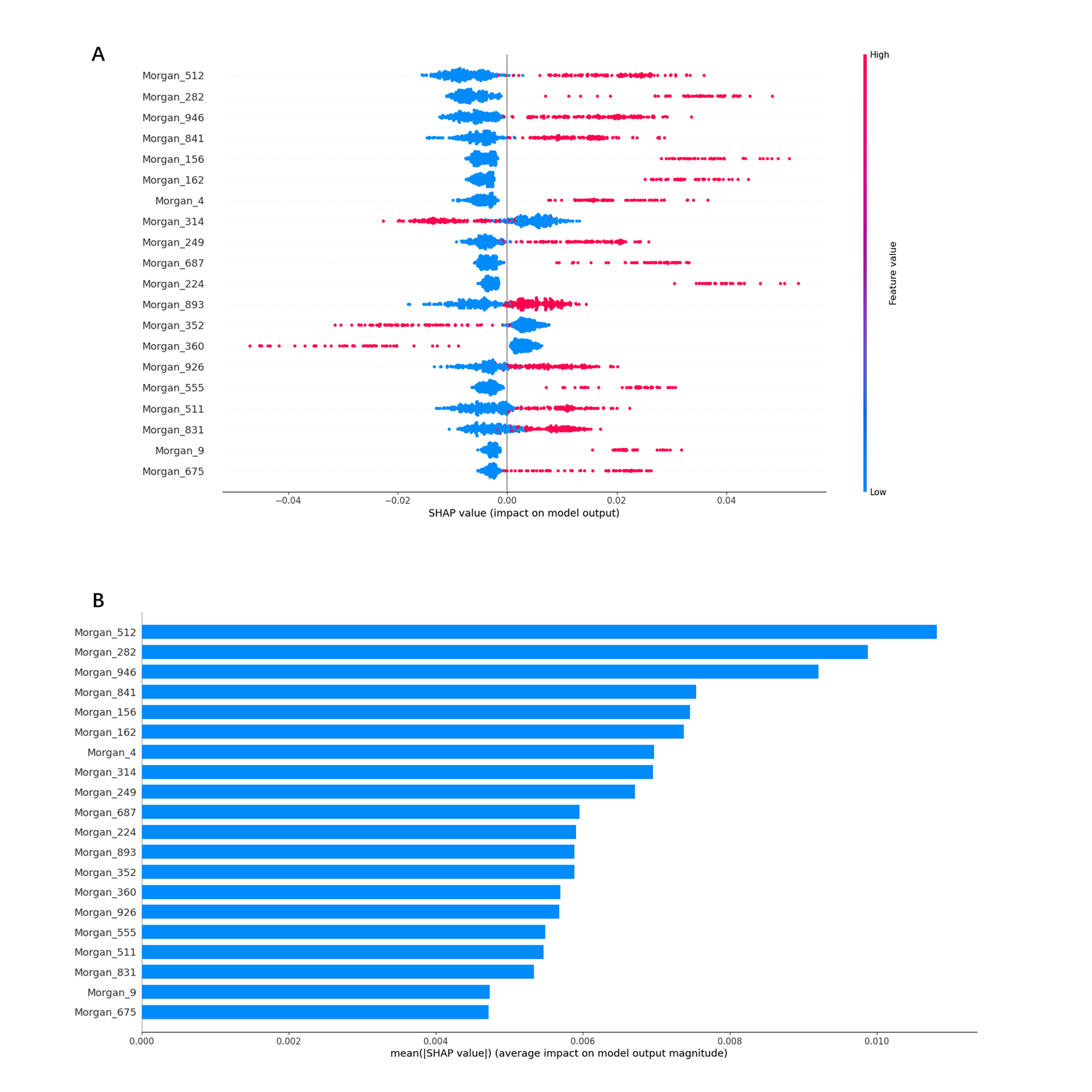


**Supplementary Figure S19.** Based on the top 20 most important features of the RF::Morgan model in MDA-MB-453 (A) the SHAP values for each molecular substructure, and (B) the mean of the absolute value of the SHAP value for each molecular substructure.


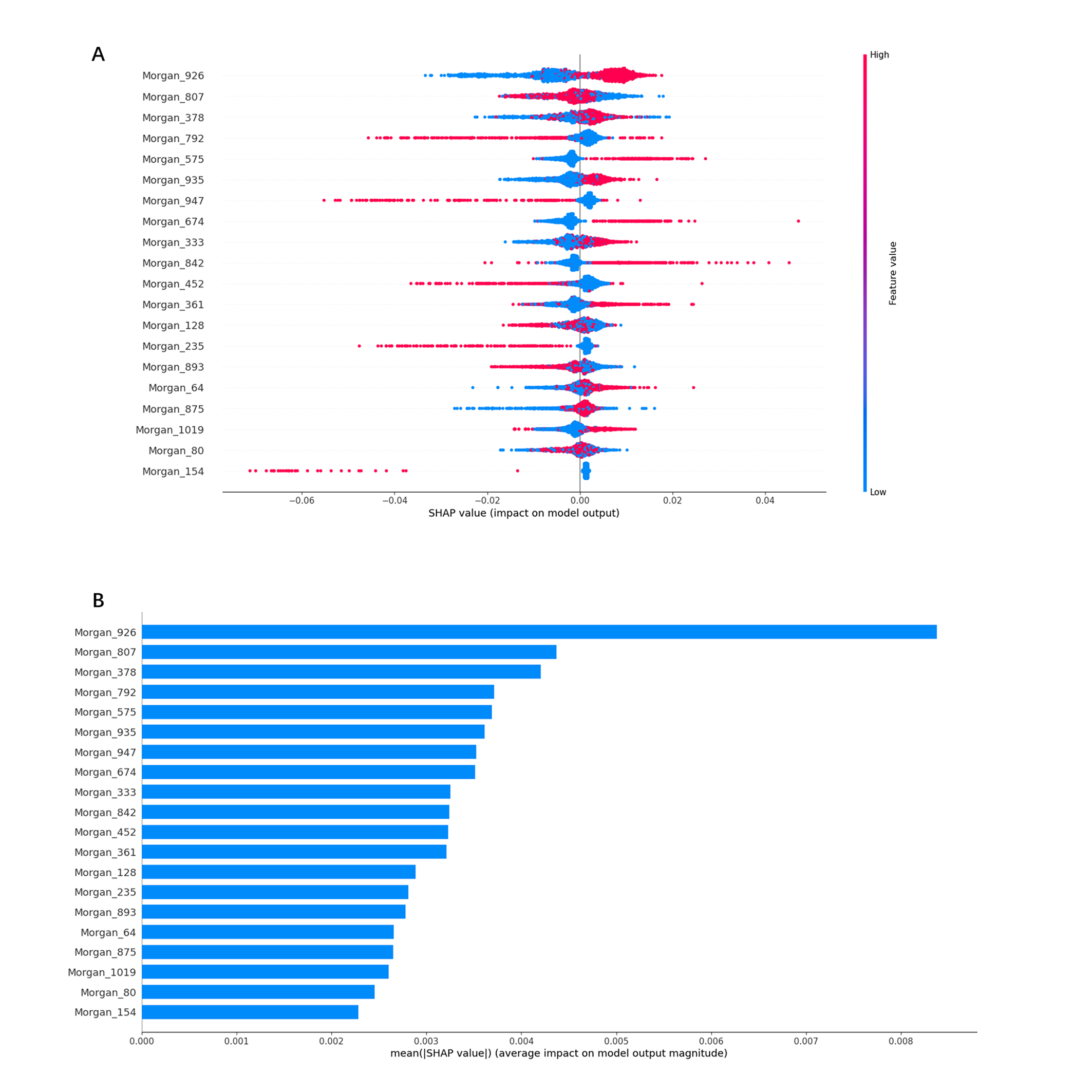


**Supplementary Figure S20.** Based on the top 20 most important features of the RF::Morgan model in MDA-MB-468 (A) the SHAP values for each molecular substructure, and (B) the mean of the absolute value of the SHAP value for each molecular substructure.


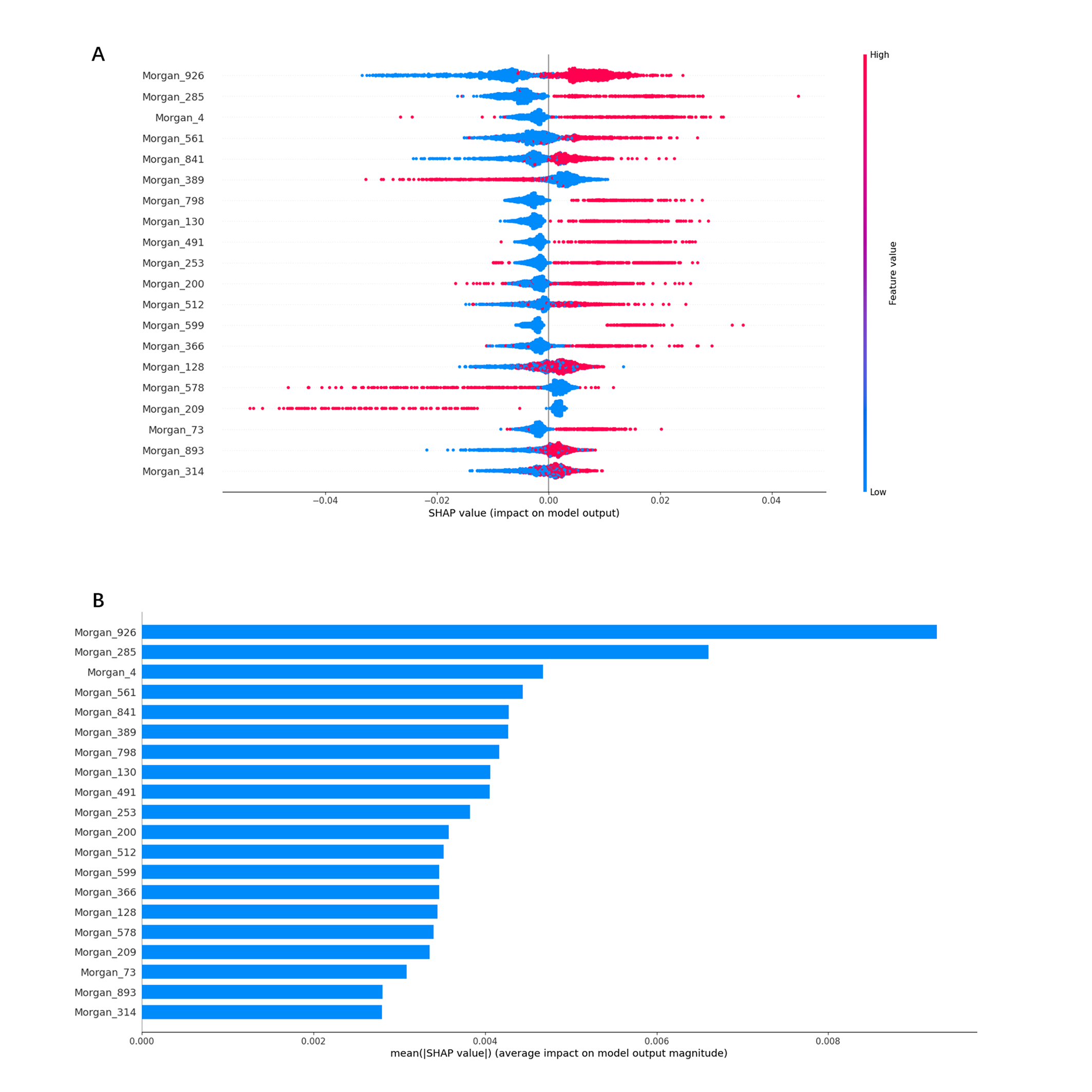


**Supplementary Figure S21.** Based on the top 20 most important features of the RF::Morgan model in SK-BR-3 (A) the SHAP values for each molecular substructure, and (B) the mean of the absolute value of the SHAP value for each molecular substructure.


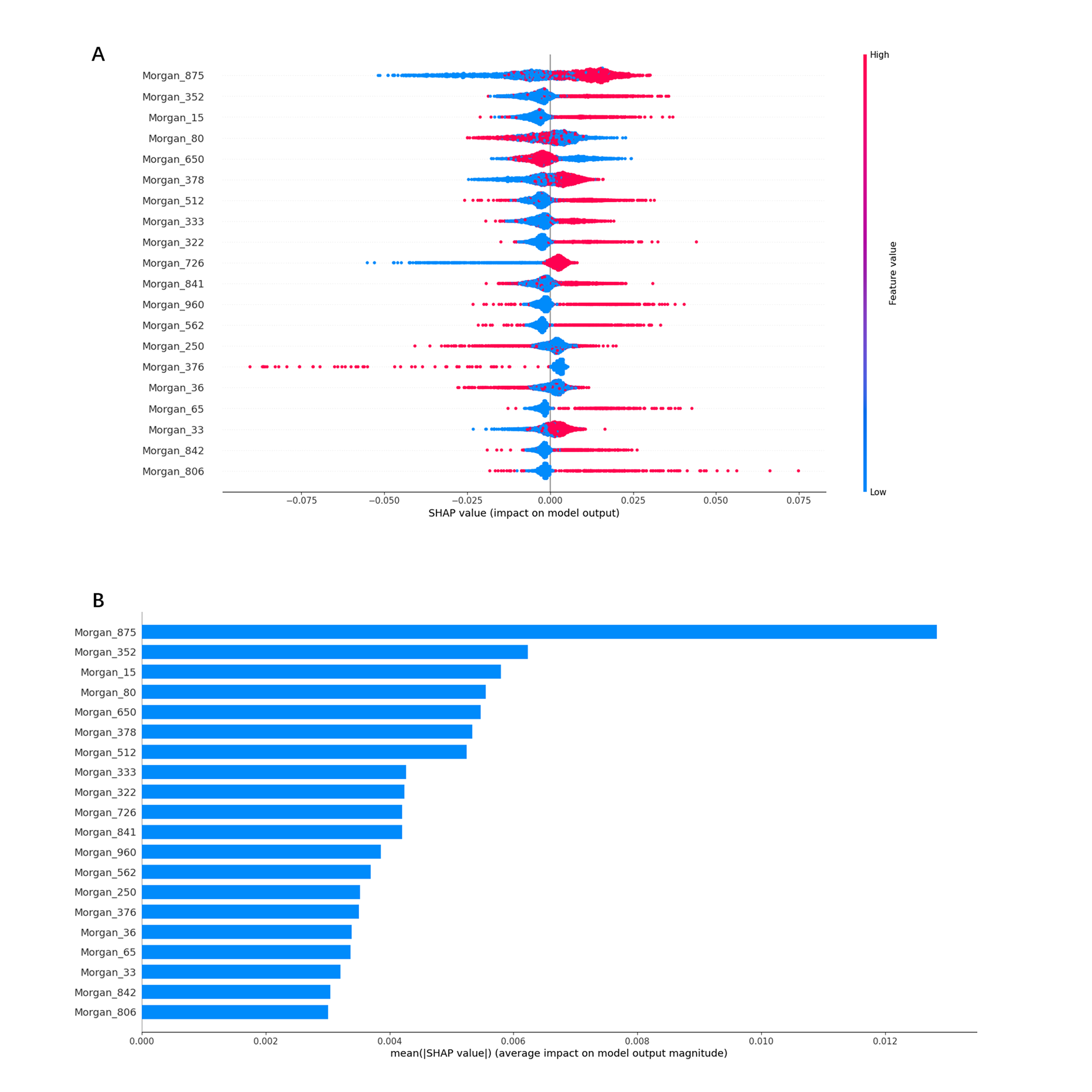


**Supplementary Figure S22.** Based on the top 20 most important features of the RF::Morgan model in T-47D (A) the SHAP values for each molecular substructure, and (B) the mean of the absolute value of the SHAP value for each molecular substructure.


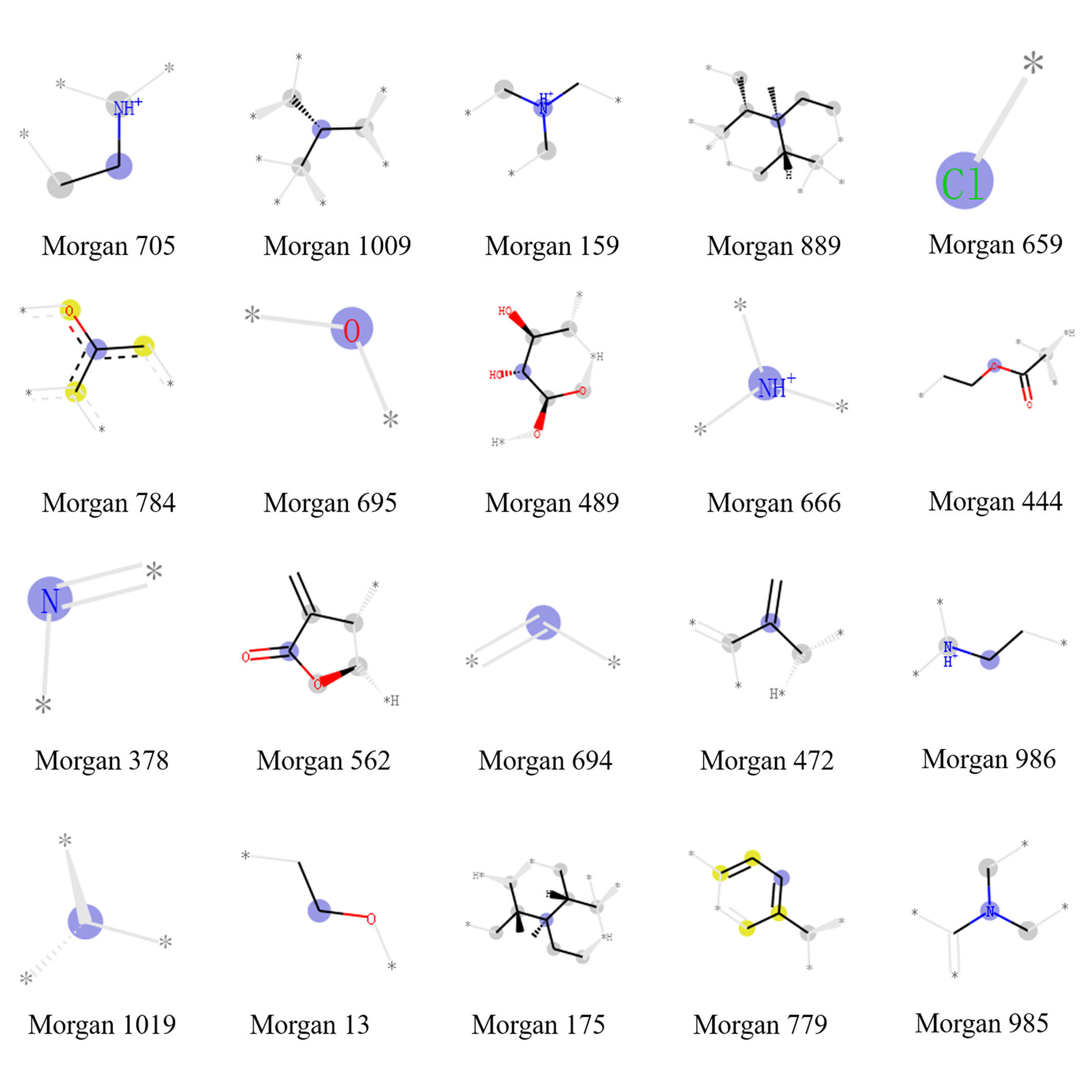


**Supplementary Figure S23.** Important molecular substructures of the RF::Morgan model in Bcap37.


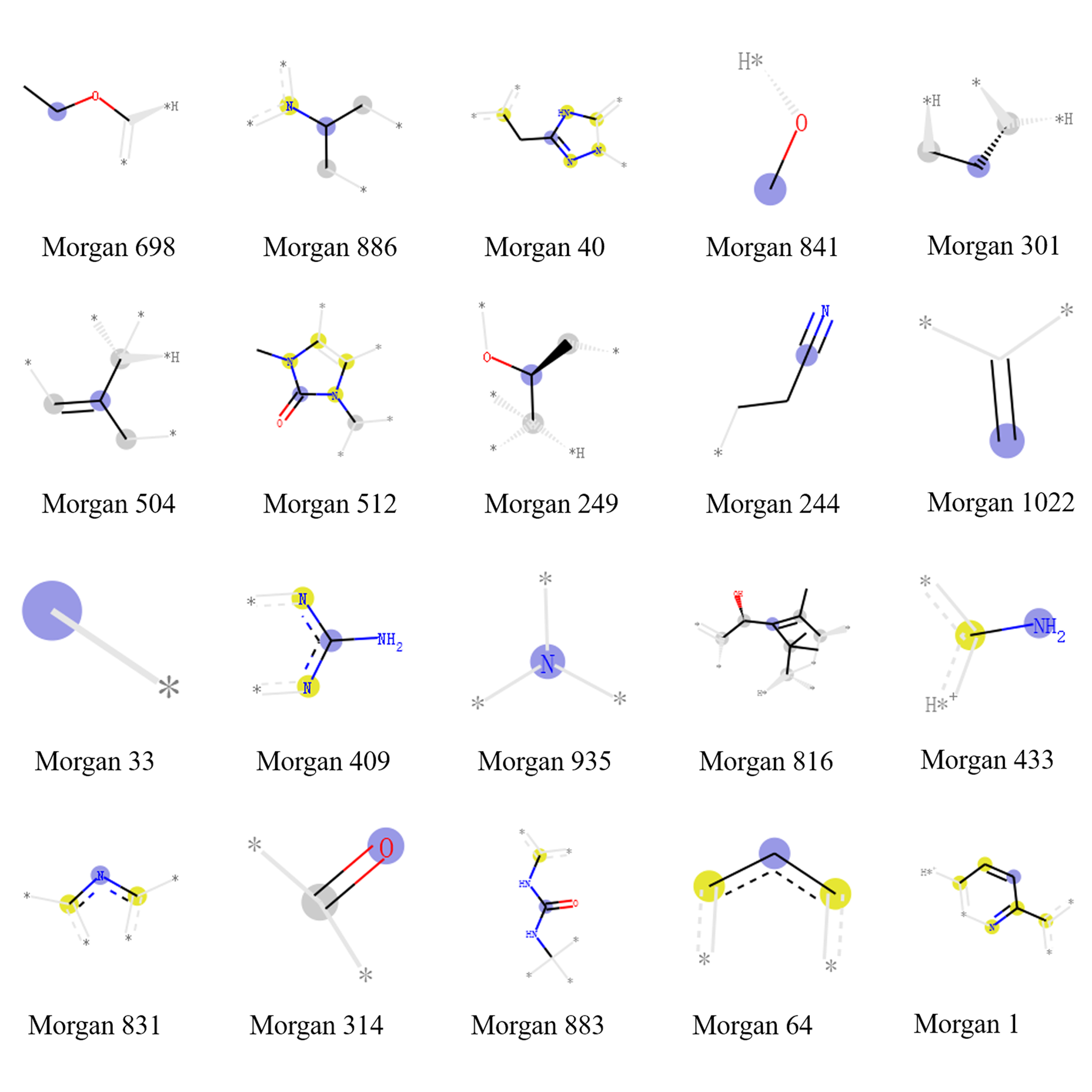


**Supplementary Figure S24.** Important molecular substructures of the RF::Morgan model in BT-20.


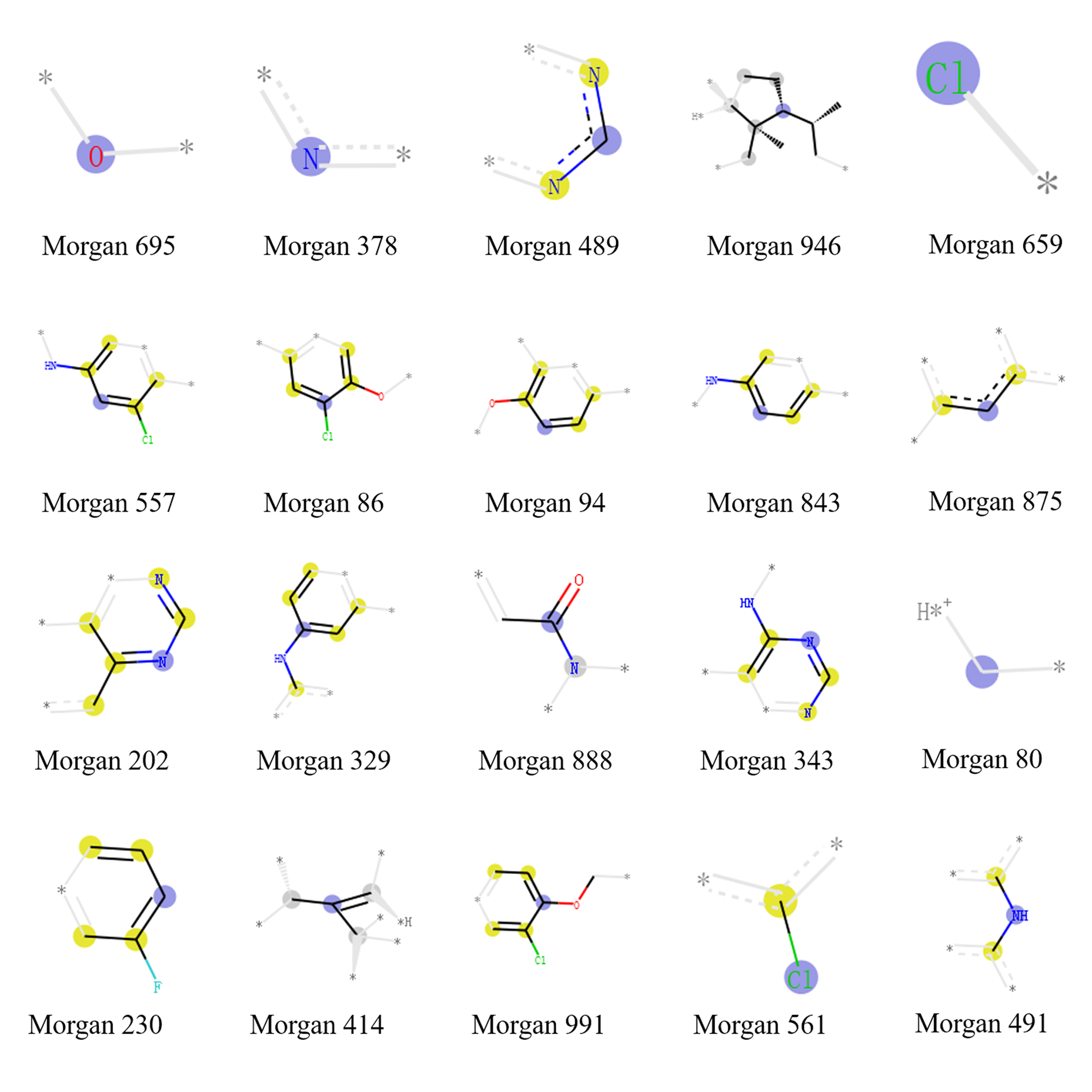


**Supplementary Figure S25.** Important molecular substructures of the RF::Morgan model in BT-474.


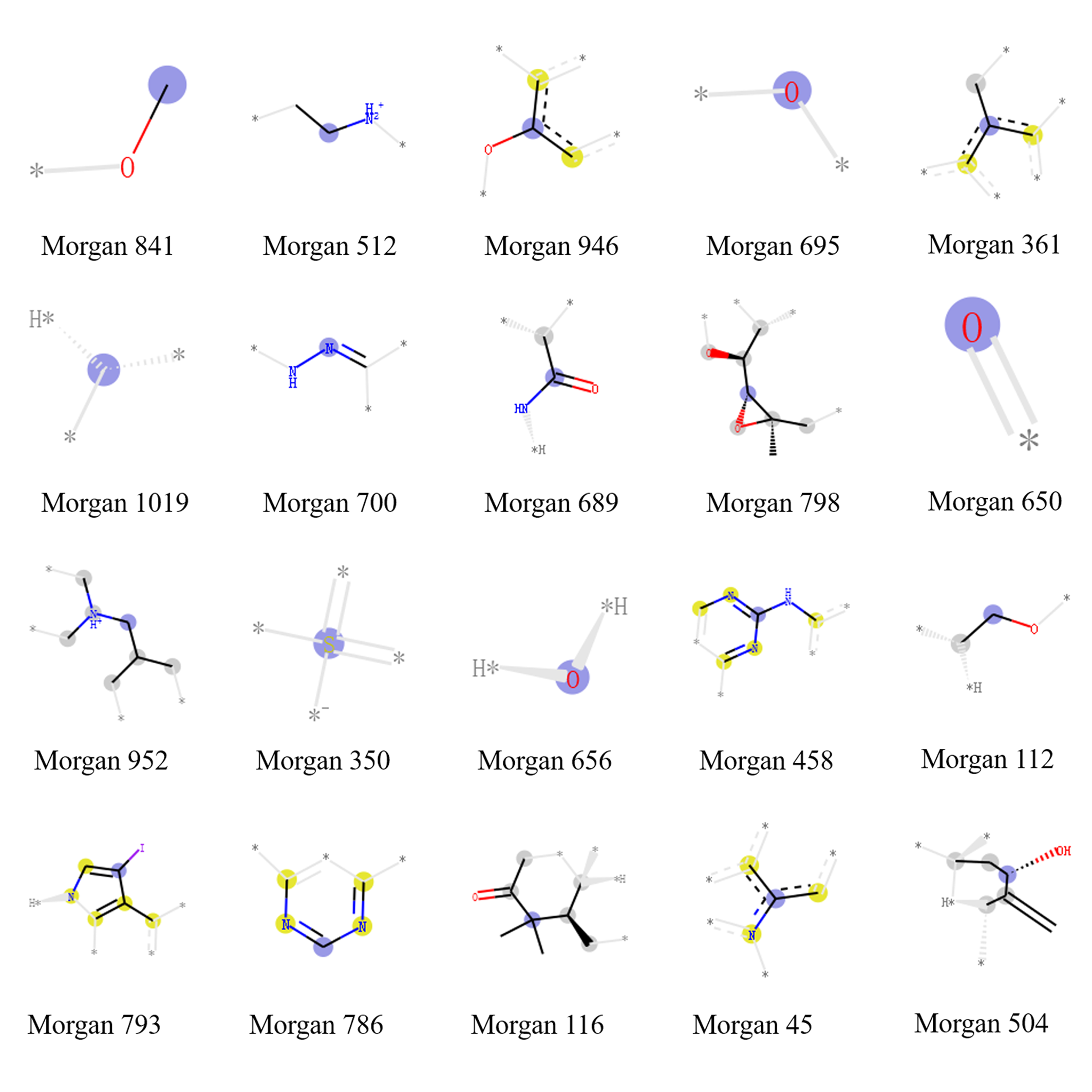


**Supplementary Figure S26.** Important molecular substructures of the RF::Morgan model in BT-549.


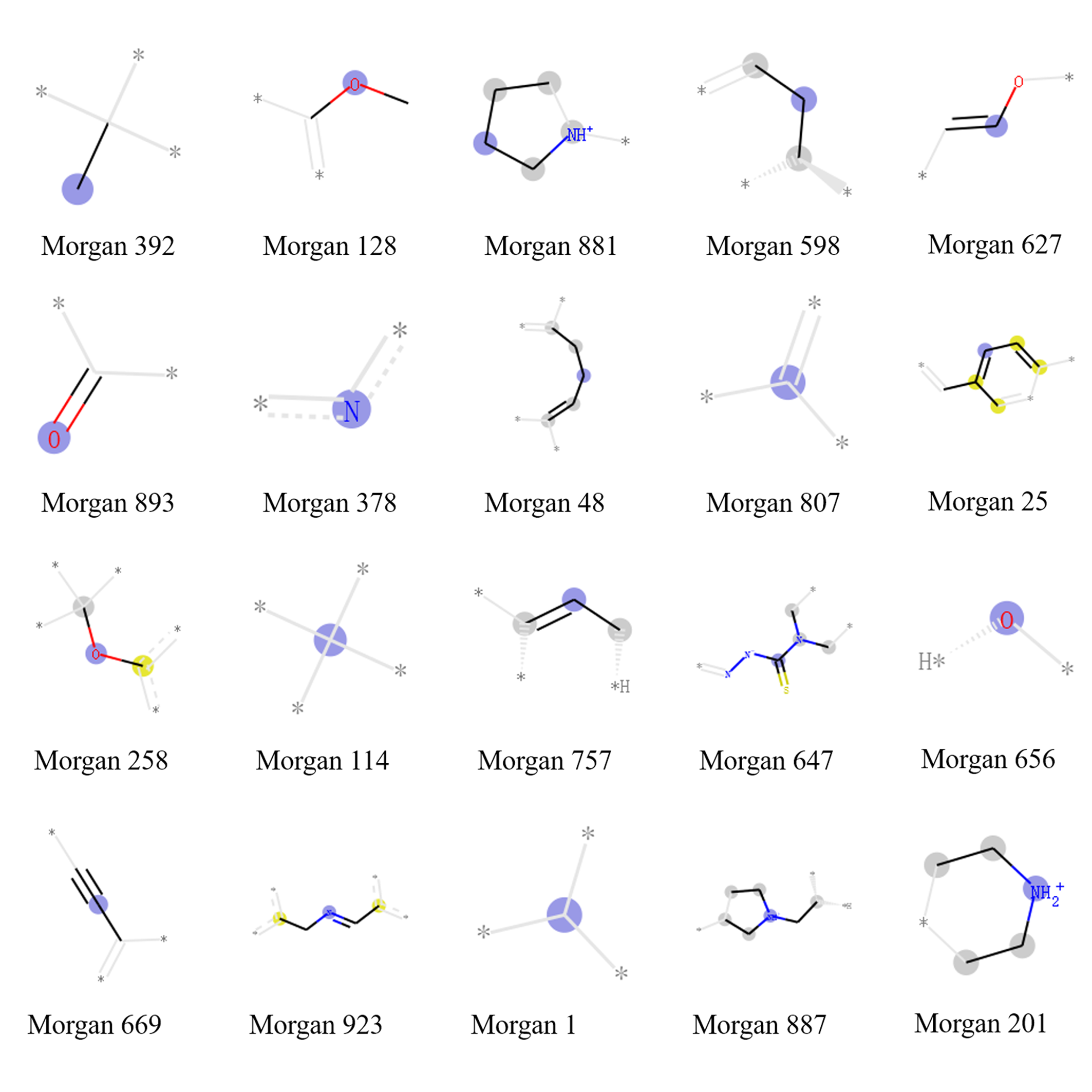


**Supplementary Figure S27.** Important molecular substructures of the RF::Morgan model in HBL-100.


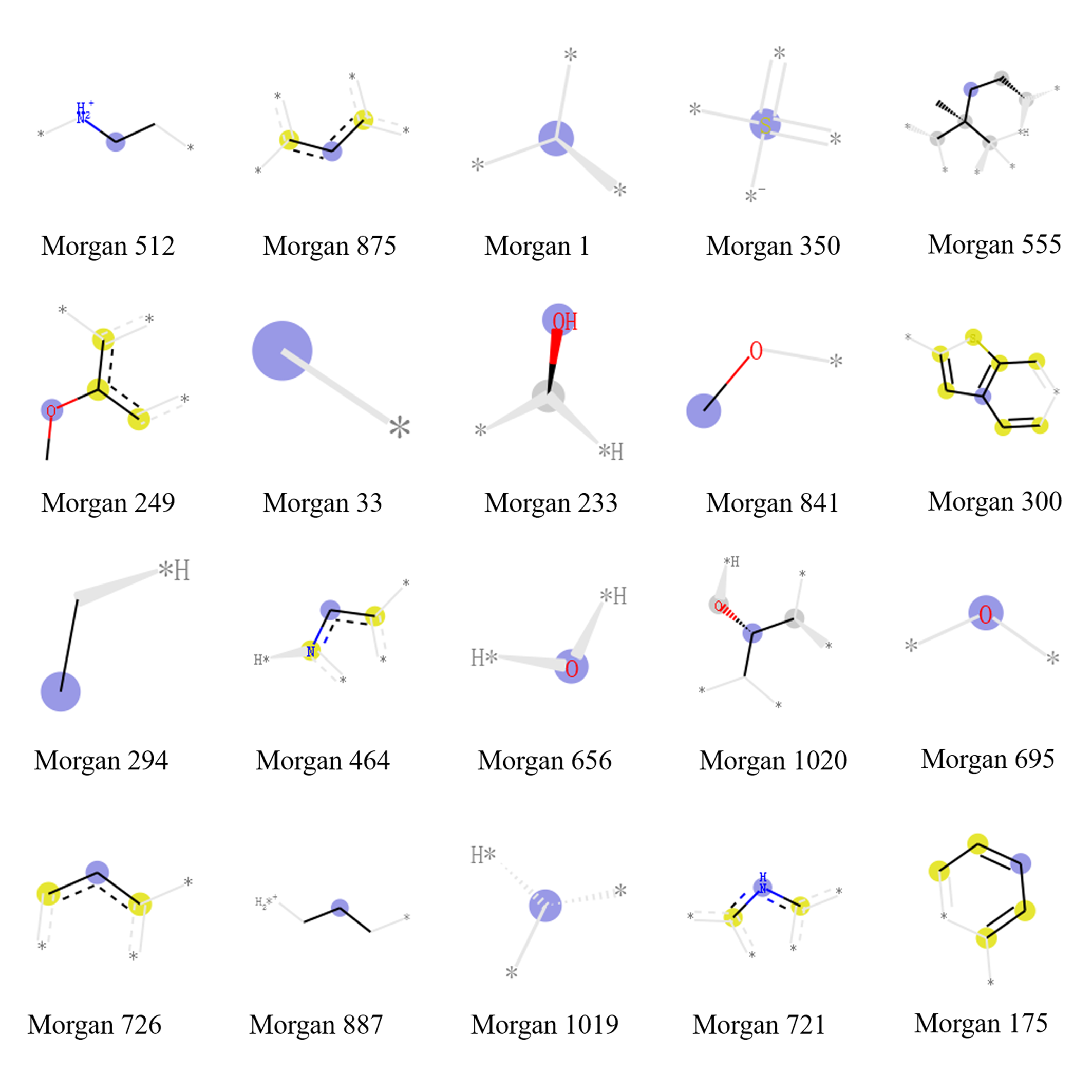


**Supplementary Figure S28.** Important molecular substructures of the RF::Morgan model in HS-578T.


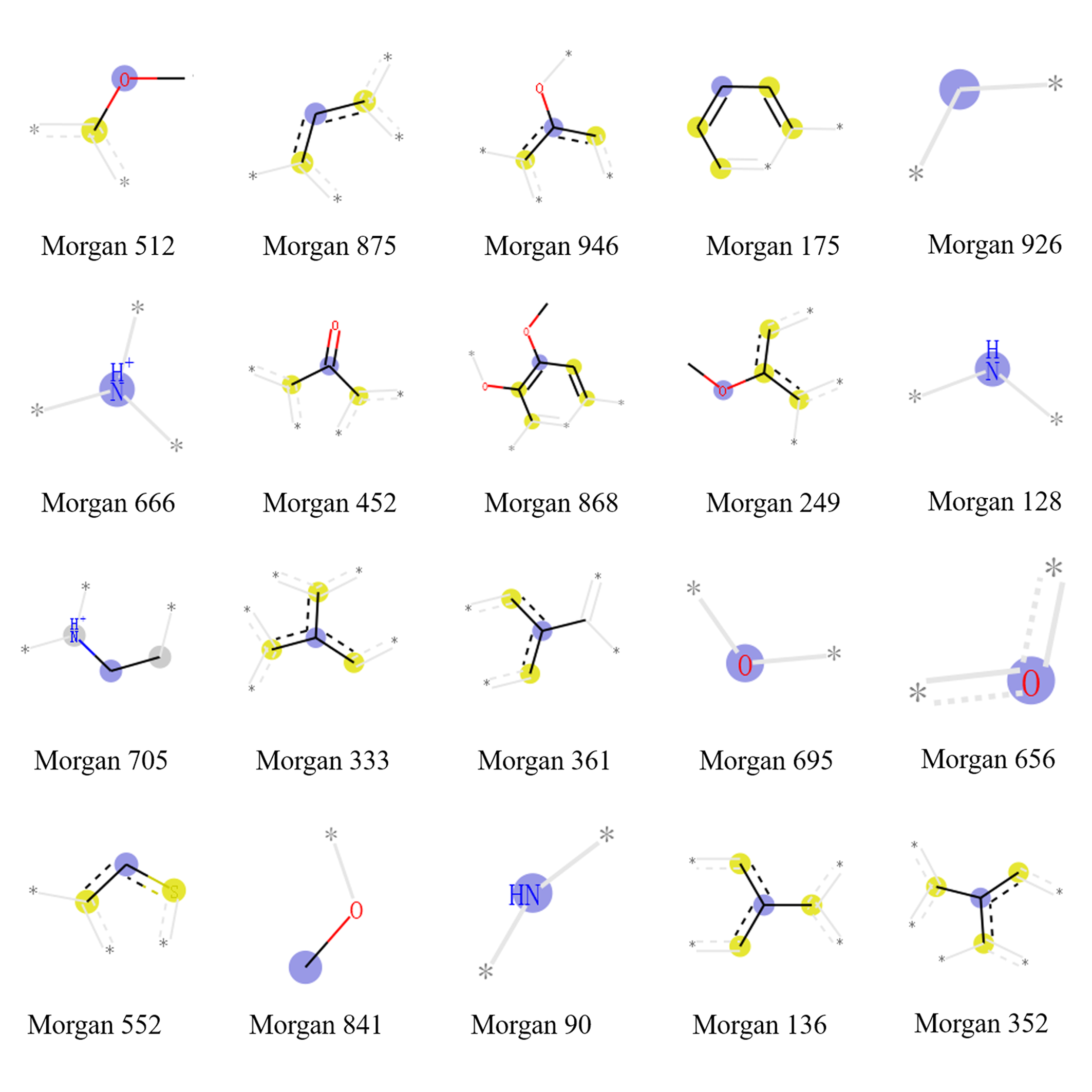


**Supplementary Figure S29.** Important molecular substructures of the RF::Morgan model in MCF-7.


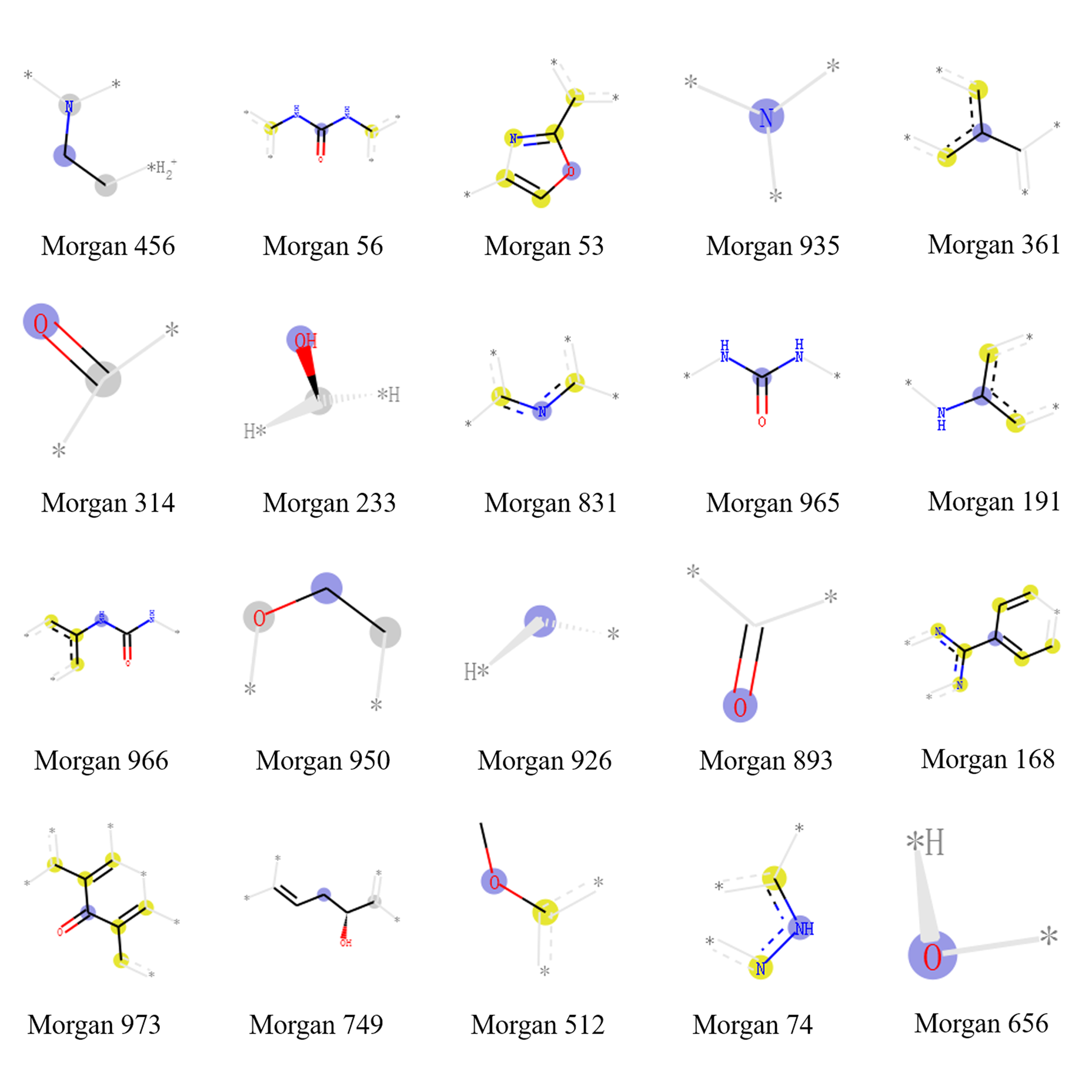


**Supplementary Figure S30.** Important molecular substructures of the RF::Morgan model in MDA-MB-361.


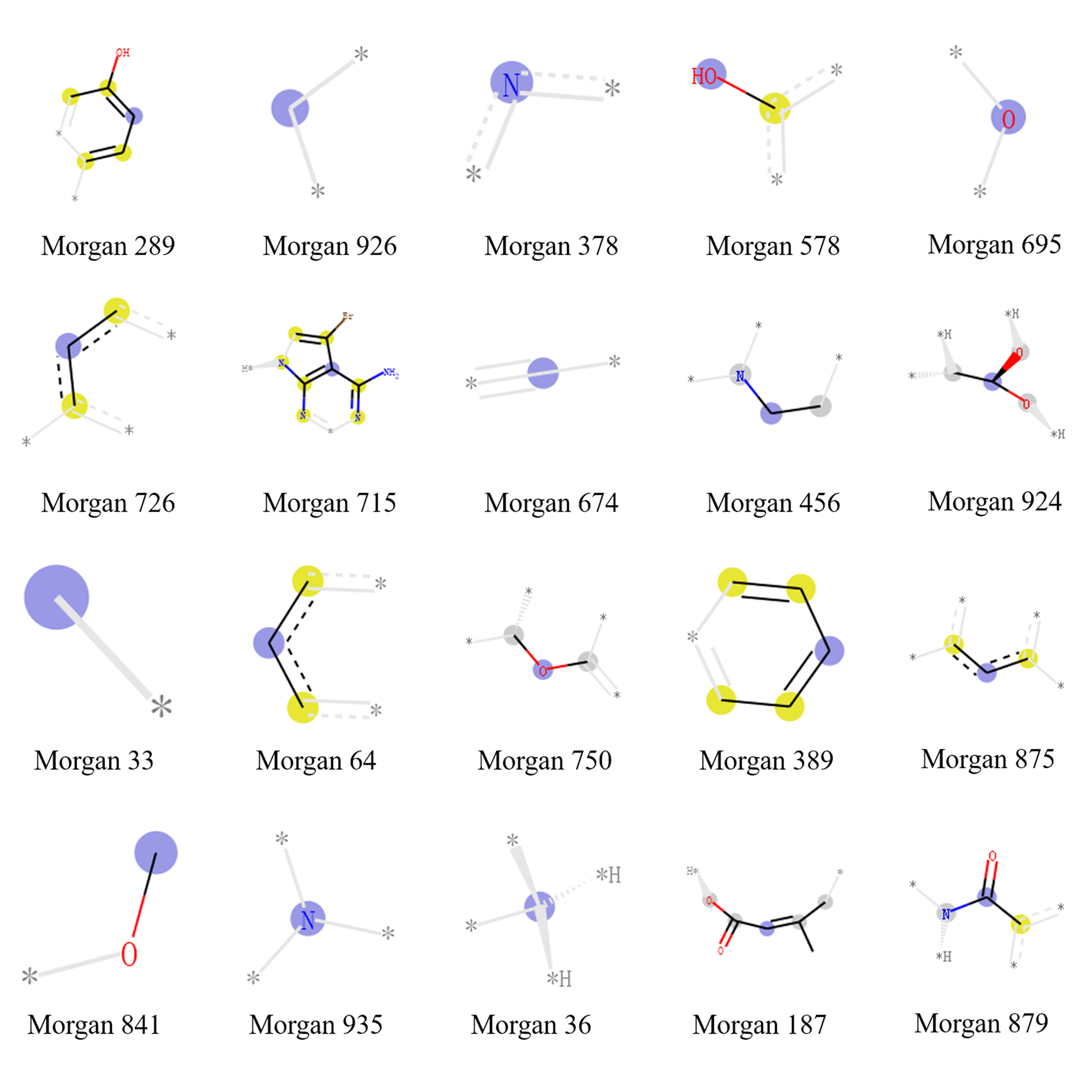


**Supplementary Figure S31.** Important molecular substructures of the RF::Morgan model in MDA-MB-435.


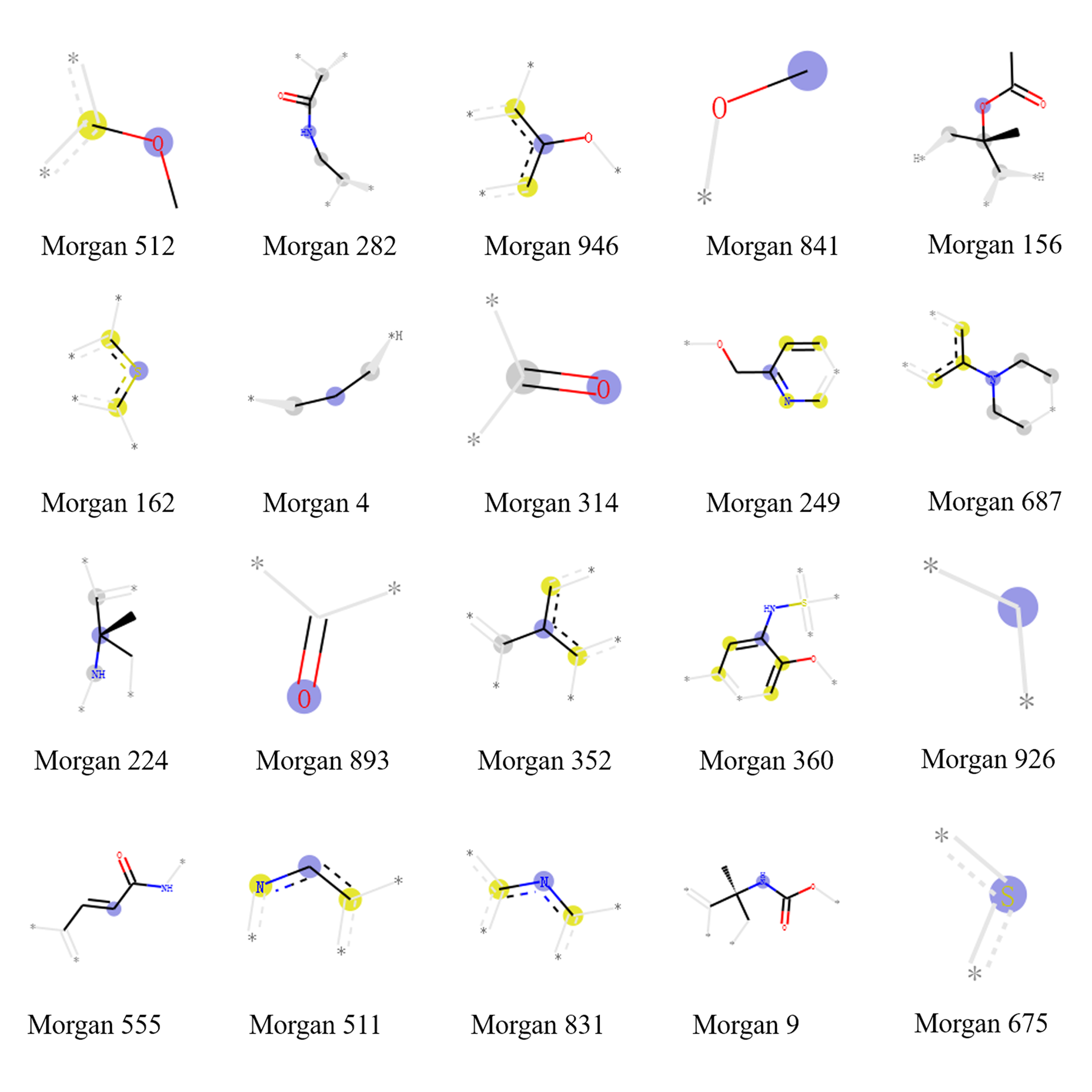


**Supplementary Figure S32.** Important molecular substructures of the RF::Morgan model in MDA-MB-453.


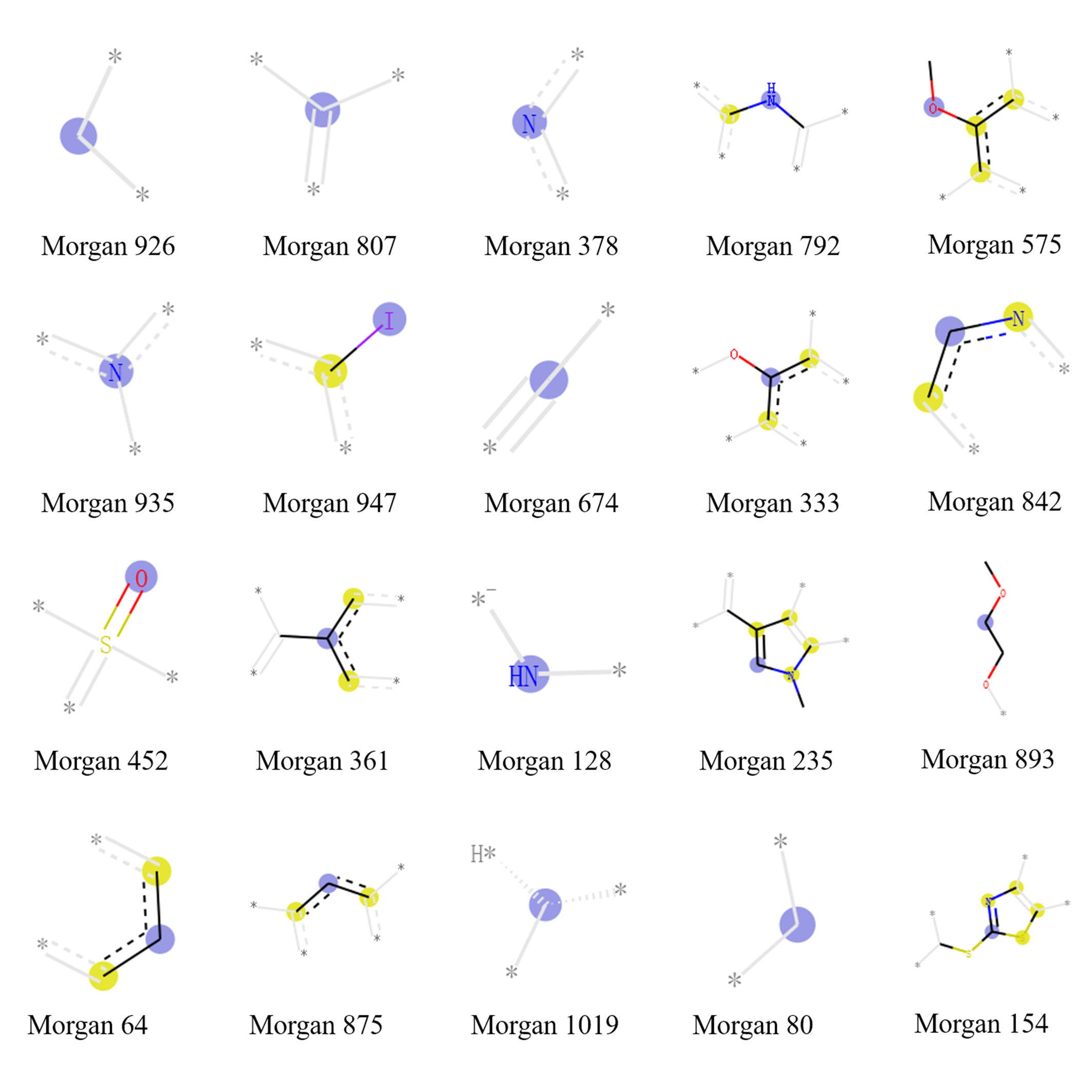


**Supplementary Figure S33.** Important molecular substructures of the RF::Morgan model in MDA-MB-468.


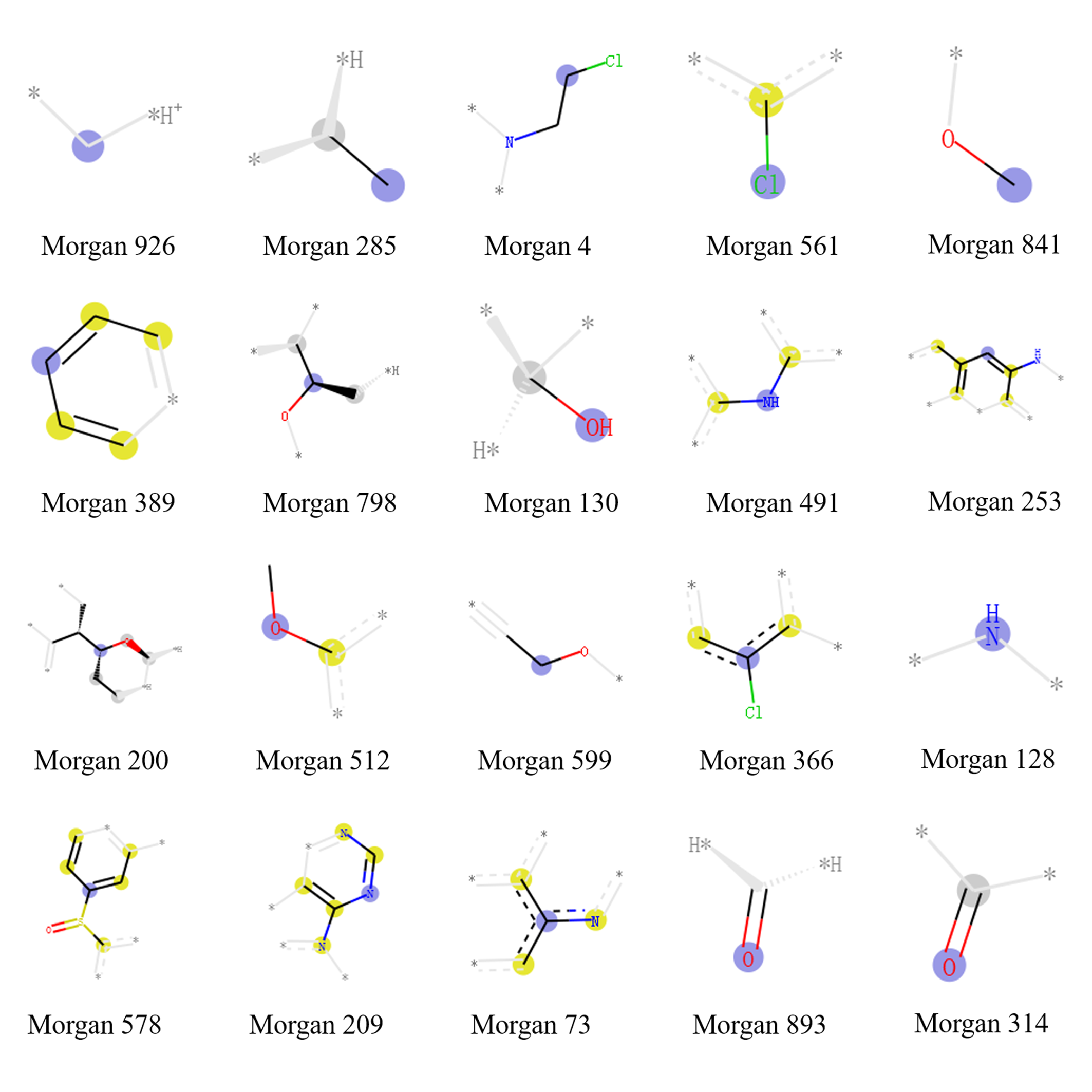


**Supplementary Figure S34.** Important molecular substructures of the RF::Morgan model in SK-BR-3.


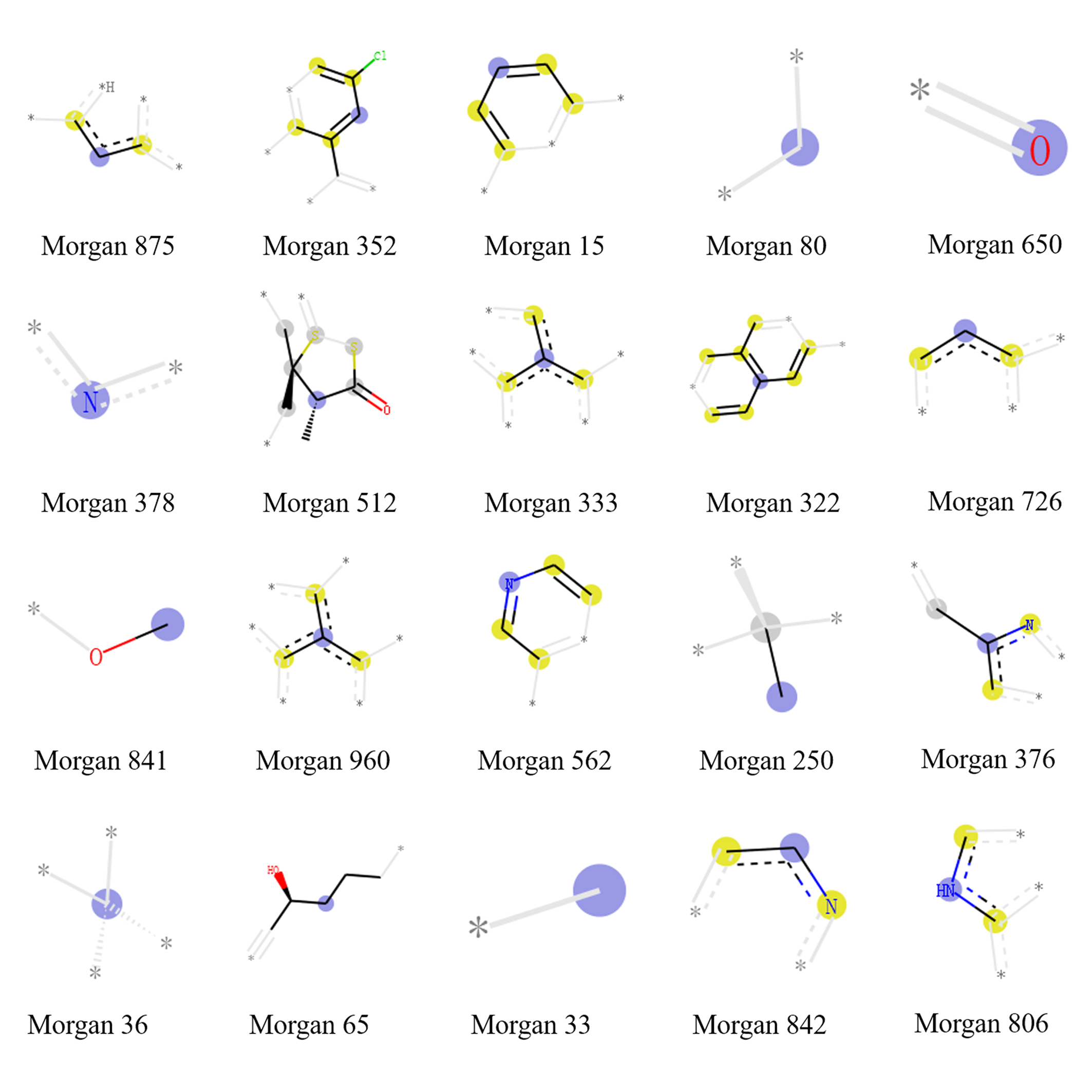


**Supplementary Figure S35.** Important molecular substructures of the RF::Morgan model in T-47D.


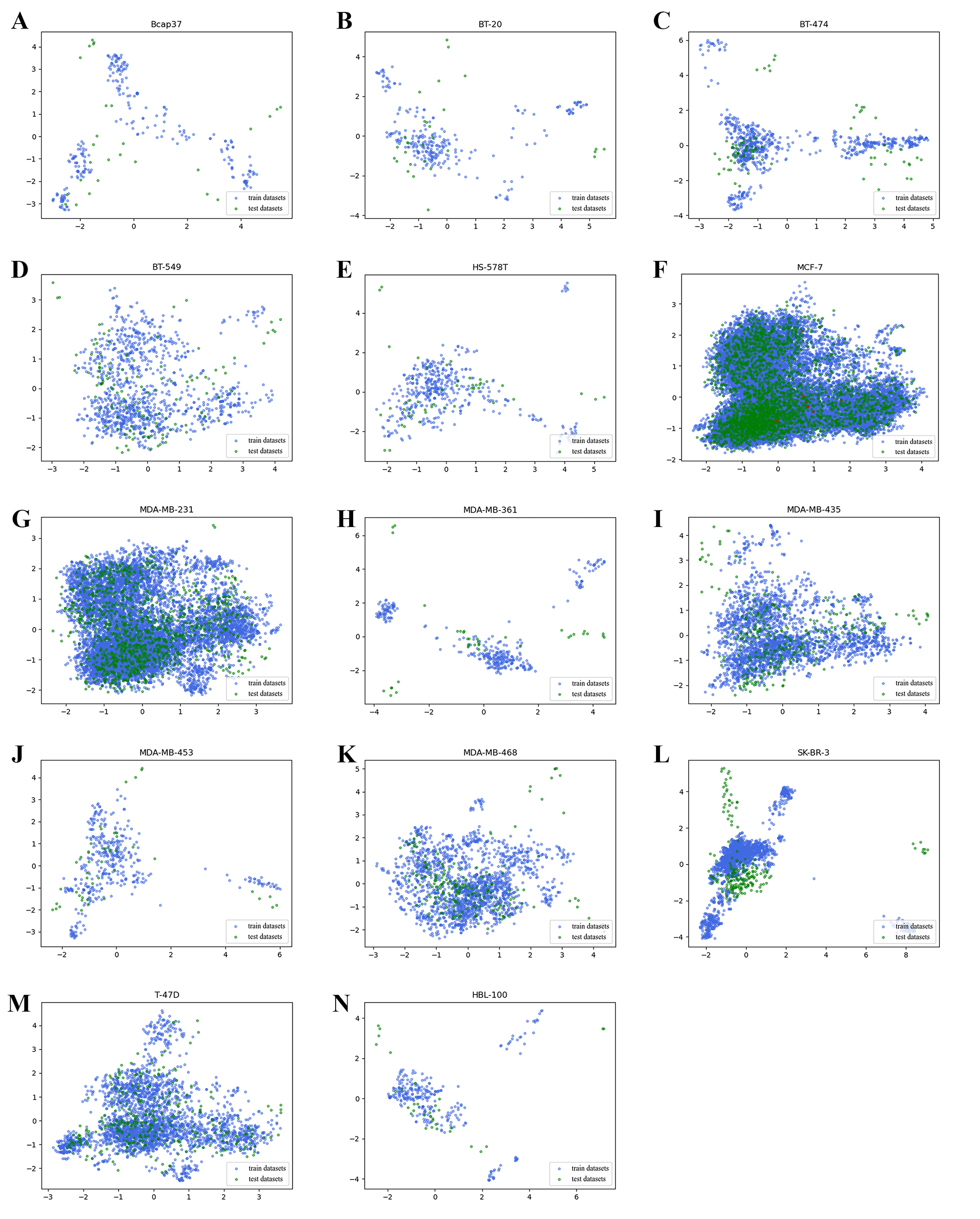


**Supplementary Figure S36.** Model AD in training sets and test sets in all breast cell lines. K was set to 5. By comparing the density of each point p and its five neighborhood points, whether this point is abnormal is judged. The lower the density of point p is, the more likely it is to be identified as an abnormal point. Exceptions are shown in red.
